# Invariance under quantum permutations rules out parastatistics

**DOI:** 10.1038/s41467-026-73064-6

**Published:** 2026-05-28

**Authors:** Manuel Mekonnen, Thomas D. Galley, Markus P. Müller

**Affiliations:** 1 Institute for Quantum Optics and Quantum Information, Austrian Academy of Sciences, Vienna, Austria; 2 Faculty of Physics, Vienna Center for Quantum Science and Technology (VCQ), University of Vienna, Vienna, Austria; 3 Perimeter Institute for Theoretical Physics, Waterloo, ON Canada

**Keywords:** Quantum information, Theoretical physics

## Abstract

Quantum systems invariant under particle exchange are either Bosons or Fermions, even though quantum theory in principle admits more general behavior under permutations. But why do we not observe such paraparticles in nature? The analysis of this question was previously limited primarily to specific quantum field theory models. Here we give two distinct model-independent arguments that rule out parastatistics, i.e. fundamentally indistinguishable quantum systems transforming under higher-dimensional representations of the symmetric group, which draw on quantum information theory and recent research on internal quantum reference frames. First, we introduce a notion of complete invariance: quantum systems should not only preserve their local state under permutations, but also the quantum information they carry about other systems, in analogy to the notion of complete positivity in quantum information theory. Second, we demand that quantum systems are invariant under quantum permutations, i.e. permutations conditioned on values of permutation-invariant observables. For both, we show that the respective principle is fulfilled if and only if the particle is a Boson or Fermion. Our results show how quantum reference frames can shed light on a longstanding problem of quantum physics, they underline the crucial role played by the compositional structure of quantum information, and demonstrate the explanatory power but also subtle limitations of recently proposed quantum covariance principles.

## Abstract (web summary)

The current understanding of why observed particles are only fermionic or bosonic under particle exchange is still incomplete. Here, the authors propose a modification of permutation invariance which encompasses quantum information invariance and quantum permutations, and show that, if one imposes such criteria, parastatistics is ruled out and only bosonic and fermionic statistics are left.

## Introduction

It is a fundamental empirical finding that all known particles in our universe are either Bosons or Fermions. But why is this the case? A standard but incomplete argument sometimes presented in the textbooks is as follows. Relabeling of identical particles should preserve all physical predictions. Thus, given a wavefunction *ψ*(*x*_1_,…,*x*_*N*_) for *N* particles, exchanging, say, the first two particles should only result in a global phase,

1 $$\psi ({x}_{2},{x}_{1},{x}_{3},\ldots,{x}_{N})={e}^{i\theta }\psi ({x}_{1},{x}_{2},\ldots,{x}_{N}),$$

which implies that the wavefunction transforms under a one-dimensional representation of the permutation group. It is either symmetric, *e*^*iθ*^ = +1 (as for Bosons), or antisymmetric, *e*^*iθ*^ = −1 (as for Fermions). This requirement is sometimes known as the symmetrization postulate^1^.

However, it has been understood since the 1950s^2^ that quantum theory admits more general behaviors of quantum systems under particle exchange. After all, general quantum states are represented by density operators, not state vectors, and these can preserve their form under actions of the permutation group which are more general than multiplication by a complex phase. This would lead to neither Bosonic nor Fermionic statistics, but parastatistics. Indeed, hypothetical paraparticles have been extensively studied over the last few decades^2–11^.

A widely accepted consequence of this research has been termed the equivalence thesis: every consistent theory of paraparticles is physically equivalent to some theory of regular Bosons or Fermions^12^. However, this conclusion has only been obtained under significant assumptions^13^, such as a locality-inspired charge recombination principle formulated in the framework of quantum field theory (QFT). Indeed, recent works have theoretically reinforced the consistent possibility of parastatistics^14,15^, including in certain quantum many-body systems^16,17^, which evade the usual QFT formalism and its locality principles. It is thus natural to ask: is parastatistics excluded by natural arguments that do not rely on (but still apply in a special case to) the framework of QFT?

We answer this question in the affirmative by reconsidering the basic question of what it even means that a quantum system is permutation-invariant. The standard notion says that all observables of the multi-particle system should remain invariant if the particles are exchanged according to a fixed permutation *π*. Implementing this unitarily, *π* ↦ *U*(*π*), leads to Bosonic and Fermionic, but also parastatistics. This term has been used to refer to other important families of particles with exotic exchange statistics such as anyons, which transform under the braid group, emergent parastatistics from ordinary Bosons and Fermions, and paraparticles where the invariant permutation action does more than just swapping modes, as in graded systems described by Lie superalgebras^14^.

In this work, we rule out parastatistics arising from fundamentally indistinguishable quantum systems transforming under multidimensional irreducible representations of the symmetric group. While not applying to parastatistics in emergent, anyonic and graded systems (as we will discuss), our results are rather general because they apply to all kinds of quantum systems that can be permuted, not only to particles with spatiotemporal locations. To this end, our approach defines permutation invariance directly at the level of Hilbert space operators, and not on the level of classical configuration space^18^. We take guidance from quantum information theory and research on quantum reference frames, suggesting two important aspects of permutation-invariance that have, to the best of our knowledge, not been considered in this context.

**Fig. 1:**
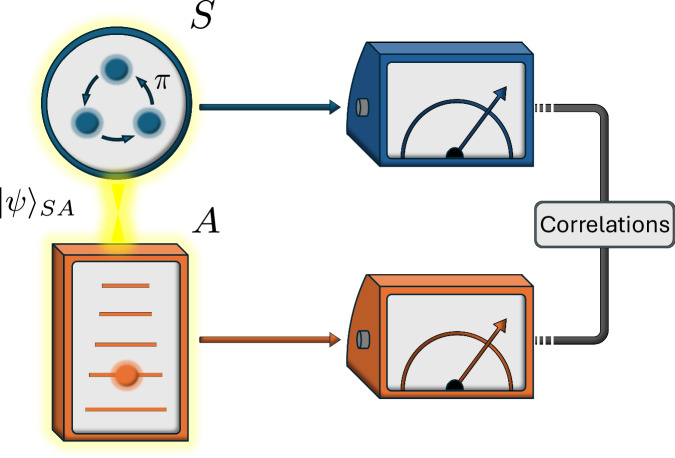
Complete invariance. All physical predictions are invariant under permutations *π* of the system of particles *S*. This includes the statistics and correlations of measurements of *S* and any ancillary system *A*, even if *SA* is potentially in a pure entangled state ${| \psi \rangle }_{SA}$.

The first aspect is that a quantum system *S* (say, of *N* indistinguishable particles) is more than its local, permutation-invariant state: it may contain quantum information about other systems *A* in the form of entanglement, which should also remain invariant under permutations on *S* (see Fig. 1)—even if *SA* is potentially in a pure entangled state. As we show, if we assume that any total state on *SA* is invariant under permuting *S* locally, then this more general form of invariance, which we call complete invariance, singles out Bosonic and Fermionic statistics.

**Fig. 2:**
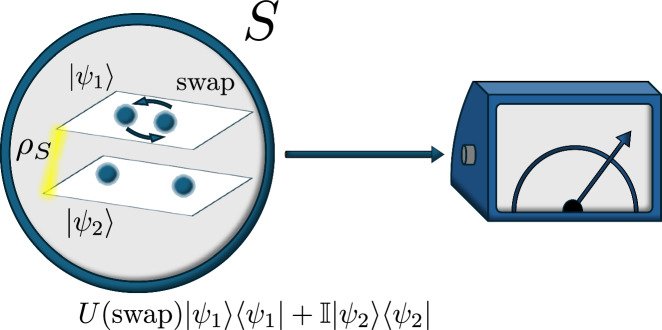
Invariance under quantum permutations. The system of particles *S* with state *ρ*_*S*_ in some superposition of $| {\psi }_{1}\rangle$ and $| {\psi }_{2}\rangle$ is permuted branchwise, i.e., conditioned on the value of a permutation-invariant observable. For example, we could apply *U*(swap) and swap the particles or apply the identity ${\mathbb{I}}$ and do nothing, depending on their distance. This must be possible in a way that preserves the statistics of all measurements on the system. We also consider what kinds of correlations with ancillary systems *A* are allowed by global invariance of *SA*.

A second aspect is that all physical predictions should remain invariant even if the system of particles *S* is permuted branch-wise, i.e., if the permutation *π* is chosen conditionally on the value of some permutation-invariant observable. For example, we could decide to swap two particles, or to do nothing, depending on the value of their distance *d*, see Fig. 2. While classically equivalent to standard permutation-invariance, we show that invariance under such quantum permutations is respected by Bosonic and Fermionic, but not by parastatistics.

Furthermore, since we are able to understand quantum permutations as quantum reference frame (QRF) transformations, our results relate in a surprising way to the field of QRFs. There, the idea is to extend the notion of reference frames, and of transformations between them, to quantum theory^19–43^. In this picture, invariance under standard permutations reflects the lack of a fundamental reference frame for labeling particles, while quantum permutation-invariance expresses the absence of a fundamental QRF for labeling.

## Results

### Standard permutation invariance and parastatistics

The notion of indistinguishability of particles in quantum physics describes the fact that particles with identical properties cannot be identified uniquely in principle, contrary to classical intuition. This means that shuffling *N* particles, either passively by changing the labels in their description, or actively by exchanging them via prescribed physical operations, must preserve all physical predictions.

Capturing this in mathematical terms, we associate to each way of shuffling *N* systems an element *π* in the permutation group ${{{{\mathcal{S}}}}}_{N}$. On a given Hilbert space ${{{\mathcal{H}}}}$, we then have some unitary representation *π* ↦ *U*(*π*) of this group, depending on the formalism we use to describe indistinguishable particles. For example, in first quantization, a single particle is described by a Hilbert space ${{{{\mathcal{H}}}}}_{1}$ such that the total Hilbert space is given by ${{{\mathcal{H}}}}={{{{\mathcal{H}}}}}_{1}^{\otimes N}$. An element *U*(*π*) then acts by permuting these *N* tensor factors. For instance, in the case of *N* = 2, the swap operation *U*(swap) would map a state $| \psi \rangle \otimes | \phi \rangle$ to $| \phi \rangle \otimes | \psi \rangle$. In second quantization, and in particular in the formalism of Green’s paraparticles that we include in our analysis, this tensor product structure is lost, and so are the aforementioned permutations of tensor factors. Indeed, attempts to define analogous transformations lead to non-unitary and non-physical maps (see Section F in Supplementary Note 4). Instead, individual systems to be shuffled are associated to modes *k*_*i*_, representing a property such as position or momentum, which can be occupied by some number ${n}_{{k}_{i}}$ of indistinguishable particles. Bosons (Fermions) are described by a Fock space ${{{\mathcal{F}}}}$ generated by creation operators ${a}_{{k}_{i}}^{{{\dagger}} }$ that fulfill the usual (anti)commutation relations, or more general trilinear relations in the case of paraparticles^1,2^, see Section B in Supplementary Note 2. The *N*-particle subspace ${{{{\mathcal{F}}}}}_{N}$ of the Fock space is spanned by vectors

2 $${| {k}_{1},\ldots,{k}_{N}\rangle }_{{{{\mathcal{F}}}}}:={a}_{{k}_{1}}^{{{\dagger}} }\ldots {a}_{{k}_{N}}^{{{\dagger}} }{| 0\rangle }_{{{{\mathcal{F}}}}},$$

where ${| 0\rangle }_{{{{\mathcal{F}}}}}$ is the vacuum state. Here, an element *U*(*π*) represents a physical transport operation^44^ that permutes the modes, while it acts mathematically by permuting the labels of the modes, and thus the associated creation operators, via $U(\pi ){a}_{{k}_{i}}^{{{\dagger}} }U{(\pi )}^{{{\dagger}} }={a}_{{k}_{\pi (i)}}^{{{\dagger}} }$.

Regardless of the formalism, the concept of indistinguishability can then be formalized by requiring permutation-invariance for all physical states: A state *ρ* of indistinguishable particles is physically allowed if

3 $$U(\pi )\rho U{(\pi )}^{{{\dagger}} }=\rho$$

holds for any permutation *π*. In second quantization we will only consider situations where we have *N* distinct modes with each mode occupied by one particle. On the one hand, this is a necessary restriction to make: if the modes have different occupation numbers then the physical systems they represent are simply distinguishable. On the other hand, this restriction still allows for the possibility of physically interesting parastatistical behavior, such as relative (2*π*/3)-phase shifts under coherently controlled permutations (see Example 3 in Supplementary Note 2) that are impossible for Bosons and Fermions^44^.

As a direct consequence of Schur’s lemma (see the “Methods” for details), permutation-invariant states decompose into a direct sum

4 $$\rho {=\bigoplus }_{\lambda }{\sigma }_{\lambda },$$

where the (subnormalized) density matrices *σ*_*λ*_ are each contained in invariant subspaces ${{{{\mathcal{H}}}}}_{\lambda }$, known as superselection sectors. The different sectors, labeled by the distinct irreducible representations (irreps) *λ* of *U*(*π*), correspond to different particle types. Bosonic (Fermionic) states are contained in the symmetric (antisymmetric) subspace of ${{{\mathcal{H}}}}$ and fulfill $U(\pi )| b\rangle=| b\rangle$ ($U(\pi )| \,f\rangle={{{\rm{sgn}}}}(\pi )| \,f\rangle$), thus transforming according to the trivial (sign) irrep of ${{{{\mathcal{S}}}}}_{N}$. All other irreps of the permutation group denote exchange statistics which is neither Bosonic nor Fermionic, known as parastatistics and obeyed e.g., by paraparticles. For examples of Bosonic, Fermionic and parastatistical states in first and second quantization formalisms, see Supplementary Note 2.

### Complete permutation invariance

So far, we have been considering any system *S* of *N* indistinguishable particles in isolation: implementing invariance on the level of states, i.e., demanding $U{(\pi )}_{S}{\rho }_{S}U{(\pi )}_{S}^{{{\dagger}} }={\rho }_{S}$, led to the form of *ρ* = *ρ*_*S*_ above, which includes the possibility of parastatistics. However, *S* will typically be part of a larger quantum system *SA*, consisting of itself and some ancillary system *A*, which may be a different type of system on which the permutations do not act. Then, the quantum state *ρ*_*S*_ will be the local reduced state of an extension *ρ*_*SA*_, i.e., of a quantum state that satisfies ${\rho }_{S}={{{{\rm{Tr}}}}}_{A}{\rho }_{SA}$. The assumption that this global state is invariant under local permutations of *S* means

5 $$\left(U{(\pi )}_{S}\otimes {{\mathbb{I}}}_{A}\right){\rho }_{SA}\left(U{(\pi )}_{S}^{{{\dagger}} }\otimes {{\mathbb{I}}}_{A}\right)={\rho }_{SA}.$$

In analogy with the notion of complete positivity from quantum information theory, we will say that *ρ*_*S*_ is completely invariant if Eq. (5) holds for all possible extensions *ρ*_*SA*_. Complete invariance implies invariance, but the converse is not in general true.

It turns out that complete invariance follows already from the existence of a single pure state ${\rho }_{SA}=| \psi \rangle {\langle \psi | }_{SA}$, i.e., a purification^45^ of *ρ*_*S*_, that satisfies Eq. (5). In this case, Eq. (5) can be interpreted as saying that not only is the local state of *S* preserved under permutations (as in standard invariance), but so is the quantum information that it carries about other systems *A*.

It turns out to be the case that complete invariance is a crucial difference between ordinary statistics and parastatistics. Requiring this property rules out the latter:

#### Theorem 1

A quantum state *ρ*_*S*_ is completely invariant under permutations if and only if it is fully supported on either the Bosonic or the Fermionic subspace.

The full proof of this is deferred to the “Methods” section, but in essence, it works by virtue of showing that complete invariance of a state *ρ*_*S*_ is equivalent to the existence of a single purification ${| \psi \rangle }_{SA}$ of *ρ*_*S*_ which is invariant under local permutations $U{(\pi )}_{S}\otimes {{\mathbb{I}}}_{A}$ on *S*. Furthermore, given any invariant purification, we find that its marginal on *S* must be either a Bosonic or Fermionic state, thus ruling out invariant purifications for paraparticles.

Besides being a natural notion from the perspective of quantum information theory, another possible motivation for complete invariance comes from the idea that the universe should always be in a pure quantum state, and that all mixed states of subsystems *S* should be understood as improper mixtures, arising from entanglement with the rest of the world *A*. This is in line with some versions of the many-worlds interpretation of quantum mechanics, and it resembles an attitude of quantum information theory that has been termed the church of the larger Hilbert space. Furthermore, complete invariance expresses a strong compatibility of symmetries and compositions of systems: if we have a system *S* in our lab, and we know that it is prepared in a completely invariant state (e.g., by measuring the projector onto the Bosonic or Fermionic subspace), we can be sure that nothing else in the universe will change under local permutations, regardless of how *S* is correlated or entangled with other systems *A*. Similarly as for complete positivity, a more detailed analysis of this notion could benefit from the category-theoretic methods of process theories, analyzing the compositional structure of quantum theory in a more systematic way.

While complete invariance is a natural requirement for fundamentally indistinguishable particles, it is not expected to hold in systems with emergent symmetries. This is illustrated by Example 4 in Supplementary Note 2 and Subsection D in Supplementary Note 3. Beyond permutations, it is natural to expect that complete invariance also holds for fundamental gauge symmetries in QFT. In Subsection E in Supplementary Note 3, we prove a generalization of Theorem 1: complete invariance under a compact group ${{{\mathcal{G}}}}$ is equivalent to the system *S* transforming under a one-dimensional representation of ${{{\mathcal{G}}}}$. Even though it is beyond our mathematical framework, note that Dirac quantization implements gauge symmetries via constraints such as $C| \psi \rangle=0$ (for example, the Gupta-Bleuler condition $C={\partial }^{\mu }{A}_{\mu }^{+}$ in quantum electrodynamics^46^). The gauge transformations *G* generated by *C* hence satisfy $G| \psi \rangle=| \psi \rangle$ (or, in more general Yang-Mills theories, $G| \psi \rangle={e}^{i\theta }| \psi \rangle$^47^), corresponding to one-dimensional representations, even if the gauge group is non-Abelian. Hence, if the mathematical framework can be suitably generalized, we expect complete invariance to hold here, confirming the natural intuition that it expresses nothing in the world to change whatsoever under gauge transformations.

### Quantum permutation invariance

We now consider another extension of standard permutation invariance which is naturally motivated by QRFs, as we explain further below: invariance under a quantum permutation group. In contrast to the previous argument, this one rules out parastatistics in both first and second quantization.

A quantum permutation can be thought of as a collection of permutations, each one acting independently on a different branch of a superposition—a coherently controlled, conditional permutation. For example, if we have *N* = 2 particles, we might swap them if their distance is less than *d*, or otherwise do nothing (see Fig. 2), as implemented by

6 $${V}_{+}=U({{{\rm{swap}}}})\cdot P+{\mathbb{I}}\cdot ({\mathbb{I}}-P),$$

where $U({{{\rm{swap}}}})| {x}_{1},{x}_{2}\rangle=| {x}_{2},{x}_{1}\rangle$, and $P| {x}_{1},{x}_{2}\rangle=| {x}_{1},{x}_{2}\rangle$ if ∣*x*_1_ − *x*_2_∣ < *d* and 0 otherwise. The two branches here are defined by the distance between a pair of particles. Generally, the branches can be defined by any projective measurement that is itself permutation-invariant. The projective action on the separate branches must be the same as that of the base representation *U*(*π*), and if we do the same permutation on each branch, ***π*** = (*π*,…,*π*), then we must obtain the projective action of *U*(*π*). This leads to the following definition, with an arbitrary phase *θ*(*π*) depending on the permutation *π*:

#### Definition 1

A quantum permutation is a unitary transformation of the form

7 $$V({{{\boldsymbol{\pi }}}})={\sum }_{j}{e}^{i\theta ({\pi }_{j})}U({\pi }_{j}){P}_{j},$$

where {*P*_*j*_} is a permutation-invariant projective measurement, i.e., [*U*(*π*), *P*_*j*_] = 0 for all *π* ∈ *S*_*N*_ and all *j*. If ***π*** ↦ *V*(***π***) is a projective representation of *S*_*N*_ × *S*_*N*_ × … × *S*_*N*_, then we call the set ${{{\mathcal{V}}}}$ of all *V*(***π***) a quantum permutation group. We call ${{{\mathcal{V}}}}$ maximal if the *P*_*j*_ have finite and smallest possible rank under these requirements.

Now, while all Bosonic states are invariant under *V*_+_, a quantum permutation such as

8 $${V}_{-}=-U({{{\rm{swap}}}})\cdot {{{\rm{P}}}}+{\mathbb{I}}\cdot ({\mathbb{I}}-{{{\rm{P}}}})$$

leaves all Fermionic states invariant. Moreover, we find that elements such as *V*_+_ and *V*_−_ are separately contained in different quantum permutation groups, each of which still leaves all Bosonic or all Fermionic states invariant. As it turns out, there exists no such group for all paraparticle states, leading to our main result ruling them out:

#### Theorem 2

For any system *S* of indistinguishable particles and any admissible {*P*_*j*_}, there exist exactly two quantum permutation groups ${{{{\mathcal{V}}}}}_{+}$ and ${{{{\mathcal{V}}}}}_{-}$, respectively containing elements of the following forms:

- *V*_+_(***π***) = ∑_*j*_*U*(*π*_*j*_)*P*_*j*_,
- $${V}_{-}({{{\boldsymbol{\pi }}}})={\sum }_{j}{{{\rm{sgn}}}}({\pi }_{j})U({\pi }_{j}){P}_{j}$$.

All Bosonic (Fermionic) states are invariant under ${{{{\mathcal{V}}}}}_{+}$ (${{{{\mathcal{V}}}}}_{-}$). In contrast, any paraparticle state invariant under either group must be diagonal in the basis {*P*_*j*_}. Hence, paraparticle systems invariant under any maximal quantum permutation group can neither be entangled nor interact unitarily with any ancillary system, in contrast to Bosonic and Fermionic systems.

Furthermore, if we require quantum permutation-invariance with respect to all possible projective measurements {*P*_*j*_} (which holds for Bosons and Fermions), then every paraparticle system must be completely uncorrelated with its environment. The proofs can be found in the “Methods” section. As a direct consequence, one finds that in the case of only considering classical states of indistinguishable particles, i.e., diagonal states in a given basis, requiring this conditional kind of permutation-invariance does not imply more restrictions than standard permutation-invariance. The two notions are thus classically equivalent. Tables 1 and 2 show the forms of invariant states under standard, quantum and strong quantum permutation-invariance, and the possible correlations with ancillary systems.

**Table 1:** General form of invariant and completely invariant states for the different symmetries {*U*(*π*)} ⊂ {*V*(*π*)} ⊂ ⋃_*P*_{*V*(*π*; *P*)} considered in this paper, where *V*(*π*; *P*) denotes either *V*_+_(*π*) or *V*_−_(*π*) defined relative to a projective measurement *P* = {*P*_*j*_}, and *V*(*π*) = *V*(*π*, *P*) for some fixed maximal *P*

Symmetry *V*(*g*)	*ρ*_*S*_ = *V*(*g*)*ρ*_*S*_*V*(*g*)^†^	*ρ*_*SA*_ pure, ${\rho }_{SA}=(V(g)\otimes {{\mathbb{I}}}_{A}){\rho }_{SA}{(V(g)\otimes {{\mathbb{I}}}_{A})}^{{{\dagger}} }$
*U*(*π*), *π* ∈ *S*_*N*_	${\rho }_{S}{=\bigoplus }_{\lambda }{p}_{\lambda }\frac{{\mathbb{I}}}{{d}_{\lambda }}\otimes {\rho }_{\lambda }$	*ρ*_*S*_ = *ρ*_*λ*_, *λ* ∈ {*λ*_Bos_, *λ*_Ferm_}
$V({{{\boldsymbol{\pi }}}}),{{{\boldsymbol{\pi }}}}\in {S}_{N}^{Q}$	${\rho }_{S}={p}_{\gamma }{\rho }_{\gamma }{+\bigoplus }_{\lambda \ne \gamma }{p}_{\lambda }\frac{{\mathbb{I}}}{{d}_{\lambda }}\otimes {\rho }_{\lambda }^{{{{\rm{diag}}}}}$, *γ* ∈ {*λ*_Bos_, *λ*_Ferm_}	*ρ*_*S*_ = *ρ*_*λ*_, *λ* ∈ {*λ*_Bos_, *λ*_Ferm_}
$V({{{\boldsymbol{\pi }}}};P),{{{\boldsymbol{\pi }}}}\in {S}_{N}^{Q},P\in {{{{\rm{Proj}}}}}_{{S}_{N}}$	${\rho }_{S}={p}_{\gamma }{\rho }_{\gamma }{+\bigoplus }_{\lambda \ne \gamma }{p}_{\lambda }\frac{{\mathbb{I}}}{{D}_{\lambda }}$, *γ* ∈ {*λ*_Bos_, *λ*_Ferm_}	*ρ*_*S*_ = *ρ*_*λ*_, *λ* ∈ {*λ*_Bos_, *λ*_Ferm_}

We use the notation ${\rho }_{\lambda }^{{{{\rm{diag}}}}}={\sum }_{i}{p}_{i,\lambda }{P}_{i,\lambda }$ for a quantum state diagonal in the basis of the measurement {*P*_*i*,*λ*_} controlling the quantum permutation, and ${{{{\rm{Proj}}}}}_{{S}_{N}}$ for the set of projective measurements where all projections commute with the representation of *S*_*N*_. This table shows that without appeal to an ancillary system, one can effectively rule out parastatistics by appealing to invariance under quantum permutations *V*(***π***) or *V*(***π***; *P*). By appealing to an ancilla and the existence of a pure global state *ρ*_*SA*_, invariance under the standard permutation group is enough to rule out parastatistics.

**Table 2:** Allowed correlations with the environment when imposing invariance on system *S* and ancilla *A* for the different symmetries considered in this paper

Symmetry ${V}_{S}(g)\otimes {{\mathbb{I}}}_{A}$	Correlations with ${{{{\mathcal{H}}}}}_{A}$ for *λ* ∈ {*λ*_Bos_, *λ*_Ferm_}	Correlations with ${{{{\mathcal{H}}}}}_{A}$ for *λ* ∉ {*λ*_Bos_, *λ*_Ferm_}	
	*ρ*_*SA*_ arbitrary	*ρ*_*SA*_ pure	*ρ*_*SA*_ arbitrary
*U*(*π*), *π* ∈ *S*_*N*_	Entanglement possible	No global state	Entanglement possible
$V({{{\boldsymbol{\pi }}}}),{{{\boldsymbol{\pi }}}}\in {S}_{N}^{Q}$	Entanglement possible	No global state	Classical correlations only
$V({{{\boldsymbol{\pi }}}};P),{{{\boldsymbol{\pi }}}}\in {S}_{N}^{Q},P\in {{{{\rm{Proj}}}}}_{{S}_{N}}$	Entanglement possible	No global state	No correlations

Requiring that *ρ*_*SA*_ is pure and invariant under the standard permutations *V*(*π*) is enough to rule out parastatistics. If *ρ*_*SA*_ is not required to be pure, then invariance under quantum permutations {*V*(***π***)} or ⋃_*P*_{*V*(***π***, *P*)} rules out parastatistics.

For the remainder of this section, we will relate our results to the field of QRFs. Initiated decades ago^19–22^ it has recently received a significant amount of attention in quantum information, quantum foundations, and quantum gravity research^23–43^. We will now argue that quantum permutations can be understood as QRF transformations. Since indistinguishable particles lack individual identity, there is no natural, physically preferred way to label them. Hence, there exists a relabeling group of elements *U*(*π*) preserving all physical predictions, and we can understand standard permutation invariance as the consequence of a lack of a fundamental reference frame for labeling. A choice of labeling then means breaking permutation-invariance and choosing a reference frame from which to describe the system: for example, we can identify the electron that is closer to Alice’s (Bob’s) laboratory as the “first (second) particle”.

Now, as a natural requirement strengthening this, we argue that there is also no physically preferred way to label particles—or to identify labeling conventions—across branches of superpositions. For example, what is labeled as “first particle” on one branch may be called “second particle” on another. Hence, there exists a quantum relabeling group preserving all physical predictions, and choices of labeling on each branch constitute a choice of a QRF.

QRF transformations in general are understood as coherently-controlled classical reference frame changes^30^, where the controlling branches are themselves defined in a frame-independent way^48^, or as state-dependent gauge transformations^49^. Hence, quantum permutations can be interpreted as QRF transformations. As abstract groups, they are *S*_*N*_ × *S*_*N*_ × … × *S*_*N*_ (one independent choice of permutation per branch), but the specific way they act on a Hilbert space depends on the type of physical system (via ${{{{\mathcal{V}}}}}_{+}$ for Bosons, via ${{{{\mathcal{V}}}}}_{-}$ for Fermions). This is a well-known general phenomenon in quantum physics: for example, electrons carry a different representation of the rotation group than photons. Indeed, it is well-known that applying an inappropriate quantum permutation group, such as ${{{{\mathcal{V}}}}}_{+}$ via active permutations to Fermions, will violate invariance and lead to physically detectable relative phases^44^. Hence, elements of ${{{{\mathcal{V}}}}}_{+}$ (understood either as active transformations or passive relabelings) are symmetry transformations for Bosons, but not for Fermions (and vice versa for ${{{{\mathcal{V}}}}}_{-}$ and Fermions). Similarly, for other types of systems without fundamental permutation invariance, e.g., anyons, none of the two groups would be symmetry transformations, and our results do not apply.

## Discussion

One of the goals of the QRF research program is to obtain novel physical predictions by postulating some version of covariance of the physical laws under QRF transformations^31,38,40,43^, such as the one formulated in ref. ^40^: “Physical laws retain their form under quantum coordinate transformations.” (for details see Section J in Supplementary Note 5). This extended covariance principle in the context of quantum gravity is but one example of a larger class of quantum generalizations of symmetry principles considered in the literature, including also, for example, proposals for a quantum version of the equivalence principle^31,38,50^, or a notion of quantum conformal symmetries^43^. Our result is an instance of this: postulating invariance under quantum permutations for fundamental particles predicts the empirically correct absence of parastatistics. While this supports the idea that such principles can be predictively powerful, it also motivates some caution: we show that whether or not a given quantum permutation is a QRF transformation depends on the type of system (here Bosonic or Fermionic) it acts on. Therefore, only some transformations will in general preserve the physical predictions for a given system, and we suggest the following modification of the postulate:

Physical laws retain their form under a suitable representation of the quantum coordinate transformation group.

Our results rule out fundamental parastatistics defined by multidimensional representations of the symmetric group. They do not exclude the possibility of emergent parastatistics, and hence do not stand in opposition to recent results showing the detectability of parastatistical correlations in many-body and quasiparticle systems^15–17^. In Example 4 in Supplementary Note 2 and Subsection D in Supplementary Note 3, we show that the intuitive reason for this is that permutations *U*(*π*) on the emergent system *S* are typically accompanied by non-trivial permutation actions on the complement of *S*, violating both our stronger notions of invariance in a natural way.

In principle, our mathematical results apply to all unitary representations of the symmetric group, enforcing that *U*(*π*) acts either trivially or with the sign representation on the physical states. If *U*(*π*) simply permutes the tensor factors or modes (as assumed above), then the corresponding symmetric and antisymmetric subspaces describe Bosons and Fermions, such that our results rule out parastatistics. However, if *U*(*π*) is a more exotic representation that e.g., couples the permutations to internal degrees of freedom, then these allowed states may still have an interpretation as particles with exotic exchange statistics. For example, this happens with ${{\mathbb{Z}}}_{2}\times {{\mathbb{Z}}}_{2}$-graded parastatistics^14^, where creation operators are exchanged with a grading-dependent phase ${\widehat{a}}^{{{\dagger}} }{\widehat{b}}^{{{\dagger}} }\mapsto {(-1)}^{\langle \alpha,\beta \rangle }{\widehat{b}}^{{{\dagger}} }{\widehat{a}}^{{{\dagger}} }$. Such systems transform trivially under exotic permutation actions and are therefore consistent with our principles of quantum permutation invariance and complete invariance as applied to those exotic permutation actions.

Our work raises a number of interesting follow-up questions. May complete invariance point towards a deeper principle related to composing subsystems consistently, in the presence of more abstract, structural properties? Can further transformation properties of fundamental particles be understood as consequences of quantum covariance principles? These future questions notwithstanding, we believe that our results provide a novel perspective on the nature of Bosons and Fermions and thus of the very building blocks of our universe.

## Methods

### Representation theory and permutation-invariance

Suppose that we have *N* ≥ 2 and a complex, separable Hilbert space ${{{{\mathcal{H}}}}}_{1}$ describing particles or modes, depending on which quantization formalism we use. We assume that the dimension of ${{{{\mathcal{H}}}}}_{1}$ is at least two, but at most countably-infinite, thus the total Hilbert space is ${{{\mathcal{H}}}}={{{{\mathcal{H}}}}}_{1}^{\otimes N}$. We then have some unitary representation *π* ↦ *U*(*π*) of the permutation group *S*_*N*_ whose irreps are labeled by Young diagrams (also called Young frames^51^) *λ*. The two special cases are 9 denoting one-dimensional representations of *S*_*N*_: the trivial representation and the sign representation, respectively. These correspond to Bosons and Fermions. All other Young frames *λ* correspond to irreps that are at least two-dimensional, and these describe irreps of *S*_*N*_ associated with paraparticles^2,52^. Since *S*_*N*_ is a finite group, its representation decomposes as

10 $$U(\pi ){=\bigoplus }_{\lambda }{U}_{\lambda }(\pi )\otimes {{\mathbb{I}}}_{{n}_{\lambda }},$$

with the Hilbert space decomposing as

11 $${{{\mathcal{H}}}}{=\bigoplus }_{\lambda }{{{{\mathcal{M}}}}}_{\lambda }\otimes {{{{\mathcal{N}}}}}_{\lambda }.$$

Here, ${n}_{\lambda }=\dim ({{{{\mathcal{N}}}}}_{\lambda })$ (which may be infinite) denotes the number of copies of the irrep *λ* and *U*_*λ*_(*π*) the representation matrix on the irrep subspace ${{{{\mathcal{M}}}}}_{\lambda }$. Since these abstract decompositions apply to all unitary representations of *S*_*N*_ on separable Hilbert spaces, our results will apply broadly to all of them.

Let us now postulate permutation-invariance: only quantum states *ρ* with *U*(*π*)*ρU*(*π*)^†^ = *ρ* are allowed to describe the results of physical preparation procedures. Hence, quantum states must be described by density operators that commute with all *U*(*π*) and are of the form

12 $$\rho {=\bigoplus }_{\lambda }{p}_{\lambda }\frac{{{\mathbb{I}}}_{\lambda }}{{d}_{\lambda }}\otimes {\rho }_{\lambda },$$

where *d*_*λ*_ is the dimension of the irrep *λ*, ${\{{p}_{\lambda }\}}_{\lambda }$ is a probability distribution, and the *ρ*_*λ*_ are density matrices. All decompositions above are the direct result of Lemma 4 in Supplementary Note 1.

### Formalism of complete invariance

Complete invariance dictates that physics is invariant under local permutations of *S*, even in the presence of an arbitrary ancillary system. Given a permutation-invariant state *ρ*_*S*_ on a system *S* of indistinguishable particles, it is expressed by the condition that all possible extensions *ρ*_*SA*_ with ${\rho }_{S}={{{\rm{Tr}}}}({\rho }_{SA})$ are invariant under local permutations, i.e.

13 $$\left({{\mathrm{U}}}{(\pi )}_{S}\otimes {{\mathbb{I}}}_{A}\right){\rho }_{SA}\left(U{(\pi )}_{S}^{{{\dagger}} }\otimes {{\mathbb{I}}}_{A}\right)={\rho }_{SA},$$

for all permutations *π* any ancillary system *A*.

The following lemma shows that this notion can be mathematically defined in different ways.

#### Lemma 1

The following conditions are all equivalent, and can be used to define what it means that a quantum state *ρ*_*S*_ is completely invariant under permutations:

(i) Eq. (13) holds for some purification *ρ*_*SA*_ of *ρ*_*S*_;
(ii) Eq. (13) holds for all purifications *ρ*_*SA*_ of *ρ*_*S*_;
(iii) Eq. (13) holds for all extensions *ρ*_*SA*_ of *ρ*_*S*_.

Moreover, all three are equivalent to

(iv) *ρ*_*S*_ has full support on the *λ*_Bos_- or *λ*_Ferm_-subspace.

#### Proof

Clearly, (*iii*) ⇒ (*ii*) ⇒ (*i*), and we will now show that (*i*) ⇒ (*iv*). We have the decomposition

$$U{(\pi )}_{S}\otimes {{\mathbb{I}}}_{A}{=\bigoplus }_{\lambda }{U}_{\lambda }(\pi )\otimes ({{\mathbb{I}}}_{{n}_{\lambda }}\otimes {{\mathbb{I}}}_{A}),$$

and so Lemma 4 in Supplementary Note 1 implies that ${\rho }_{SA}{=\bigoplus }_{\lambda }{p}_{\lambda }\frac{{{\mathbb{I}}}_{\lambda }}{{d}_{\lambda }}\otimes {\rho }_{\lambda,A}$, where every *ρ*_*λ*,*A*_ is a quantum state on ${{{{\mathcal{N}}}}}_{\lambda }\otimes A$. But if *ρ*_*SA*_ is a pure state, then *p*_*λ*_ > 0 is only possible if *d*_*λ*_ = 1, leaving the two possibilities ${\rho }_{SA}=| \psi \rangle {\langle \psi | }_{{\lambda }_{{{{\rm{Bos}}}}},A}$ or ${\rho }_{SA}=| \psi \rangle {\langle \psi | }_{{\lambda }_{{{{\rm{Ferm}}}}},A}$. Hence, *ρ*_*S*_ has full support on the *λ*-subspace, where either *λ* = *λ*_Bos_ or *λ* = *λ*_Ferm_.

Let us finally show that (iv) ⇒ (iii). Let *P*_*λ*_ be the orthogonal projector onto ${{{{\mathcal{M}}}}}_{\lambda }\otimes {{{{\mathcal{N}}}}}_{\lambda }$, and let *ρ*_*SA*_ be any extension of *ρ*_*S*_. We have $1={{{\rm{Tr}}}}({P}_{\lambda }{\rho }_{S})={{{\rm{Tr}}}}(({P}_{\lambda }\otimes {{\mathbb{I}}}_{A}){\rho }_{SA})$, and thus $({P}_{\lambda }\otimes {{\mathbb{I}}}_{A}){\rho }_{SA}({P}_{\lambda }\otimes {{\mathbb{I}}}_{A})={\rho }_{SA}$. Using that $U{(\pi )}_{S}{P}_{\lambda }={{{{\rm{sgn}}}}}_{\lambda }(\pi ){P}_{\lambda }$, where ${{{{\rm{sgn}}}}}_{\lambda }(\pi )={{{\rm{sgn}}}}(\pi )$ if *λ* = *λ*_Ferm_ and ${{{{\rm{sgn}}}}}_{\lambda }(\pi )=1$ if *λ* = *λ*_Bos_, we obtain

$$\begin{array}{rcl} & & U{(\pi )}_{S}\otimes {{\mathbb{I}}}_{A}{\rho }_{SA}U{(\pi )}_{S}^{{{\dagger}} }\otimes {{\mathbb{I}}}_{A}\\ & &=(U{(\pi )}_{S}\otimes {{\mathbb{I}}}_{A})({P}_{\lambda }\otimes {{\mathbb{I}}}_{A}){\rho }_{SA}({P}_{\lambda }\otimes {{\mathbb{I}}}_{A})\left(U{(\pi )}_{S}^{{{\dagger}} }\otimes {{\mathbb{I}}}_{A}\right)\\ & &={\rho }_{SA},\end{array}$$

and so *ρ*_*SA*_ satisfies (13).

This implies Thm. 1 that rules out parastatistics in first quantization.

### Formalism of quantum permutations

Starting from the definition given in the main text, we can give a more explicit form of the quantum permutations. Recall the decomposition of the total Hilbert space ${{{\mathcal{H}}}}{=\bigoplus }_{\lambda }{{{{\mathcal{M}}}}}_{\lambda }\otimes {{{{\mathcal{N}}}}}_{\lambda }$, on which the permutations act as $U(\pi ){=\bigoplus }_{\lambda }{U}_{\lambda }(\pi )\otimes {{\mathbb{I}}}_{\lambda }$. By Schur’s Lemma, projective measurements commuting with all those unitaries are of the form

14 $${\{{{\mathbb{I}}}_{\lambda }\otimes {P}_{\lambda,j}\}}_{\lambda,j},$$

and this will define a maximal quantum permutation group if the *P*_*λ*,*j*_ are all rank-one projectors. Such maximally finely-controlled quantum permutations are hence of the form

15 $$\begin{array}{rcl}V({{{\boldsymbol{\pi }}}}) &=& {\sum }_{\lambda,j}{e}^{i\theta ({\pi }_{\lambda,j})}U({\pi }_{\lambda,j})({{\mathbb{I}}}_{\lambda }\otimes {P}_{\lambda,j})\\ &=& {\sum }_{\lambda,j}{e}^{i\theta ({\pi }_{\lambda,j})}{U}_{\lambda }({\pi }_{\lambda,j})\otimes {P}_{\lambda,j}\end{array}$$

with ${{{\rm{Tr}}}}({P}_{\lambda,j})=1$. Note that we always have at least two particles (or modes) with associated Hilbert spaces of dimensions at least two, and so the number *Q* of possible (*λ*, *j*) pairs is at least two. In what follows, we will hence always assume that *Q*≥2. In the case of infinite-dimensional Hilbert spaces, we will restrict our attention to finite-rank projections for simplicity.

In the following lemma, let us now prove the first part of our main theorem: the existence of exactly two quantum permutation groups.

#### Lemma 2

The map ***π*** ↦ *V*(***π***) in Eq. (15) defines a quantum permutation group if and only if either ${e}^{i\theta ({\pi }_{\lambda,j})}={e}^{i\theta }$ or ${e}^{i\theta ({\pi }_{\lambda,j})}={e}^{i\theta }{{{\rm{sgn}}}}({\pi }_{\lambda,j})$ for some fixed *θ* (which we will in the following, without loss of generality, set to zero). In both cases, if we set *θ* to zero, it is not only a projective, but a linear representation of ${S}_{N}^{Q}$.

#### Proof

The map ***π*** ↦ *V*(***π***) is a projective representation if and only if there are complex numbers *ω*(***σ***, ***π***) such that *V*(***σ***)*V*(***π***) = *ω*(***σ***, ***π***)*V*(***σπ***). Direct calculation shows that this implies

$$\omega ({{{\boldsymbol{\sigma }}}},{{{\boldsymbol{\pi }}}})={e}^{i\theta ({\sigma }_{\lambda,j})}{e}^{i\theta ({\pi }_{\lambda,j})}{e}^{-i\theta ({\sigma }_{\lambda,j}{\pi }_{\lambda,j})}\,\,{{{\rm{for\; all}}}}\,\lambda,j.$$

Suppose that ${{{{\boldsymbol{\sigma }}}}}^{{\prime} }$ and ${{{{\boldsymbol{\pi }}}}}^{{\prime} }$ are elements of ${S}_{N}^{Q}$ that agree with ***σ*** and ***π*** on at least one entry (*λ*, *j*), i.e., there exists some (*λ*, *j*) such that ${\sigma }_{\lambda,j}={\sigma }_{\lambda,j}^{{\prime} }$ and ${\pi }_{\lambda,j}={\pi }_{\lambda,j}^{{\prime} }$. Then it follows that $\omega ({{{\boldsymbol{\sigma }}}},{{{\boldsymbol{\pi }}}})=\omega ({{{{\boldsymbol{\sigma }}}}}^{{\prime} },{{{{\boldsymbol{\pi }}}}}^{{\prime} })$. Let ***σ***^*″*^, ***π***^*″*^ be arbitrary elements of ${S}_{N}^{Q}$. Then we can always find some pair ${{{{\boldsymbol{\sigma }}}}}^{{\prime} },{{{{\boldsymbol{\pi }}}}}^{{\prime} }$ that agree with the pair ***σ***, ***π*** in at least one entry, and that also agrees with the pair ***σ***^*″*^, ***π***^*″*^ in at least one entry. Thus, *ω*(***σ***, ***π***) = *ω*(***σ***^*″*^, ***π***^*″*^) = *ω* is a constant that does not depend on ***σ*** or ***π***. The special case ${\pi }_{\lambda,j}={\mathbb{I}}$ shows that *ω* = *e*^*iθ*^ for $\theta :=\theta ({\mathbb{I}})$, and hence ***π*** ↦ *e*^−*iθ*^*V*(***π***) is a linear unitary representation. Furthermore, *π* ↦ *e*^−*iθ*^*e*^*iθ*(*π*)^ is a linear one-dimensional representation of *S*_*N*_, i.e., either the trivial or the sign representation. This shows that *V*(***π***) is of the claimed form.

Thus, for every choice of one-dimensional projectors *P*_*λ*,*j*_ in the subspaces ${{{{\mathcal{M}}}}}_{\lambda }$, there are (up to a global phase) two maximal quantum permutation groups, as stated in Theorem 2: one where *e*^*iθ*(*π*)^ = 1 and thus

16 $${V}_{+}({{{\boldsymbol{\pi }}}})={\sum }_{\lambda,j}{U}_{\lambda }({\pi }_{\lambda,j})\otimes {P}_{\lambda,j},$$

and another one where ${e}^{i\theta (\pi )}={{{\rm{sgn}}}}(\pi )$ and thus

17 $${V}_{-}({{{\boldsymbol{\pi }}}})={\sum }_{\lambda,j}{{{\rm{sgn}}}}({\pi }_{\lambda,j}){U}_{\lambda }({\pi }_{\lambda,j})\otimes {P}_{\lambda,j}.$$

Note that we would have obtained the exact same result if we had started with an even more general definition of quantum permutations. In contrast to Definition 1 where the representation *V* of the symmetric group is fixed, we could even let the representation (and thus the associated complex phases) depend on the permutation-invariant variable, i.e., on the branch *j*,

18 $$V({{{\boldsymbol{\pi }}}}):={\sum }_{j}{e}^{i{\theta }_{j}({\pi }_{j})}U({\pi }_{j}){P}_{j}.$$

In Section G in Supplementary Note 4, we show that this more general definition leads to the same conclusions as Definition 1.

Now, in order to derive the form of states invariant under quantum permutations and thus to show the rest of our main result Theorem 2, we will first need to establish the in-equivalence of representations on invariant subspaces for quantum permutations in Lemma 3 below.

Recall the decomposition of the Hilbert space Eq. (11), and rewrite it slightly as

19 $${{{\mathcal{H}}}}{=\bigoplus {}_{\lambda }\bigoplus }_{j}{{{{\mathcal{H}}}}}_{\lambda,j},$$

where every ${{{{\mathcal{H}}}}}_{\lambda,j}$ is an irreducible subspace for the representation *U*(*π*) of Eq. (10); concretely, ${{{{\mathcal{H}}}}}_{\lambda,j}={{{\rm{im}}}}({{\mathbb{I}}}_{\lambda }\otimes {P}_{\lambda,j})$. It is immediate from Eq. (16) and Eq. (17) that the ${{{{\mathcal{H}}}}}_{\lambda,j}$ are invariant subspaces for the quantum permutation groups *V*(***π***). Since subspaces invariant under all *V*(***π***) must also be invariant under all *U*(*π*) = *e*^−*iθ*(*π*)^*V*(*π*, …, *π*), the spaces ${{{{\mathcal{H}}}}}_{\lambda,j}$ cannot be decomposed any further into smaller-dimensional irreducible subspaces.

Thus, for both representations *π* ↦ *U*(*π*) and ***π*** ↦ *V*(***π***), we have an identical decomposition Eq. (19) into irreducible subspaces. However, for the former, every pair of subspaces ${{{{\mathcal{H}}}}}_{\lambda,j}$ and ${{{{\mathcal{H}}}}}_{\lambda,k}$ carries equivalent representations of the permutation group, which is not true for the group of quantum permutations:

#### Lemma 3

If *λ* ≠ *μ*, then ${{{{\mathcal{H}}}}}_{\lambda,j}$ and ${{{{\mathcal{H}}}}}_{\mu,k}$ carry inequivalent representations of the quantum permutation group $V({{{\boldsymbol{\pi }}}})\simeq {S}_{N}^{Q}$. Moreover, if *j* ≠ *k*, then ${{{{\mathcal{H}}}}}_{\lambda,j}$ and ${{{{\mathcal{H}}}}}_{\lambda,k}$ carry inequivalent representations of ${S}_{N}^{Q}$ in all cases except for the following two:

- *λ* = *λ*_Bos_ and *V*(***π***) = *V*_+_(***π***),
- *λ* = *λ*_Ferm_ and *V*(***π***) = *V*_−_(***π***).

#### Proof

Denote the action of *V*(***π***) on the invariant subspace ${{{{\mathcal{H}}}}}_{\lambda,j}$ by *V*_*λ*,*j*_(***π***). Suppose that *μ* ≠ *λ*, but that the representations ***π*** ↦ *V*_*λ*,*j*_(***π***) and ***π*** ↦ *V*_*μ*,*k*_(***π***) are equivalent. Then there is a unitary *W* such that *V*_*λ*,*j*_(***π***) = *WV*_*μ*,*k*_(***π***)*W*^†^. The special case ***π*** = (*π*, …, *π*) implies *U*_*λ*_(*π*) = *WU*_*μ*_(*π*)*W*^†^, and this can only hold for all *π* ∈ *S*_*N*_ if *λ* = *μ*, which is a contradiction.

Now let *j* ≠ *k*, and suppose that ${{{{\mathcal{H}}}}}_{\lambda,j}$ and ${{{{\mathcal{H}}}}}_{\lambda,k}$ carry equivalent representations of ${S}_{N}^{Q}$. Then there is some unitary *W* such that *V*_*λ*,*j*_(***π***) = *WV*_*λ*,*k*_(***π***)*W*^†^ for all ***π***, or equivalently, *ω*(*π*_*j*_)*U*_*λ*_(*π*_*j*_) = *ω*(*π*_*k*_)*WU*_*λ*_(*π*_*k*_)*W*^†^ for all *π*_*j*_, *π*_*k*_ ∈ *S*_*N*_, where by Lemma 2 *ω*(*π*) = 1 for all *π* if *V*(***π***) = *V*_+_(***π***), or $\omega (\pi )={{{\rm{sgn}}}}(\pi )$ for all *π* if *V*(***π***) = *V*_−_(***π***). For the special case ${\pi }_{k}={\mathbb{I}}$, this implies ${U}_{\lambda }(\pi )=\omega (\pi ){\mathbb{I}}$ for all *π* ∈ *S*_*N*_, and so *U*_*λ*_ corresponds to the trivial representation acting on the Bosonic subspace in the case *V*(***π***) = *V*_+_(***π***), or the sign representation acting on the Fermionic subspace in the case *V*(***π***) = *V*_−_(***π***). In both cases, *U*_*λ*_ is a one-dimensional representation acting on the Bosonic/Fermionic subspace respectively.

Let us now analyze the consequences of quantum permutation invariance. We will denote the *N* indistinguishable particles by *S* (the system), and a possible additional quantum system (the ancilla) by *A*. To consider *S* in isolation, we can simply treat *A* as a trivial quantum system of Hilbert space dimension one. Let us note that, unlike complete invariance, we are not imposing that the global invariant state is pure (or equivalently that every extension must be invariant). Rather, our goal is to determine the set of all invariant extensions.

First, let us consider the case that the quantum permutation group is the one of Eq. (16), i.e., *V*(***π***) = *V*_+_(***π***). If we apply some quantum permutation on *S* and the identity map on *A*, the resulting transformation can be decomposed as

20 $${V}_{+}{({{{\boldsymbol{\pi }}}})}_{S}\otimes {{\mathbb{I}}}_{A}=({{\mathbb{I}}}_{{\lambda }_{{{{\rm{Bos}}}}}}\otimes {{\mathbb{I}}}_{A}){\oplus }{\bigoplus }_{j,\lambda \ne {\lambda }_{{{{\rm{Bos}}}}}}{V}_{\lambda,j}({{{\boldsymbol{\pi }}}})\otimes {{\mathbb{I}}}_{A},$$

and all the representations ***π*** ↦ *V*_*λ*,*j*_(***π***) in this equation are irreducible and pairwise inequivalent. Let us now implement the invariance of all physical predictions under *V*_+_(***π***) on the level of states, and postulate that all allowed states are invariant under all these quantum coordinate transformations. Using Schur’s Lemma again (see Lemma 4 in Supplementary Note 1), the requirement $[{V}_{+}{({{{\boldsymbol{\pi }}}})}_{S}\otimes {{\mathbb{I}}}_{A},{\rho }_{SA}]=0$ for all ${{{\boldsymbol{\pi }}}}\in {S}_{N}^{Q}$ implies

21 $${\rho }_{SA}={p}_{{{{\rm{Bos}}}}}\,{\rho }_{{{{\rm{Bos}}}},A}{\oplus } {\bigoplus } _{j,\lambda \ne {\lambda }_{{{{\rm{Bos}}}}}}{p}_{\lambda,j}\frac{{{\mathbb{I}}}_{\lambda,j}}{{d}_{\lambda }}\otimes {\rho }_{A}^{(\lambda,j)},$$

where *d*_*λ*_ is the dimension of ${{{{\mathcal{H}}}}}_{\lambda,j}$ (which does not depend on *j*). Furthermore, *p*_Bos_ and the *p*_*λ*,*j*_ are probabilities that sum to one, *ρ*_Bos,A_ is a quantum state on ${{{{\mathcal{H}}}}}_{{{{\rm{Bos}}}}}\otimes A$, with ${{{{\mathcal{H}}}}}_{{{{\rm{Bos}}}}}$ the symmetric subspace of *S*, and the ${\rho }_{A}^{(\lambda,j)}$ are quantum states on *A*. This state describes a classical mixture of different types of statistics (for more details on how the *λ* are related to types of paraparticles such as parabosons and parafermions, see Supplementary Note 2). If the quantum number is measured and found to be *λ* = *λ*_Bos_, then the post-measurement state *ρ*_Bos,A_ can describe any quantum state whatsoever, and it may in particular be pure and entangled. However, if another type of statistics *λ* ≠ *λ*_Bos_ is found, then the post-measurement state will be

22 $${\rho }_{SA}(\lambda ){=\bigoplus }_{j}{p}_{j}\frac{{{\mathbb{I}}}_{\lambda,j}}{{d}_{\lambda }}\otimes {\rho }_{A}^{(\lambda,j)},$$

where *p*_*j*_ = *p*_*λ*,*j*_/∑_*j*_*p*_*λ*,*j*_. This is a separable state.

However, we can say more. Due to Eq. (22), all possible *ρ*_*SA*_(*λ*) are diagonal in the same basis of *S*, which is determined by the projective measurement Eq. (14). Thus, it is effectively a classical system. It is well-known that classical systems cannot reversibly interact with quantum systems in a nontrivial way. While stochastic evolution of *SA* under a Lindblad equation is possible^53^, no unitary time evolution generated by any nontrivial Hamiltonian *H*_*SA*_ ≠ *H*_*S*_ + *H*_*A*_ is possible: any consistent coupling between a classical and a quantum system must be fundamentally irreversible^54^. Essentially, if the particle is not a Boson, it cannot interact reversibly with any ancillary system. (Up to the fineprint that paraboson Hilbert spaces carry a Bosonic sector which may be entangled with other systems, but which, as the wording indicates, behaves exactly like a Boson. For the corresponding parafermion case, see Example 2 of Section B in Supplementary Note 2).

The second case of the quantum permutation group (as in Eq. (17)) can be treated analogously, starting from the decomposition

23 $${V}_{-}{({{{\boldsymbol{\pi }}}})}_{S}\otimes {{\mathbb{I}}}_{A}=({{\mathbb{I}}}_{{\lambda }_{{{{\rm{Ferm}}}}}}\otimes {{\mathbb{I}}}_{A}){\oplus }{\bigoplus } _{j,\lambda \ne {\lambda }_{{{{\rm{Ferm}}}}}}{V}_{\lambda,j}({{{\boldsymbol{\pi }}}})\otimes {{\mathbb{I}}}_{A}.$$

This establishes our main result.

Theorem 2 also shows that classically, invariance of probability distributions under conditional permutations is equivalent to invariance under unconditional ones. This follows from the fact that Theorem 2 does not yield any additional constraints in the case that all possible states and measurements are diagonal in the product basis.

If we assume the stronger form of quantum permutation invariance, we get an even stronger result:

#### Theorem 3

Consider a system *S* of indistinguishable particles, and assume strong quantum permutation invariance, i.e., invariance with respect to all maximal sets {*P*_*λ*,*j*_} of permutation-invariant projective measurements. Suppose that the particle type *λ* and the quantum permutations *V*(***π***) are among the following two cases:

- *λ* = *λ*_Bos_ and *V*(***π***) = *V*_+_(***π***) as in Eq. (16);
- *λ* = *λ*_Ferm_ and *V*(***π***) = *V*_−_(***π***) as in Eq. (17).

Then all quantum states of *S* are allowed. Moreover, *S* can be arbitrarily entangled with ancillary systems *A*.

In all other cases, however, and in particular if *λ* ∉ {*λ*_Bos_, *λ*_Ferm_}, the state of *S* must be maximally mixed:

$${\rho }_{S}=\frac{{{\mathbb{I}}}_{\lambda }}{{D}_{\lambda }},$$

where *D*_*λ*_ = *d*_*λ*_*n*_*λ*_ is the dimension of the paraparticle Hilbert subspace. Moreover, if we have an additional ancillary system *A*, then *S* and *A* must be uncorrelated:

$${\rho }_{SA}=\frac{{{\mathbb{I}}}_{\lambda }}{{D}_{\lambda }}\otimes {\rho }_{A},$$

and no continuous interaction whatsoever between *S* and *A* is possible. If one of the multiplicity spaces is infinite-dimensional, i.e., if there is some *λ* ∉ {*λ*_Bos_, *λ*_Ferm_} with *n*_*λ*_ = *∞*, then there does not exist any quantum state *ρ*_*S*_ (or *ρ*_*SA*_) with support on the corresponding subspace that satisfies strong quantum permutation invariance.

The proof of Theorem 3 is given in Section H in Supplementary Note 4. Furthermore, Tables 1 and 2 below show the forms of invariant states for the different symmetries, i.e., under standard, quantum and strong quantum permutation-invariance, and the possible correlations with ancillary systems in each case.

### Quantum reference frame descriptions

The invariance of all physical predictions under permutations can be implemented in three different ways. First, we can have invariance on the level of states, by postulating that only quantum states *ρ* with *U*(*π*)*ρU*(*π*)^†^ = *ρ* are allowed to describe the results of preparation procedures, without any restriction on the observables. Second, we can implement invariance on the level of observables, requiring that only operators *A* with *U*^†^(*π*)*AU*(*π*) = *A* can be measured, without any restriction on the states. Third, we can implement invariance on the level of states and observables, requiring that both lie in the subalgebra of operators commuting with all *U*(*π*). The three conventions are equivalent because

24 $${{{\rm{Tr}}}}[\widehat{P}(\rho )A]={{{\rm{Tr}}}}[\rho \widehat{P}(A)]={{{\rm{Tr}}}}[\widehat{P}(\rho )\widehat{P}(A)]$$

holds, where $\widehat{P}(X):=\frac{1}{| {S}_{N}| }{\sum }_{\pi \in {S}_{N}}U(\pi )XU{(\pi )}^{{{\dagger}} }$ projects every operator *X* into the subalgebra of invariant operators. These three conventions are also available for other symmetries, including symmetry under quantum permutations.

Now, while we have been working with the first convention so far, the second one is sometimes chosen implicitly in the context of QRFs: only observables invariant under the symmetry are deemed measurable, but all quantum states are allowed as descriptions of preparation procedures. There is an algebra of observables that can be measured under a certain condition of invariance, for example the subalgebra of all Bosonic operators ${{{{\mathcal{A}}}}}_{+}$ together with invariance under the group of Bosonic quantum permutations ${{{{\mathcal{V}}}}}_{+}$. For any state $\rho \in {{{\mathcal{S}}}}({{{\mathcal{H}}}})$, we then have ${{{\rm{Tr}}}}[\rho {A}_{+}]={{{\rm{Tr}}}}[{V}_{+}({{{\boldsymbol{\pi }}}})\rho {V}_{+}{({{{\boldsymbol{\pi }}}})}^{{{\dagger}} }{A}_{+}]$ for all ${V}_{+}({{{\boldsymbol{\pi }}}})\in {{{{\mathcal{V}}}}}_{+}$. This means that the set $\{{V}_{+}({{{\boldsymbol{\pi }}}})\rho {V}_{+}{({{{\boldsymbol{\pi }}}})}^{{{\dagger}} }| {V}_{+}({{{\boldsymbol{\pi }}}})\in {{{{\mathcal{V}}}}}_{+}\}$ contains alternative, equally valid descriptions of one and the same quantum state. Choosing one description over another can be interpreted as choosing a quantum coordinate system. Since quantum permutations in ${{{{\mathcal{V}}}}}_{+}$ map from one quantum coordinate system to another, they are viewed as QRF transformations for Bosons. In Section I in Supplementary Note 5 we provide a short introduction and more details on QRFs. In refs. ^33,37^, it is demonstrated that the paradigmatic QRF transformations of “jumping into the perspective of one of several particles” under translation-invariance can be understood in exactly this way.

## Supplementary information

Supplementary Information

Transparent Peer Review file
