# Supplementary material for: Invariance under quantum permutations rules out parastatistics
Source: Nat Commun. 2026 May 28;17:6947. doi: 10.1038/s41467-026-73064-6 (PMC13389154; doi:10.1038/s41467-026-73064-6)
Supplement: Supplementary file 1 — Supplementary Information [file 41467_2026_73064_MOESM1_ESM.pdf]

Supplementary Information for "Invariance under quantum permutations rules out parastatistics"

Manuel Mekonnen,[1,2] Thomas D. Galley,[1,2] and Markus P. Müller[1,2,3]

[1]*Institute for Quantum Optics and Quantum Information, Austrian Academy of Sciences, Boltzmanngasse 3, A-1090 Vienna, Austria*

[2]*Vienna Center for Quantum Science and Technology (VCQ), Faculty of Physics, University of Vienna, Boltzmanngasse 5, A-1090 Vienna, Austria*

[3]*Perimeter Institute for Theoretical Physics, 31 Caroline Street North, Waterloo, ON N2L 2Y5, Canada*

(Dated: April 30, 2026)

CONTENTS

Supplementary Note 1 – Consequences of Schur's Lemma 1

Supplementary Note 2 – Descriptions of parastatistics 2
- A Examples and classification 2
- B Parastatistics in second quantization 4
- C Example of emergent parastatistics 5

Supplementary Note 3 – Complete invariance 7
- D Failure of complete invariance for emergent paraparticles 7
- E Complete invariance for compact groups 7

Supplementary Note 4 – Structural details on quantum permutations 9
- F Applicability of our formalism in second quantization: particle permutations 9
- G More general quantum permutation groups 9
- H Proof of Theorem 3 in the main text 10

Supplementary Note 5 – Quantum Reference Frames 11
- I Application to Bosons and Fermions 11
- J Implications for quantum covariance principles 12

Supplementary Note 6 – Relation to other notions of symmetry in previous work 14
- K Strong versus weak and exact versus average symmetry 14
- L Complete invariance from local gauge symmetry 14
- M Global states in various approaches to quantum references frames 15

References 15

SUPPLEMENTARY NOTE 1 – CONSEQUENCES OF SCHUR'S LEMMA

In the following we consider unitary representations of compact (but not necessarily Lie) groups G on separable Hilbert spaces. We will always assume that G is, as a topological space, a Hausdorff space. We describe a consequence of Schur's Lemma [1, Proposition 5.8] that is used several times in the main text. We make use of the fact that unitary representations of compact groups are isomorphic to the Hilbert space direct sum of irreducible representations [2, Theorem 7.8], and irreducible representations of compact groups are finite-dimensional [2, Corollary 5.8].

The following is well-known, but we include the proof in our notation for completeness. For the basic representation-theoretic facts that we are using, we refer the reader to the books by Barry Simon [3] and Alain Robert [2]. Our two main cases of interest are the permutations and the quantum permutations, i.e. $\mathcal{G} = S_N$ and $\mathcal{G} = S_N^Q$, where Q denotes the number of branches, i.e. independent copies of S_N. If Q is not finite, but countably-infinite, then S_N^Q is still a compact group in the product topology due to Tychonoff's Theorem. Moreover, it is a totally disconnected group and hence topologically a Hausdorff space [1], i.e. a profinite group. The representation-theoretic results below hence apply to the case of $\mathcal{G} = S_N^\infty$, too.

Lemma 1. *Consider the unitary representation $U(g) = \bigoplus_{i\in N} U_\lambda^i(g)$, with N a discrete set, of a compact group G acting on $\mathcal{H} \simeq \bigoplus_{i\in N} \mathcal{H}_\lambda^i$, a direct sum of finite-dimensional Hilbert spaces $\mathcal{H}_\lambda^i$, where each irreducible component is isomorphic, then we have the following (group representation) isomorphism:*

$$\mathcal{H} \simeq \bigoplus_{i\in N} \mathcal{H}_\lambda^i \simeq \mathcal{M}_\lambda \otimes \mathcal{N}_\lambda, \tag{1}$$

where $\mathcal{M}_\lambda \simeq \mathcal{H}_\lambda$ and $\mathcal{N}_\lambda \simeq \ell^2(N)$ (which is isomorphic to $\mathbb{C}^n$ for N finite). The representation $U(g)$ acts as:

$$U(g) = \bigoplus_{i\in N} U_\lambda^i(g) \simeq U_\lambda(g) \otimes \mathbb{I}_{\mathcal{N}_\lambda}. \tag{2}$$

Proof. Denote ϕ_i the representation isomorphism $\mathcal{H}_\lambda^i \to \mathcal{M}_\lambda$, where $\mathcal{M}_\lambda$ carries the representation $U_\lambda(g)$, which exists since all $\mathcal{H}_\lambda^i$ are isomorphic. Define $\Phi_i : \mathcal{H}_\lambda^i \to \mathcal{M}_\lambda \otimes \mathcal{H}_i$ for $i \in N$ where $\mathcal{H}_i \simeq \mathbb{C}$:

$$\Phi_i : v^i \mapsto \phi_i(v^i) \otimes e_i, \tag{3}$$

where $v^i \in \mathcal{H}_\lambda^i$ and e_i a fixed unit vector in $\mathbb{C}$. The map Φ_i is a group representation isomorphism: $\Phi_i(U_\lambda^i(g)\bullet) = (U_\lambda(g)\otimes 1_i)\Phi_i(\bullet)$. Let $\Phi = \bigoplus_{i\in N}\Phi_i : \mathcal{H} \to \bigoplus_{i\in N}(\mathcal{M}_\lambda \otimes \mathcal{H}_i)$. This map is also a group representation isomorphism. Moreover,

$$\bigoplus_{i\in N}(\mathcal{M}_\lambda \otimes \mathcal{H}_i) \simeq \mathcal{M}_\lambda \otimes \bigoplus_{i\in N}\mathcal{H}_i = \mathcal{M}_\lambda \otimes \mathcal{N}_\lambda \tag{4}$$

where $\mathcal{N}_\lambda = \ell^2(N)$. The action of $U(g)$ under this isomorphism is $U_\lambda(g)\otimes \mathbb{I}_{\mathcal{N}_\lambda}$. □

Lemma 2. *Given a unitary reducible representation $U(g) = \bigoplus_{i=1}^n U_\lambda^i(g)$ acting on $\mathcal{H} \simeq \bigoplus_{i=1}^n \mathcal{H}_\lambda^i$ a direct sum of separable Hilbert spaces $\mathcal{H}_\lambda^i$, where each irreducible component is isomorphic, there exist unitary operators $W_{ij} : \mathcal{H}_\lambda^i \to \mathcal{H}_\lambda^j$ such that every operator A with $[A, U(g)] = 0$ can be written as*

$$A = \sum_{ij} a_{ij} W_{ij} \tag{5}$$

for some $a_{ij} \in \mathbb{C}$. Moreover, the W_{ij} are defined by the property $W_{ij}U_\lambda^j(g) = U_\lambda^i(g)W_{ij}$.

Proof. Define the orthogonal projector $\Pi_i : \mathcal{H} \to \mathcal{H}_\lambda^i$. Then since $\sum_i \Pi_i = \mathbb{I}_\mathcal{H}$ we can decompose A as:

$$A = \sum_{i,j}\Pi_i A \Pi_j = \sum_{ij} A_{ij}. \tag{6}$$

This is a block decomposition of A, and $[A, U(g)] = 0$ implies $A_{ij}U_\lambda^j(g) = U_\lambda^i(g)A_{ij}$. Hence, by Schur's Lemma, A_{ij} is a multiple of a unitary operator W_{ij} which is unique up to a constant which we can absorb into a_{ij}. □

Lemma 3. *Given a representation $U_\lambda(g)\otimes\mathbb{I}_{\mathcal{N}_\lambda}$ acting on $\mathcal{M}_\lambda\otimes\mathcal{N}_\lambda$, any operator A which commutes with $U_\lambda(g)\otimes\mathbb{I}_{\mathcal{N}_\lambda}$ is of the form:*

$$A = \mathbb{I}_{\mathcal{M}_\lambda}\otimes A_{\mathcal{N}_\lambda}. \tag{7}$$

Proof. We make use of the isomorphism $\phi : \bigoplus_i \mathcal{H}_\lambda^i \to \mathcal{M}_\lambda \otimes \mathcal{N}_\lambda$. According to Lemma 2, we have

$$W_{ki}W_{ij}U_\lambda^j(g) = W_{ki}U_\lambda^i(g)W_{ij} = U_\lambda^k(g)W_{ki}W_{ij}$$

and thus $W_{ki}W_{ij} = W_{kj}$. Thus, we can find bases $\{|e_k, i\rangle\}_k$ of every $\mathcal{H}_\lambda^i$ such that $W_{ij}|e_k, i\rangle = |e_k, j\rangle$. Hence, every invariant operator on $\bigoplus_i \mathcal{H}_\lambda^i$ is of the form:

$$A = \sum_{ij} a_{ij}\sum_k |e_k, i\rangle\langle e_k, j|. \tag{8}$$

Under the isomorphism ϕ this is mapped to:

$$(\phi\otimes\phi^*)(A) = \sum_{ij} a_{ij}\sum_k |e_k\rangle|i\rangle\langle e_k|\langle j| \tag{9}$$

$$= \sum_k |e_k\rangle\langle e_k| \otimes \sum_{ij} a_{ij}|i\rangle\langle j| \tag{10}$$

$$= \mathbb{I}_{\mathcal{M}_\lambda}\otimes A_{\mathcal{N}_\lambda}. \tag{11}$$

This shows that A is of the form as claimed. □

Lemma 4. *A unitary representation U of a compact group G on a separable Hilbert vector space $\mathcal{H}$ induces a decomposition:*

$$\mathcal{H} \simeq \bigoplus_\lambda \mathcal{M}_\lambda \otimes \mathcal{N}_\lambda, \tag{12}$$

where λ labels the irreducible representations of G. The action of $U(g)$ is

$$U(g) \simeq \bigoplus_\lambda U_{\mathcal{M}_\lambda}\otimes \mathbb{I}_{\mathcal{N}_\lambda}. \tag{13}$$

All invariant operators A are of the form:

$$A = \bigoplus_\lambda \mathbb{I}_{\mathcal{M}_\lambda}\otimes A_{\mathcal{N}_\lambda}. \tag{14}$$

Proof. Since $U(g)$ is a finite-dimensional representation of a compact group, it follows that it decomposes into a direct sum of irreducible representations:

$$\mathcal{H} \simeq \bigoplus_\lambda \bigoplus_{i_\lambda} \mathcal{H}_{i_\lambda}, \tag{15}$$

Applying Lemma 1 to each subspace $\mathcal{H}_\lambda := \bigoplus_{i_\lambda}\mathcal{H}_{i_\lambda}$ gives:

$$\mathcal{H} \simeq \bigoplus_\lambda \mathcal{M}_\lambda\otimes\mathcal{N}_\lambda, \tag{16}$$

$$U(g) \simeq \bigoplus_\lambda U_\lambda(g)\otimes \mathbb{I}_{\mathcal{N}_\lambda}. \tag{17}$$

An operator $A : \bigoplus \mathcal{H}_\lambda \to \mathcal{H}_\lambda$ can be decomposed as:

$$A = \sum_{\lambda,\lambda'}\sum_{i,j}\Pi_{\mathcal{H}_{\lambda'}^j} A \Pi_{\mathcal{H}_\lambda^i} = \sum_{\lambda,\lambda',i,j} A_{\lambda,\lambda',i,j}, \tag{18}$$

where each $A_{\lambda,\lambda',i,j}$ is an operator from $\mathcal{H}_\lambda^i$ to $\mathcal{H}_{\lambda'}^j$.

Invariance $[A, U(g)] = 0$ implies $A_{\lambda,\lambda',i,j}U_{\lambda'}^j(g) = U_\lambda^i(g)A_{\lambda,\lambda',i,j}$. For $\lambda \neq \lambda'$, Schur's Lemma entails that $A_{\lambda,\lambda',i,j} = 0$. Hence $A = \sum_{\lambda,i,j} A_{\lambda,\lambda,i,j}$ is block-diagonal in the $\mathcal{H}_\lambda$, and so $A_\lambda := \sum_{ij} A_{\lambda,\lambda,i,j}$ satisfies $[A_\lambda, U_\lambda(g)\otimes\mathbb{I}_{\mathcal{N}_\lambda}] = 0$. Using Lemma 3 on each $\mathcal{H}_\lambda \simeq \mathcal{M}_\lambda\otimes\mathcal{N}_\lambda$ gives:

$$A = \bigoplus_\lambda \mathbb{I}_{\mathcal{M}_\lambda}\otimes A_{\mathcal{N}_\lambda}. \tag{19}$$

This proves the main result of this section. □

SUPPLEMENTARY NOTE 2 – DESCRIPTIONS OF PARASTATISTICS

A. Examples and classification

We first illustrate a Bosonic, Fermionic and paraparticle representation in first quantization for the case of

$N = 3$ particles, as has been discussed in [4]. The action of standard permutations in this context is given by

$$U(\pi)|x_1, \ldots, x_N\rangle = |x_{\pi^{-1}(1)}, \ldots, x_{\pi^{-1}(N)}\rangle, \tag{20}$$

which permutes the tensor factors associated to Hilbert spaces describing single particles.

Example 1. *For simplicity, let us only consider states of the form $|x_1, x_2, x_3\rangle$ where x_1, x_2 and x_3 are all distinct and orthonormal. First, note that the states*

$$|b\rangle = \frac{1}{|S_3|} \sum_{\pi \in S_3} U(\pi)|x_1, x_2, x_3\rangle, \tag{21}$$

$$|f\rangle = \frac{1}{|S_3|} \sum_{\pi \in S_3} \mathrm{sgn}(\pi) U(\pi)|x_1, x_2, x_3\rangle \tag{22}$$

fulfill $U(\pi)|b\rangle = |b\rangle$ and $U(\pi)|f\rangle = \mathrm{sgn}(\pi)|f\rangle$ for all $\pi \in S_3$. Hence, they are contained in the symmetric (Bosonic) and antisymmetric (Fermionic) subspaces of $\mathcal{H}$, on which $U(\pi)$ acts as the trivial and the sign representation, respectively.

Now, for $\omega = e^{i\frac{2\pi}{3}}$, let us also consider the orthonormal states

$$|\psi_1\rangle = \frac{1}{\sqrt{3}}(|x_1, x_2, x_3\rangle + \omega|x_2, x_3, x_1\rangle + \bar{\omega}|x_3, x_1, x_2\rangle), \tag{23}$$

$$|\psi_2\rangle = \frac{1}{\sqrt{3}}(|x_2, x_1, x_3\rangle + \omega|x_3, x_2, x_1\rangle + \bar{\omega}|x_1, x_3, x_2\rangle). \tag{24}$$

For the permutation $\tau = (12)$ exchanging positions 1 and 2, we have $U(\tau)|\psi_1\rangle = |\psi_2\rangle$, while the cyclic permutation $\sigma = (123)$ gives $U(\sigma)|\psi_1\rangle = \omega|\psi_1\rangle$ and $U(\sigma)|\psi_2\rangle = \bar{\omega}|\psi_2\rangle$. Therefore, since every $\pi \in S_3$ is generated by the elements τ and σ, we find that $\mathcal{H}_{\mathrm{Para}} := \mathrm{span}\{|\psi_1\rangle, |\psi_2\rangle\}$ is a two-dimensional invariant subspace under $U(\pi)$, which acts as an irrep $U_{\mathrm{Para}}(\pi)$ on $\mathcal{H}_{\mathrm{Para}}$. This is the simplest example of a parastatistics sector. The corresponding Young diagram is given by

$\lambda_{\mathrm{Para}} =$ 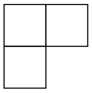 . (25)

Moreover, we observe that $U(\pi)$ acts as the regular representation $U_r(\pi)$ on the invariant subspace $\mathcal{H}_r := \mathrm{span}\{U(\pi)|x_1, x_2, x_3\rangle | \pi \in S_3\}$, where $|x_1, x_2, x_3\rangle$ is a fixed initial state, which contains the states $|b\rangle, |f\rangle, |\psi_1\rangle$ and $|\psi_2\rangle$ described there. Therefore, we must have

$$U_r(\pi) = 1 \oplus \mathrm{sgn}(\pi) \oplus U_{\mathrm{Para}}(\pi) \otimes \mathbb{I}_2, \tag{26}$$

and thus the orthogonal decomposition

$$\mathcal{H}_r = \mathbb{C}|b\rangle \oplus \mathbb{C}|f\rangle \oplus 2\mathcal{H}_{\mathrm{Para}}, \tag{27}$$

as $1, \mathrm{sgn}(\pi)$ and $U_{\mathrm{Para}}(\pi)$ are the only irreps of S_3. Furthermore, this tells us that $U_{\mathrm{Para}}(\pi)$ must appear twice. Indeed, a second instance of $\mathcal{H}_{\mathrm{Para}}$ is spanned by the orthonormal pair

$$|\phi_1\rangle = \frac{1}{\sqrt{3}}(|x_2, x_1, x_3\rangle + \bar{\omega}|x_3, x_2, x_1\rangle + \omega|x_1, x_3, x_2\rangle), \tag{28}$$

$$|\phi_2\rangle = \frac{1}{\sqrt{3}}(|x_1, x_2, x_3\rangle + \bar{\omega}|x_2, x_3, x_1\rangle + \omega|x_3, x_1, x_2\rangle), \tag{29}$$

for which we also find $\langle\psi_i|\phi_j\rangle = 0$, due to $1 + \omega + \bar{\omega} = 0$.

Moreover, given $U(\tau)|\psi_1\rangle = |\psi_2\rangle$ and $U(\tau)|\psi_2\rangle = |\psi_1\rangle$ for $\tau = (12)$, the states are related by a permutation and cannot be distinguished due to our postulate of permutation-invariance. Hence, they lead to the same physical predictions as the mixed state $\rho_\psi = \frac{1}{2}(|\psi_1\rangle\langle\psi_1| + |\psi_2\rangle\langle\psi_2|)$. This state is invariant, and is hence a valid description of preparation procedures according to permutation-invariance on the level of states. This is true for the pair $|\phi_1\rangle, |\phi_2\rangle$ as well, leading to ρ_ϕ there. However, although each pair cannot be distinguished, it is possible to distinguish the two pairs from each other, since ρ_ψ and ρ_ϕ are both invariant and orthogonal to one another.

Any collection of N Bosons (Fermions) is described by states contained in irreducible subspaces that correspond to the Young diagram of a single row (column) of N boxes. Moreover, let us consider a collection of $N+1$ Bosons (Fermions), N of which are localized in a lab and one very far away, such that it cannot be accessed by an observer in the lab. It is well-known that the representation obtained by restricting a representation of the group S_{N+1} with Young diagram λ to the subgroup S_N is the direct sum of all representations with Young diagram λ' obtained by removing a square from λ [3]. Hence, given the corresponding Young diagram of a single row (column) of $N+1$ boxes, the only valid Young diagram obtained by removing one box is the single row (column) of N boxes. Therefore, it is clear that the N particles in the lab must behave as Bosons (Fermions) as well. In the same way, by considering the para-sector of three particles as in Example 1 above, we can imagine a scenario where we only have access to two of the particles, and no access whatsoever to the third particle (this includes not having access to the outcomes of potential measurements that some other observer might have performed on the third particle). Since the mere existence of the third particle far away should not influence the measurement outcome statistics in the lab, the accessible pair should therefore either behave as a pair of Bosons or Fermions.

This is indeed shown in [5] and generalized in their classification of paraparticles [6]: parabosons (parafermions) of order $p \in \mathbb{N}$ correspond to the family of Young diagrams with a maximum number of p rows (columns). Restricting to a subset of the particles corresponds to removing boxes from the Young diagrams, and this preserves the particle type if it is defined in this way. Note that parafermions as used here should not be confused

with the notion of parafermions in the context of non-Abelian anyons [7, 8].

B. Parastatistics in second quantization

It is second quantization in which parastatistics was first conceived of by Green [9]. There, Bosons (Fermions) are described by a Fock space $\mathcal{F}$ generated by creation operators $a_k^\dagger$ that fulfill the usual (anti)commutation relations, where k denote modes such as momentum or position. However, in order to preserve the equations of motion, one can have more general, trilinear (anti)commutation relations [9, 10]:

$$[a_{k_1}, [a_{k_2}^\dagger, a_{k_3}]_\pm]_- = 2\delta_{k_1 k_2} a_{k_3}, \tag{30}$$
$$[a_{k_1}, [a_{k_2}^\dagger, a_{k_3}^\dagger]_\pm]_- = 2\delta_{k_1 k_2} a_{k_3}^\dagger \pm 2\delta_{k_1 k_3} a_{k_2}^\dagger, \tag{31}$$
$$[a_{k_1}, [a_{k_2}, a_{k_3}]_\pm]_- = 0, \tag{32}$$

with $[X, Y]_\pm := XY \pm YX$ denoting anticommutators and commutators, respectively. These relations led to a whole new class of fields that were eventually named parabosons (parafermions), corresponding to the choice of anticommutators (commutators) above. Green's ansatz of solutions for the trilinear equations involves writing the parabosonic (parafermionic) operators as sums of p distinct Bosonic (Fermionic) creation and annihilation operators:

$$a_k^{(\dagger)} = \sum_{\alpha=1}^{p} c_{\alpha,k}^{(\dagger)} \tag{33}$$

with

$$[c_{\alpha,k_1}^\dagger, c_{\alpha,k_2}]_\mp = \delta_{k_1,k_2}, \qquad [c_{\alpha,k_1}, c_{\alpha,k_2}]_\mp = 0$$
$$[c_{\alpha,k_1}^\dagger, c_{\beta,k_2}]_\pm = 0, \qquad [c_{\alpha,k_1}, c_{\beta,k_2}]_\pm = 0, \qquad \alpha \neq \beta \tag{34}$$

Parabosonic (parafermionic) operators $a_k^{(\dagger)}$ of order p are then identified with sums of p Bosonic (Fermionic) operators $c_{\alpha,k}^{(\dagger)}$ which commute (anticommute) for $\alpha = \beta$ and anticommute (commute) for $\alpha \neq \beta$. Moreover, it can be shown [10] that they exhaust all possible solutions compatible with the trilinear relations. In addition to the compatibility with the equations of motion, the trilinear relations can also be derived by requiring invariance of the number operator under unitary changes of basis [11].

The N-particle subspace of the Fock space is spanned by vectors

$$|k_1, ..., k_N\rangle_\mathcal{F} := a_{k_1}^\dagger ... a_{k_N}^\dagger |0\rangle_\mathcal{F}, \tag{35}$$

where $|0\rangle_\mathcal{F}$ is the vacuum state. By fixing the entries and order of such a tuple $K := (k_1, ..., k_n)$, one can define a representation $\bar{U}_K(\pi)$ of S_N on the subspace $\mathcal{H}_K := \text{span}\{|k_{\pi(1)}, ..., k_{\pi(N)}\rangle_\mathcal{F} | \pi \in S_N\}$, which transforms creation operators $a_{k_i}^\dagger$ into $\bar{U}_K(\pi) a_{k_i}^\dagger \bar{U}_K(\pi)^\dagger = a_{k_{\pi(i)}}^\dagger$. It can then be shown [12] that this representation decomposes in the following way: For parabosons (parafermions) of order p, on each of these subspaces, the representation contains exactly once each irrep whose Young diagram has no more than p rows (columns). Thus, a connection between the parafields of second quantization and the family of Young diagrams associated to each paraparticle type in first quantization is established. Defining $\bar{U}_K(\pi)$ for every ordered tuple K leads to a consistent definition of standard permutations $\bar{U}(\pi)$ in second quantization, known as *particle permutations* (for more details, see Subsection F).

Example 2. *Following the Volkov model from [13], we illustrate the case for the simplest parafermion of order 2. There, a product of three creation operators fulfills the relation* $a_i^\dagger a_j^\dagger a_k^\dagger = -a_k^\dagger a_j^\dagger a_i^\dagger$ *for* $i, j, k \in \{1, 2, 3\}$*. This relation halves the dimension of the space of states created from the vacuum* $|0\rangle_\mathcal{F}$ *down to 3, and the correspondence described above tells us that it will consist of irrep spaces associated to Young diagrams with a maximum of 2 columns. Indeed, on the one hand we find the state*

$$|f\rangle_\mathcal{F} = \frac{1}{\sqrt{3}} \left(a_1^\dagger a_2^\dagger a_3^\dagger + a_2^\dagger a_3^\dagger a_1^\dagger + a_3^\dagger a_1^\dagger a_2^\dagger \right) |0\rangle_\mathcal{F}, \tag{36}$$

which exhibits usual Fermionic statistics, i.e. it carries the one-dimensional sign representation under particle permutations. On the other hand we find the two-dimensional irrep space spanned by the states

$$|\psi_1\rangle_\mathcal{F} = \frac{1}{\sqrt{3}} \left(a_1^\dagger a_2^\dagger a_3^\dagger + \bar{\omega} a_2^\dagger a_3^\dagger a_1^\dagger + \omega a_3^\dagger a_1^\dagger a_2^\dagger \right) |0\rangle_\mathcal{F}, \tag{37}$$
$$|\psi_2\rangle_\mathcal{F} = \frac{1}{\sqrt{3}} \left(a_2^\dagger a_1^\dagger a_3^\dagger + \bar{\omega} a_1^\dagger a_3^\dagger a_2^\dagger + \omega a_3^\dagger a_2^\dagger a_1^\dagger \right) |0\rangle_\mathcal{F}$$
$$= -\frac{1}{\sqrt{3}} \left(a_3^\dagger a_1^\dagger a_2^\dagger + \bar{\omega} a_2^\dagger a_3^\dagger a_1^\dagger + \omega a_1^\dagger a_2^\dagger a_3^\dagger \right) |0\rangle_\mathcal{F}, \tag{38}$$

with $\omega = e^{i\frac{2\pi}{3}}$*. As in first quantization, for the permutation* $\tau = (12)$ *we have* $\bar{U}(\tau)|\psi_1\rangle_\mathcal{F} = |\psi_2\rangle_\mathcal{F}$*, while the cyclic permutation* $\sigma = (123)$ *gives* $\bar{U}(\sigma)|\psi_1\rangle_\mathcal{F} = \omega|\psi_1\rangle_\mathcal{F}$ *and* $\bar{U}(\sigma)|\psi_2\rangle_\mathcal{F} = \bar{\omega}|\psi_2\rangle_\mathcal{F}$*. A permutation-invariant and therefore physically allowed state is given by*

$$\rho_S = \frac{1}{2} \left(|\psi_1\rangle\langle\psi_1|_\mathcal{F} + |\psi_2\rangle\langle\psi_2|_\mathcal{F} \right). \tag{39}$$

It might seem at first sight that the postulate of permutation-invariance renders parastatistics trivial: in the previous example, it enforced a uniform mixture on the subspace spanned by $|\psi_1\rangle$ and $|\psi_2\rangle$, and one might conjecture that this "averages out" all non-trivial consequences of parastatistics. However, in Example 3 below, we show that this is not the case, and that interesting permutation-invariant physical behavior can be obtained, even if we have a single particle in each mode. Allowing situations with more than one particle per mode shows this in an even simpler way. Consider three parafermions in two modes. There are two orthogonal

particle-permutation-invariant states

$$|\psi_\pm\rangle := (a_1^\dagger a_1^\dagger a_2^\dagger \pm a_2^\dagger a_2^\dagger a_1^\dagger)|0\rangle, \tag{40}$$

and the other possible permutation-invariant combinations are:

$$(a_1^\dagger a_2^\dagger a_1^\dagger \pm a_2^\dagger \hat{a}_1^\dagger a_2^\dagger)|0\rangle = 0 \tag{41}$$

$$(a_1^\dagger a_2^\dagger a_2^\dagger \pm a_2^\dagger a_1^\dagger a_1^\dagger)|0\rangle = \mp|\psi_\pm\rangle \tag{42}$$

Since it is possible to have two modes with three excitations, these particles are not Fermions. Moreover, since it is not possible to have three excitations in one mode, they also behave differently from Bosons.

We have just seen an example for parafermionic states in second quantization. For a given triple of distinct modes and under the postulate of (particle) permutation-invariance, there is one physically allowed state. However, one must note that the multiplicity of possible states arises from having other degrees of freedom available, i.e. more modes, on which we can condition. This is in no way different for a permutation-invariant state of a pair of Bosons and Fermions, when there are only two modes available. In the following example, we will introduce an additional spin degree of freedom and implement a conditional permutation to show that paraparticles are still detectable, as well as different from Bosons and Fermions despite imposing permutation-invariance.

Example 3. *Recall Example 2 involving three parafermions in three distinct modes, and consider an additional qubit with basis states* $\{|0\rangle, |1\rangle\}$. *This qubit may describe an arbitrary external degree of freedom, or it may (similarly as in the experimental proposal by Roos et al. [14]) describe internal degrees of freedom of the particles, for example* $|0\rangle = |\uparrow\uparrow\uparrow\rangle$ *if all three spins are up, or* $|1\rangle = |\downarrow\downarrow\downarrow\rangle$ *if all three spins are down. We start with the permutation-invariant initial product state*

$$\rho = \frac{1}{4}\left(|\psi_1\rangle\langle\psi_1|_{\mathcal{F}} + |\psi_2\rangle\langle\psi_2|_{\mathcal{F}}\right) \otimes (|0\rangle + |1\rangle)(\langle 0| + \langle 1|). \tag{43}$$

We will restrict our considerations to the subspace spanned by $|\psi_1\rangle$ *and* $|\psi_2\rangle$, *because* ρ *is fully supported on it. Similarly as in the Roos et al. [14] experiment, we perform a permutation that is coherently controlled by the qubit: if the qubit is in state* 0, *we do nothing; if it is in state* 1, *we do a cyclic permutation. However, whether we apply the cyclic permutation* $\sigma = (123)$ *or* σ^{-1} *shall also be controlled by whether the particles are in state* $|\psi_1\rangle$ *or* $|\psi_2\rangle$. *That is, we apply the unitary transformation*

$$\begin{aligned}\bar{V} &= \mathbb{I}\left(|\psi_1\rangle\langle\psi_1|_{\mathcal{F}} \otimes |0\rangle\langle 0| + |\psi_2\rangle\langle\psi_2|_{\mathcal{F}} \otimes |0\rangle\langle 0|\right) \\ &\quad + \bar{U}(\sigma)|\psi_1\rangle\langle\psi_1|_{\mathcal{F}} \otimes |1\rangle\langle 1| + \bar{U}(\sigma^{-1})|\psi_2\rangle\langle\psi_2|_{\mathcal{F}} \otimes |1\rangle\langle 1|.\end{aligned} \tag{44}$$

This is a conditional permutation, but it is not a quantum permutation in the sense of Definition 1 in the main text: the projectors $|\psi_i\rangle\langle\psi_i|_{\mathcal{F}} \otimes |1\rangle\langle 1|$ *do not commute with all permutations. However, they do commute with those permutations* $U(\pi)$ *where* π *is in the cyclic group* $\mathbb{Z}_3 \simeq \{\mathbb{I}, \sigma, \sigma^{-1}\}$. *Hence, we can regard it as a valid* $\mathbb{Z}_3$ *QRF transformation. It maps the initial state to* $\rho' := \bar{V}\rho\bar{V}^\dagger$, *which is*

$$\rho' = \frac{1}{4}\left(|\psi_1\rangle\langle\psi_1|_{\mathcal{F}} + |\psi_2\rangle\langle\psi_2|_{\mathcal{F}}\right) \otimes (|0\rangle + \omega|1\rangle)(\langle 0| + \bar{\omega}\langle 1|), \tag{45}$$

i.e. it induces a relative phase of $\omega = \exp\left(\frac{2\pi}{3}i\right)$ *on the control qubit. This is clearly impossible for Bosons and Fermions. To see that it has this effect, note that* $\bar{V}$ *can be simplified to*

$$\bar{V} = \left(|\psi_1\rangle\langle\psi_1|_{\mathcal{F}} + |\psi_2\rangle\langle\psi_2|_{\mathcal{F}}\right) \otimes \big(|0\rangle\langle 0| + \omega|1\rangle\langle 1|\big), \tag{46}$$

which also shows that it is indeed a permutation-invariant transformation, mapping every permutation-invariant state to a permutation-invariant state.

It may seem surprising that we should regard this as an allowed operation: after all, it performs an action depending on whether the internal degree of freedom is $|\psi_1\rangle$ *or* $|\psi_2\rangle$, *and permutation-invariance forbids the measurement of the corresponding projectors. However, these projectors are not used for measurement, but for coherent control which can be thought of as fundamentally erasing the corresponding outcome in the process. Hence, particle-permutation-invariant states of three parafermions, occupying three distinct modes, can induce relative phases of* $\omega = \exp\left(\frac{2\pi}{3}i\right)$ *via physically allowed conditional permutations which Bosons and Fermions cannot.*

Both Examples 2 and 3 can be understood in the same way for parabosons when the relation $a_i^\dagger a_j^\dagger a_k^\dagger = a_k^\dagger a_j^\dagger a_i^\dagger$ is fulfilled by the creation operators. Both relations are instances of trilinear relations that are more general than ordinary (anti)commutation relations, corresponding to parabosons (parafermions) of the lowest non-trivial order 2. In the case of parafermions, the order coincides with the maximum occupation number per mode: in contrast to ordinary Fermions, $(a_i^\dagger)^2 \neq 0$, but the relations clearly imply that $(a_i^\dagger)^3 = 0$. However, in the special arrangement that the operators have in the Fermionic state $|f\rangle_{\mathcal{F}}$ in Example 2, setting any two of them equal will lead to 0, as expected.

C. Example of emergent parastatistics

In this subsection, we give an example (following [15, Section 9]) that shows how parafermions can emerge from Bosons with additional degrees of freedom. In Subsection D, we will see that it demonstrates an intuitive reason for why and how complete invariance fails for such emergent systems.

Example 4. *We begin with three distinguishable isospin doublets $\mathcal{H}^{(3)} = (L^2(\mathbb{R}^3)\otimes\mathbb{C}^2)^{\otimes 3}$, which carry the following representation of S_3:*

$$U^{(3)}(\pi)\psi_{a_1,a_2,a_3}(q_1,q_2,q_3) = \psi_{a_{\pi(1)},a_{\pi(2)},a_{\pi(2)}}(q_{\pi(1)},q_{\pi(2)},q_{\pi(3)}). \tag{47}$$

We consider the subspace $\mathcal{H}^{(3)}_{\lambda_0}$ of Bosonic isospin doublets spanned by symmetrized wavefunctions:

$$\psi_{a_1,a_2,a_3}(q_1,q_2,q_3) = \psi_{a_{\pi(1)},a_{\pi(2)},a_{\pi(2)}}(q_{\pi(1)},q_{\pi(2)},q_{\pi(3)}). \tag{48}$$

Using Schur-Weyl for the action of S_3 given in Equation (47) (i.e. permuting both spatial and spin labels) we have that the space $\mathcal{H}^{(3)}$ decomposes as:

$$\mathcal{H}^{(3)} \simeq \bigoplus_\lambda V_\lambda \otimes W_\lambda \simeq \bigoplus_\lambda \mathcal{H}^{(3)}_\lambda, \tag{49}$$

where V_λ carries the irreducible representation of S_3 labeled by λ and W_λ a representation of $U(W_\lambda)$. Since W_λ is infinite-dimensional, one needs to choose the correct topology on W_λ to define $U(W_\lambda)$, as discussed in [15, footnote 7].

However we can further decompose $\mathcal{H}^{(3)}_{\lambda_0}$ by considering the fact that the spatial and spin components of $\mathcal{H}^{(3)}$ carry separate actions of S_3. We write $\mathcal{H}^{(3)} \simeq \mathcal{H}^{(3),\mathrm{spatial}} \otimes \mathcal{H}^{(3),\mathrm{spin}}$ where $\mathcal{H}^{(3),\mathrm{spatial}} \simeq L^2(\mathbb{R}^3)^{\otimes 3}$ and $\mathcal{H}^{(3),\mathrm{spin}} \simeq (\mathbb{C}^2)^{\otimes 3}$. $\mathcal{H}^{(3),\mathrm{spatial}}$ carries a representation $U^{(3),\mathrm{spatial}}(\pi)$ of S_3 (corresponding to permuting the spatial indices) and similarly $\mathcal{H}^{(3),\mathrm{spin}}$ carries a representation $U^{(3),\mathrm{spin}}(\pi)$ of S_3 (corresponding to permuting the spin indices).

The representation $U^{(3)}$ of S_3 given in Equation (47) corresponds to performing the same permutation π on both $\mathcal{H}^{(3),\mathrm{spatial}}$ and $\mathcal{H}^{(3),\mathrm{spin}}$, i.e. $U^{(3)}(\pi) = U^{(3),\mathrm{spatial}}(\pi)\otimes U^{(3),\mathrm{spin}}(\pi)$.

Using Schur-Weyl we can first decompose the spaces $\mathcal{H}^{(3),\mathrm{spatial}}$ and $\mathcal{H}^{(3),\mathrm{spin}}$ under their respective S_3 actions:

$$\begin{aligned}\mathcal{H}^{(3),\mathrm{spatial}} = L^2(\mathbb{R}^3)^{\otimes 3} &\simeq \bigoplus_\lambda V^{\mathrm{spatial}}_\lambda \otimes W^{\mathrm{spatial}}_\lambda \\ &\simeq \bigoplus_\lambda \mathcal{H}^{(3),\mathrm{spatial}}_\lambda, \end{aligned}\tag{50}$$

$$\begin{aligned}\mathcal{H}^{(3),\mathrm{spin}} = (\mathbb{C}^2)^{\otimes 3} &\simeq \bigoplus_\lambda V^{\mathrm{spin}}_\lambda \otimes W^{\mathrm{spin}}_\lambda \\ &\simeq \bigoplus_\lambda \mathcal{H}^{(3),\mathrm{spin}}_\lambda, \end{aligned}\tag{51}$$

where $V^{\mathrm{spatial}}_\lambda, V^{\mathrm{spin}}_\lambda$ carry the irreducible representation of S_3 labeled by λ, $W^{\mathrm{spatial}}_\lambda$ a representation of $U(W^{\mathrm{spatial}}_\lambda)$ and $W^{\mathrm{spin}}_\lambda$ an irreducible representation of SU(2).

Clearly for λ_0 the Bosonic representation, any state in $\mathcal{H}^{(3),\mathrm{spatial}}_{\lambda_0} \otimes \mathcal{H}^{(3),\mathrm{spin}}_{\lambda_0}$ transforms trivially under permutations and is therefore in $\mathcal{H}^{(3)}_{\lambda_0}$. This is just the statement that the symmetric subspace $\mathcal{H}^{(3)}_{\lambda_0}$ of $\mathcal{H}^{(3)} \simeq \mathcal{H}^{(3),\mathrm{spatial}} \otimes \mathcal{H}^{(3),\mathrm{spin}}$ contains the tensor product of the symmetric subspaces of the factors, namely $\mathcal{H}^{(3),\mathrm{spatial}}_{\lambda_0} \otimes \mathcal{H}^{(3),\mathrm{spin}}_{\lambda_0} \subset \mathcal{H}^{(3)}_{\lambda_0}$. However, as is well-known, the symmetric subspace of a composite system also contains the tensor product of the antisymmetric subspaces of the factors: $\mathcal{H}^{(3),\mathrm{spatial}}_{\lambda_1} \otimes \mathcal{H}^{(3),\mathrm{spin}}_{\lambda_1} \subset \mathcal{H}^{(3)}_{\lambda_0}$ where λ_1 labels the antisymmetric subspace. Similarly for λ_2 the $(2,1)$ representation of S_3 there will be elements in $\mathcal{H}^{(3),\mathrm{spatial}}_{\lambda_2} \otimes \mathcal{H}^{(3),\mathrm{spin}}_{\lambda_2}$ which are invariant under $U^{(3),\mathrm{spatial}}_{\lambda_2}(\pi) \otimes U^{(3),\mathrm{spin}}_{\lambda_2}(\pi)$, i.e. which lie in $\mathcal{H}^{(3)}_{\lambda_0}$

For $\lambda_2 = (2,1)$ we define a permutation-invariant state in $V^{\mathrm{spatial}}_{\lambda_2} \otimes V^{\mathrm{spin}}_{\lambda_2}$, where $V^{\mathrm{spatial}}_{\lambda_2} \simeq V^{\mathrm{spin}}_{\lambda_2} \simeq \mathbb{C}^2$ both transform under the $(2,1)$ irrep of S_3. From the well known fact that the singlet state is invariant under $U \otimes U$ we can define the following Bosonic state in $\mathcal{H}^{(3)}_{\lambda_0} \subset \mathcal{H}^{(3)}$:

$$|\Psi\rangle = \left(\frac{1}{\sqrt{2}}(|e_0\rangle_{V^{\mathrm{spatial}}_{\lambda_2}} \otimes |e_1\rangle_{V^{\mathrm{spin}}_{\lambda_2}} - |e_1\rangle_{V^{\mathrm{spatial}}_{\lambda_2}} \otimes |e_0\rangle_{V^{\mathrm{spin}}_{\lambda_2}})\right) \otimes |\psi\rangle_{W^{\mathrm{spatial}}_{\lambda_2}, W^{\mathrm{spin}}_{\lambda_2}}, \tag{52}$$

where $\{|e_i\rangle_{V^{\mathrm{spatial}}_{\lambda_2}}\}_{i=0,1}$ is a basis for $V^{\mathrm{spatial}}_{\lambda_2}$ (and similarly for $V^{\mathrm{spin}}_{\lambda_2}$) and $|\psi\rangle_{W^{\mathrm{spatial}}_{\lambda_2}, W^{\mathrm{spin}}_{\lambda_2}}$ an arbitrary state in $W^{\mathrm{spatial}}_{\lambda_2}, W^{\mathrm{spin}}_{\lambda_2}$. This is clearly invariant since a simultaneous permutation $U^{(3)}(\pi) = U^{(3),\mathrm{spatial}}(\pi) \otimes U^{(3),\mathrm{spin}}(\pi)$ of the spatial and spin indices acts on $\mathcal{H}^{(3),\mathrm{spatial}}_{\lambda_2} \otimes \mathcal{H}^{(3),\mathrm{spin}}_{\lambda_2}$ as $U_{V^{\mathrm{spatial}}_{\lambda_2}}(\pi) \otimes U_{V^{\mathrm{spin}}_{\lambda_2}}(\pi) \otimes \mathbb{I}_{W^{\mathrm{spatial}}_{\lambda_2}} \otimes \mathbb{I}_{W^{\mathrm{spin}}_{\lambda_2}}$ and the singlet state over the first two factors is invariant under any $U \otimes U$.

Let us ignore the isospin degrees of freedom and trace out $V^{\mathrm{spin}}_\lambda \otimes W^{\mathrm{spin}}_\lambda$:

$$\rho_{\mathrm{para}} := \mathrm{Tr}_{V^{\mathrm{spin}}_{\lambda_2} W^{\mathrm{spin}}_{\lambda_2}} |\Psi\rangle\langle\Psi| = \frac{1}{2}\mathbf{1}_{V^{\mathrm{spatial}}_{\lambda_2}} \otimes \rho_{W^{\mathrm{spatial}}_{\lambda_2}}, \tag{53}$$

where $\rho_{W^{\mathrm{spatial}}_{\lambda_2}} = \mathrm{Tr}_{W_{\lambda^{\mathrm{spin}}_2}}(|\psi\rangle\langle\psi|_{W^{\mathrm{spatial}}_{\lambda_2}, W^{\mathrm{spin}}_{\lambda_2}})$. This is indeed a parafermionic state of three particles with just spatial degrees of freedom, since $\sum_i |e_i\rangle\langle e_i|_{V^{\mathrm{spatial}}_\lambda}$ is the identity on the space $V^{\mathrm{spatial}}_\lambda$ transforming under the $(2,1)$ irrep of S_3 and $V^{\mathrm{spatial}}_\lambda$ transforms trivially under S_3.

SUPPLEMENTARY NOTE 3 – COMPLETE INVARIANCE

D. Failure of complete invariance for emergent paraparticles

The effective paraparticles in Example 4 above are permutation-invariant. But does it make sense to require complete invariance? The answer is in the negative, as we now show.

Given an agent Alice using the effective paraparticles of Equation (53), an adversary Eve without restriction would have access to the purification given in Equation (52).

Alice can implement permutations only on the spatial components, and not the spin (since by construction of the effective paraparticles she does not have access to them). Her state ρ_{para} is indeed invariant under $U_{V_{\lambda_2}^{\text{spatial}}}(\sigma)$ for all $\sigma \in S_3$.

The purification $|\Psi\rangle$ which Eve has access to is not invariant under $U_{V_{\lambda_2}^{\text{spatial}}}(\sigma) \otimes \mathbb{I}_{W_{\lambda_2}^{\text{spatial}}} \otimes \mathbb{I}_{V_{\lambda_2}^{\text{spin}}}(\sigma) \otimes \mathbb{I}_{W_{\lambda_2}^{\text{spin}}}$ and therefore does not obey complete invariance. Remember that the requirement of being a Bosonic state meant invariance under a different condition, namely $U_{V_{\lambda_2}^{\text{spatial}}}(\sigma) \otimes \mathbb{I}_{W_{\lambda_2}^{\text{spatial}}} \otimes U_{V_{\lambda_2}^{\text{spin}}}(\sigma) \otimes \mathbb{I}_{W_{\lambda_2}^{\text{spin}}}$.

We can see that the purification is not invariant under $U_{V_{\lambda_2}^{\text{spatial}}}(\sigma)$ from the following facts about the $(2,1)$ irrep of S_3, namely that for $\sigma = (123)$:

$$U(\sigma)|e_0\rangle = \omega|e_0\rangle, \tag{54}$$
$$U(\sigma)|e_1\rangle = \bar{\omega}|e_1\rangle, \tag{55}$$

where $\omega = e^{\frac{2\pi i}{3}}$. Therefore:

$$\begin{aligned} &U_{V_{\lambda_2}^{\text{spatial}}}(\sigma) \otimes \mathbb{I}_{W_{\lambda_2}^{\text{spatial}}} \otimes \mathbb{I}_{V_{\lambda_2}^{\text{spin}}}(\sigma) \otimes \mathbb{I}_{W_{\lambda_2}^{\text{spin}}}|\Psi\rangle \\ =&\frac{1}{\sqrt{2}}\left(\omega|e_0\rangle_{V_{\lambda_2}^{\text{spatial}}} \otimes |e_1\rangle_{V_{\lambda_2}^{\text{spin}}} - \bar{\omega}|e_1\rangle_{V_{\lambda_2}^{\text{spatial}}} \otimes |e_0\rangle_{V_{\lambda_2}^{\text{spin}}}\right) \\ &\otimes |\psi\rangle_{W_{\lambda_2}^{\text{spatial}}, W_{\lambda_2^{\text{spin}}}} \end{aligned} \tag{56}$$

$$\begin{aligned} \simeq&\frac{1}{\sqrt{2}}\left(|e_0\rangle_{V_{\lambda_2}^{\text{spatial}}} \otimes |e_1\rangle_{V_{\lambda_2}^{\text{spin}}} - \omega|e_1\rangle_{V_{\lambda_2}^{\text{spatial}}} \otimes |e_0\rangle_{V_{\lambda_2}^{\text{spin}}}\right) \\ &\otimes |\psi\rangle_{W_{\lambda_2}^{\text{spatial}}, W_{\lambda_2^{\text{spin}}}} \not\simeq |\Psi\rangle \end{aligned} \tag{57}$$

where $\simeq$ denotes equivalence up to global phase.

The above example shows that given an effective system of paraparticles emerging from an underlying Bosonic system, the assumption of complete invariance fails exactly because the full underlying Bosonic state is not invariant under permutations acting solely on the parastatistical subsystem.

E. Complete invariance for compact groups

In this section, we show that our results on complete invariance do not only hold for the permutation group, but more generally for unitary representations of compact groups G on separable Hilbert spaces. We will always assume that G is, as a topological space, a Hausdorff space.

Since Schur's Lemma applies to unitary representations of arbitrary locally compact Hausdorff groups [1, Proposition 5.8], and since finite-dimensional representations of such groups are completely reducible, Lemma 4 and therefore all the results in this section also apply to *finite-dimensional* representations of such more general groups.

Definition 1 (Extension of a state)**.** *An extension of a state ρ_S is a state ρ_{SA} such that $\text{Tr}_A(\rho_{SA}) = \rho_S$.*

Definition 2 (Invariant (weakly symmetric) state)**.** *An invariant (or weakly symmetric) state ρ_S for a given symmetry $U(g)$ is such that $\rho_S = U(g)\rho_S U^\dagger(g)$ for all $g \in G$.*

By Lemma 3 (where $\mathcal{H}_A$ is trivial) it follows that a weakly invariant state is of the form

$$\rho = \bigoplus p_\lambda \frac{\mathbb{I}_{\mathcal{M}_\lambda}}{d_\lambda} \otimes \rho_{\mathcal{N}_\lambda}. \tag{58}$$

Definition 3 (Strongly symmetric state)**.** *A strongly symmetric state ρ for a symmetry $U(g) \simeq \bigoplus_\lambda U_\lambda(g) \otimes \mathbb{I}_{\mathcal{N}_\lambda}$ has full support on a one-dimensional irreducible representation of G:*

$$\rho = |e_\lambda\rangle\langle e_\lambda|_{\mathcal{M}_\lambda} \otimes \rho_{\mathcal{N}_\lambda}, \tag{59}$$

where $\mathcal{M}_\lambda$ carries a one-dimensional representation of G: $U_\lambda(g) = e^{i\theta_\lambda(g)}$.

Definition 4 (Invariant extension)**.** *Given a state ρ_S an extension ρ_{SA} is invariant iff*

$$(U_S(g) \otimes \mathbb{I}_A)\rho_{SA}(U_S^\dagger(g) \otimes \mathbb{I}_A) = \rho_{SA}. \tag{60}$$

It is immediate that the existence of an invariant extension ρ_{SA} for a state ρ_S implies that ρ_S is invariant:

$$(U_S(g) \otimes \mathbb{I}_A)\rho_{SA}(U_S^\dagger(g) \otimes \mathbb{I}_A) = \rho_{SA} \tag{61}$$
$$\implies U_S(g)\rho_S U_S^\dagger(g) = \rho_S. \tag{62}$$

Lemma 5 (Invariant purification)**.** *If a state $\rho_S = U_S(g)\rho_S U_S^\dagger(g)$ has an invariant purification $|\psi\rangle_{SA}$ then ρ_S has support on a single one-dimensional irreducible representation λ of G:*

$$\rho_S = |e_\lambda\rangle\langle e_\lambda|_{\mathcal{M}_\lambda} \otimes \rho_{\mathcal{N}_\lambda^S}. \tag{63}$$

Proof. A generic invariant state ρ_S has the form

$$\rho_S = \bigoplus_\lambda p_\lambda \frac{\mathbb{I}_{\mathcal{M}_\lambda^S}}{d_\lambda} \otimes \rho_{\mathcal{N}_\lambda^S}. \tag{64}$$

The invariant operators on $\mathcal{H}_S \otimes \mathcal{H}_A$ under $U_S(g) \otimes \mathbb{I}_A$ are of the form

$$\bigoplus_\lambda \mathbb{I}_{\mathcal{M}_\lambda^S} \otimes A_{\mathcal{N}_\lambda^S, \mathcal{H}_A}. \tag{65}$$

A generic invariant extension ρ_{SA} of ρ_S therefore has the form

$$\rho_{SA} = \bigoplus_\lambda p_\lambda \frac{\mathbb{I}_{\mathcal{M}_\lambda^S}}{d_{\mathcal{M}_\lambda^S}} \otimes \rho_{\mathcal{N}_\lambda^S,\mathcal{H}_A}, \tag{66}$$

where

$$\mathrm{Tr}_{\mathcal{H}_A}(\rho_{\mathcal{N}_\lambda^S,\mathcal{H}_A}) = \rho_{\mathcal{N}_\lambda^S}. \tag{67}$$

Purity of ρ_{SA} implies that

$$\rho_{SA} = |e_\lambda\rangle\langle e_\lambda|_{\mathcal{M}_\lambda^S} \otimes |\psi_\lambda\rangle\langle\psi_\lambda|_{\mathcal{N}_\lambda^S,\mathcal{H}_A}, \tag{68}$$

where λ is a one-dimensional representation: $\frac{\mathbb{I}_{\mathcal{M}_\lambda^S}}{d_{\mathcal{M}_\lambda^S}} = |e_\lambda\rangle\langle e_\lambda|_{\mathcal{M}_\lambda^S}$.

This implies that $\rho_S = |e_\lambda\rangle\langle e_\lambda|_{\mathcal{M}_\lambda} \otimes \rho_{\mathcal{N}_\lambda^S}$, where λ is a one-dimensional irreducible representation. □

Lemma 6. *The following conditions are all equivalent, and can be used to define what it means that a quantum state ρ_S is* ***completely invariant*** *under a representation $U(g)$, $g \in G$:*

(i) Equation (60) *holds for some purification ρ_{SA} of ρ_S;*

(ii) Equation (60) *holds for all purifications ρ_{SA} of ρ_S;*

(iii) Equation (60) *holds for all extensions ρ_{SA} of ρ_S.*

Moreover, all three are equivalent to

(iv) ρ_S has full support on a single one-dimensional irreducible representation λ of G.

Proof. Clearly, $(iii) \Rightarrow (ii) \Rightarrow (i)$, and by Lemma 5 (i) implies (iv), i.e. that $\rho_S = |e_\lambda\rangle\langle e_\lambda|_{\mathcal{M}_\lambda} \otimes \rho_{\mathcal{N}_\lambda^S}$, where λ is a one-dimensional irreducible representation.

Let us finally show that (iv) $\Rightarrow$ (iii). An arbitrary extension ρ_{SA} of ρ_S is therefore of the form

$$\rho_{SA} = |e_\lambda\rangle\langle e_\lambda|_{\mathcal{M}_\lambda} \otimes \rho_{\mathcal{N}_\lambda^S,\mathcal{H}_A}, \tag{69}$$

where $\mathrm{Tr}_{\mathcal{H}_A}(\rho_{\mathcal{N}_\lambda^S,\mathcal{H}_A}) = \rho_{\mathcal{N}_\lambda^S}$. The state ρ_{SA} is invariant under $U_S(g) \otimes \mathbb{I}_A$ by construction, since the action of $U_S(g) \otimes \mathbb{I}_A$ on $\mathcal{M}_\lambda^S \otimes \mathcal{N}_\lambda^S \otimes \mathcal{H}_A$ is

$$U_\lambda(g) \otimes \mathbb{I}_{\mathcal{N}_\lambda^S} \otimes \mathbb{I}_{\mathcal{H}_A} = e^{i\theta(g)} |e_\lambda\rangle\langle e_\lambda|_{\mathcal{M}_\lambda} \otimes \mathbb{I}_{\mathcal{N}_\lambda^S} \otimes \mathbb{I}_{\mathcal{H}_A}, \tag{70}$$

since $\mathcal{M}_\lambda^S$ is one-dimensional. □

This implies the following theorem:

Theorem 1. *A quantum state ρ_S is completely invariant under some compact group G with representation $U_S(g)$, $g \in G$, if and only if it is fully supported on a subspace transforming under a one-dimensional representation of G.*

Note that an invariant state ρ_S with support on multiple irreducible subspaces, including subspaces with irreducible representations of dimension strictly greater than 1, may have *some* extensions ρ_{SA} which are invariant under the conjugate action of $U_S(g) \otimes \mathbb{I}_A$. ButLemma 6 tells us that this extension cannot be pure, and moreover that this cannot be the case for all extensions.

Since complete invariance entails that a state ρ_S must have full support on a subspace transforming according to a one-dimensional representation of G, we may wonder if standard invariance of a state ρ_S can be motivated by appealing to an environment also. The following theorem shows that this is indeed the case.

Theorem 2. *A quantum state ρ_S is (weakly) invariant under some compact group G, i.e. $\rho_S = U(g)\rho_S U_S^\dagger(g)$, if and only if there exists a purification $|\psi\rangle_{SA}$ of ρ_S on some ancilla $\mathcal{H}_A$ with some representation $U_A(g)$ such that $\big(U_S(g) \otimes U_A(g)\big)|\psi\rangle\langle\psi|_{SA}\big(U_S(g) \otimes U_A(g)\big)^\dagger = |\psi\rangle\langle\psi|_{SA}$.*

Proof. We first prove the $\Leftarrow$ direction. Consider a purification $|\psi\rangle_{SA}$ of ρ_S such that $\big(U_S(g) \otimes U_A(g)\big)|\psi\rangle\langle\psi|_{SA}\big(U_S(g) \otimes U_A(g)\big)^\dagger = |\psi\rangle\langle\psi|_{SA}$. Then:

$$\begin{aligned}\rho_S &= \mathrm{Tr}_A(|\psi\rangle\langle\psi|_{SA}) \\ &= \mathrm{Tr}_A\big(\big(U_S(g) \otimes U_A(g)\big)|\psi\rangle\langle\psi|_{SA}\big(U_S(g) \otimes U_A(g)\big)^\dagger\big) \\ &= U_S(g)\mathrm{Tr}_A(|\psi\rangle\langle\psi|_{SA})U_S^\dagger(g) = U_S(g)\rho_S U_S^\dagger(g).\end{aligned} \tag{71}$$

For the $\Rightarrow$ direction we assume there exists a (weakly) invariant state $\rho_S = U(g)\rho_S U_S^\dagger(g)$. Then by Lemma 4,

$$\rho_S = \bigoplus_\lambda p_\lambda \frac{\mathbb{I}_{\mathcal{M}_\lambda^S}}{d_\lambda} \otimes \rho_{\mathcal{N}_\lambda^S}\ . \tag{72}$$

Let $\mathcal{H}_A \simeq \bar{\mathcal{H}}_S$ carry the conjugate representation $U_A(g) \simeq \bar{U}_S(g)$. Then

$$\mathcal{H}_A \simeq \bar{\mathcal{H}}_S \simeq \bigoplus_\lambda \bar{\mathcal{N}}_\lambda^A \otimes \bar{\mathcal{M}}_\lambda^A, \tag{73}$$

where $\mathcal{N}_\lambda^A$ carries the representation $\bar{U}_\lambda$.

Now define the state

$$|\psi\rangle_{SA} = \bigoplus_\lambda \sqrt{p_\lambda} \sum_{i_\lambda} |i_\lambda\rangle_{\mathcal{M}_\lambda^S} |i_\lambda\rangle_{\bar{\mathcal{M}}_\lambda^A} \otimes |\psi^\lambda\rangle_{\mathcal{N}_\lambda^S,\bar{\mathcal{N}}_\lambda^A} \tag{74}$$

with $|\psi^\lambda\rangle_{\mathcal{N}_\lambda^S,\bar{\mathcal{M}}_\lambda^A}$ a purification of $\rho_{\mathcal{N}_\lambda^S}$, which always exists since $\dim(\mathcal{N}_\lambda^S) = \dim(\bar{\mathcal{N}}_\lambda^A)$.

This is a purification of ρ_S which is invariant under $U_S(g) \otimes U_A(g)$ by construction. □

SUPPLEMENTARY NOTE 4 – STRUCTURAL DETAILS ON QUANTUM PERMUTATIONS

F. Applicability of our formalism in second quantization: particle permutations

While the action of standard permutations in first quantization is given by

$$U(\pi)|x_1,\ldots,x_N\rangle = |x_{\pi^{-1}(1)},\ldots,x_{\pi^{-1}(N)}\rangle, \tag{75}$$

usually referred to as a *label* or *place* permutation, it can generally not be defined consistently this way on Fock states

$$|k_1,...,k_N\rangle_{\mathcal{F}} := a^\dagger_{k_1}...a^\dagger_{k_N}|0\rangle_{\mathcal{F}} \tag{76}$$

and the associated creation operators in second quantization. To see this, consider the parastatistical model from Example 2: given the state $\left(a_2^\dagger a_1^\dagger a_3^\dagger + a_2^\dagger a_3^\dagger a_1^\dagger\right)|0\rangle_{\mathcal{F}}$ then exchanging the positions of the first two creation operators gives $\left(a_1^\dagger a_2^\dagger a_3^\dagger + a_3^\dagger a_2^\dagger a_1^\dagger\right)|0\rangle_{\mathcal{F}} = 0$, entailing that place permutations are not unitary. Instead, standard permutations in second quantization, which are referred to as *particle* permutations, act by permuting the *modes.* For example, the particle permutation corresponding to exchanging modes 1 and 2 from above can be represented by a unitary operator $\bar{U}((12))$ that effectively exchanges the creation operators $a_1^\dagger$ and $a_2^\dagger$ and transforms $\left(a_2^\dagger a_1^\dagger a_3^\dagger + a_2^\dagger a_3^\dagger a_1^\dagger\right)|0\rangle_{\mathcal{F}}$ into $\left(a_1^\dagger a_2^\dagger a_3^\dagger + a_1^\dagger a_3^\dagger a_2^\dagger\right)|0\rangle_{\mathcal{F}} \neq 0$.

Following Stolt and Taylor [16] we define particle permutations by considering tuples $K = (k_1,\ldots,k_N)$ of modes, say momenta, that are pairwise distinct, $k_i \neq k_j$ for $i \neq j$, and we choose lexicographical order, assuming that $k_1 < k_2 < \ldots < k_N$. This is simply a convention to ensure we do not consider tuples $K \neq K'$ that have the same set of entries, but in different order. For our purposes, we are only concerned with the case of a single particle occupation per mode, as this suffices to derive all our results. We thus refer to [16] for the construction with different occupation numbers of the modes.

Now, for every K, we have a subspace $\mathcal{H}_K$ that is spanned by the state vectors $|k_{\pi(1)},\ldots,k_{\pi(N)}\rangle_{\mathcal{F}}$, for all permutations $\pi \in S_N$. This subspace carries a representation $\pi \mapsto \bar{U}_K(\pi)$ of S_N, which transforms creation operators $a^\dagger_{k_i}$ into $\bar{U}_K(\pi)a^\dagger_{k_i}\bar{U}_K(\pi)^\dagger = a^\dagger_{k_{\pi(i)}}$. The space $\mathcal{H}_K$ is of dimension less than or equal to $N!$, which in the case of Bosons or Fermions is one-dimensional.

The $\bar{U}_K(\pi)$ define reducible representations of S_N and we get a decomposition

$$\mathcal{H}_K = \bigoplus_\lambda \mathcal{H}_{\lambda,K}, \tag{77}$$

where $\mathcal{H}_{\lambda,K}$ carries the λ-irrep of S_N, and the sum is over all admissible Young diagrams. That is, as shown in [12], each irrep λ corresponding to an admissible Young diagram appears exactly once in this decomposition.

We can now consider an external system described by some Hilbert space $\mathcal{N}$, write

$$\mathcal{H}_K \otimes \mathcal{N} = \bigoplus_\lambda \mathcal{H}_{\lambda,K} \otimes \mathcal{N}. \tag{78}$$

Alternatively, the particles could carry some internal degree of freedom, such as spin, say with total spin s. In this case, we would expect that the creation operators $a_k^\dagger$ are replaced by creation operators $a^\dagger_{k,\sigma}$, where σ can take several different values (e.g. $\sigma \in \{-s,-s+1,\ldots,s\}$). Consider the special case that all particles are prepared in the same spin σ, as is the case in the experimental proposal by Roos et al. [14]. Then, for every fixed σ, the operators $\{a^\dagger_{k,\sigma}\}_k$ satisfy relations (30). Since states for different σ are orthogonal, we obtain a particle Hilbert space that is also of the form (78), where $\mathcal{N} \simeq \mathbb{C}^{2s+1}$. More generally, for every choice of spins $\sigma_1,\sigma_2,\ldots,\sigma_N$, there will be a subspace spanned by vectors $|(k_{\pi(1)},\sigma_{\pi(1)}),\ldots,(k_{\pi(N)},\sigma_{\pi(N)})\rangle_{\mathcal{F}}$ which individually behaves like the paraparticle subspace without internal degrees of freedom described above. This also gives us a Hilbert space of the form (78), where now $\mathcal{N} \simeq \mathbb{C}^{(2s+1)^N}$. In all cases, we obtain a multiplicity space as in Eq. (16) and Eq. (17) that allows us to construct quantum permutations by conditioning on observables supported on it.

G. More general quantum permutation groups

In principle, we could have started from a more general version in the definition of quantum permutations: instead of conditioning only the argument $\pi_{\lambda,j}$ of the representation map $\pi \mapsto e^{i\theta(\pi)}U(\pi)$ on λ, j, we could also condition the map itself on λ, j as in Eq. (18) in the main text: $\pi \mapsto W_{\lambda,j}(\pi) := e^{i\theta_{\lambda,j}(\pi)}U(\pi)$. To do so, we will require that the *unconditional* special case should still reproduce the standard permutations: applying the same permutation π in every branch must projectively act like $U(\pi)$; that is, $V(\pi,\pi,\ldots,\pi)\rho V(\pi,\pi,\ldots,\pi)^\dagger = U(\pi)\rho U(\pi)^\dagger$.

As in the proof of Lemma 2 in the main text, $V(\boldsymbol{\sigma})V(\boldsymbol{\pi}) = \omega(\boldsymbol{\sigma},\boldsymbol{\pi})V(\boldsymbol{\sigma\pi})$ then implies

$$\omega(\boldsymbol{\sigma},\boldsymbol{\pi}) = e^{i\theta_{\lambda,j}(\sigma_{\lambda,j})}e^{i\theta_{\lambda,j}(\pi_{\lambda,j})}e^{-i\theta_{\lambda,j}(\sigma_{\lambda,j}\pi_{\lambda,j})} \tag{79}$$

for all λ, j. Assuming $Q > 1$ without loss of generality, suppose that $\boldsymbol{\sigma}'$ and $\boldsymbol{\pi}'$ are elements of S_N^Q that agree with $\boldsymbol{\sigma}$ and $\boldsymbol{\pi}$ on at least one entry (λ, j), i.e. there exists some (λ_0, j_0) such that $\sigma_{\lambda_0,j_0} = \sigma'_{\lambda_0,j_0}$ and $\pi_{\lambda_0,j_0} = \pi'_{\lambda_0,j_0}$. Then, we have

$$\begin{aligned}\omega(\boldsymbol{\sigma},\boldsymbol{\pi}) &= e^{i\theta_{\lambda_0,j_0}(\sigma_{\lambda_0,j_0})}e^{i\theta_{\lambda_0,j_0}(\pi_{\lambda_0,j_0})}e^{-i\theta_{\lambda_0,j_0}(\sigma_{\lambda_0,j_0}\pi_{\lambda_0,j_0})}\\ &= e^{i\theta_{\lambda_0,j_0}(\sigma'_{\lambda_0,j_0})}e^{i\theta_{\lambda_0,j_0}(\pi'_{\lambda_0,j_0})}e^{-i\theta_{\lambda_0,j_0}(\sigma'_{\lambda_0,j_0}\pi'_{\lambda_0,j_0})}\\ &= \omega(\boldsymbol{\sigma}',\boldsymbol{\pi}').\end{aligned} \tag{80}$$

Let $\boldsymbol{\sigma}'', \boldsymbol{\pi}''$ be arbitrary elements of S_N^Q. Then we can always find some pair $\boldsymbol{\sigma}', \boldsymbol{\pi}'$ that agree with the pair $\boldsymbol{\sigma}, \boldsymbol{\pi}$ in at least one entry, say λ_0, j_0, and that also agrees with the pair $\boldsymbol{\sigma}'', \boldsymbol{\pi}''$ in at least one entry, say λ_1, j_1. Thus,

$$\begin{aligned}\omega(\boldsymbol{\sigma}', \boldsymbol{\pi}') &= e^{i\theta_{\lambda_1,j_1}(\sigma'_{\lambda_1,j_1})} e^{i\theta_{\lambda_1,j_1}(\pi'_{\lambda_1,j_1})} e^{-i\theta_{\lambda_1,j_1}(\sigma'_{\lambda_1,j_1}\pi_{\lambda_1,j_1})} \\ &= e^{i\theta_{\lambda_1,j_1}(\sigma''_{\lambda_1,j_1})} e^{i\theta_{\lambda_1,j_1}(\pi''_{\lambda_1,j_1})} e^{-i\theta_{\lambda_1,j_1}(\sigma''_{\lambda_1,j_1}\pi''_{\lambda_1,j_1})} \\ &= \omega(\boldsymbol{\sigma}'', \boldsymbol{\pi}''), \end{aligned} \tag{81}$$

so $\omega(\boldsymbol{\sigma}, \boldsymbol{\pi}) = \omega(\boldsymbol{\sigma}'', \boldsymbol{\pi}'') = \omega$ is a constant that does not depend on $\boldsymbol{\sigma}$ or $\boldsymbol{\pi}$. The special case $\pi_{\lambda,j} = \mathbb{I}$ shows that $\omega = e^{i\theta}$ for $\theta := \theta(\mathbb{I})$, and hence $\boldsymbol{\pi} \mapsto e^{-i\theta} V(\boldsymbol{\pi})$ is a linear unitary representation. Furthermore, $\pi \mapsto e^{-i\theta} e^{i\theta_{\lambda,j}(\pi)}$ is a linear one-dimensional representation of S_N, i.e. either the trivial or the sign representation, depending on λ, j.

Now, even though this more general definition still qualifies as a projective representation of S_N, its action does not generally reduce to that of standard permutations in the unconditional special case $\boldsymbol{\pi} = (\pi, \pi, \ldots, \pi)$. To see this, assume we have such a general map $\boldsymbol{\pi} \mapsto V(\boldsymbol{\pi})$. Then, setting $e^{i\theta} = 1$, there exist pairs λ_1, j_1 and λ_2, j_2, where at least $\lambda_1 \neq \lambda_2$ or $j_1 \neq j_2$, such that $W_{\lambda_1,j_1}(\pi_{\lambda_1,j_1}) = U(\pi_{\lambda_1,j_1})$ and $W_{\lambda_2,j_2}(\pi_{\lambda_2,j_2}) = \mathrm{sgn}(\pi_{\lambda_2,j_2}) U(\pi_{\lambda_2,j_2})$. For any $\pi \in S_N$, set $\boldsymbol{\pi} := (\pi, ..., \pi)$ and define

$$A(\pi) := U(\pi)^\dagger V(\boldsymbol{\pi}) = V(\boldsymbol{\pi}) U(\pi)^\dagger = \sum_{\lambda,j} s_{\lambda,j}(\pi) \mathbb{I}_\lambda \otimes P_{\lambda,j}, \tag{82}$$

where $s_{\lambda,j}(\pi) \in \{1, \mathrm{sgn}(\pi)\}$. Furthermore, note that, since $P_{\lambda,j}$ is a rank-1 projector, for every λ, j there exists an element $|\psi_{\lambda,j}\rangle$ such that $\mathbb{I}_\lambda \otimes P_{\lambda,j} |\psi_{\lambda,j}\rangle = |\psi_{\lambda,j}\rangle$. This implies

$$A(\pi)(|\psi_{\lambda_1,j_1}\rangle + |\psi_{\lambda_2,j_2}\rangle) = |\psi_{\lambda_1,j_1}\rangle + \mathrm{sgn}(\pi) |\psi_{\lambda_2,j_2}\rangle, \tag{83}$$

showing that $A(\pi) \neq e^{i\theta(\pi)} \mathbb{I}$ for any $\theta(\pi)$, as it causes relative phases, and hence $V(\boldsymbol{\pi}) \neq e^{i\theta(\pi)} U(\pi)$. Therefore, $V(\boldsymbol{\pi})$ does not projectively act like $U(\pi)$.

H. Proof of Theorem 3 in the main text

Assuming the Bosonic case $U(\pi)$ (the Fermionic case $\mathrm{sgn}(\pi) U(\pi)$ works in the same way), recall the form of the quantum permutations of equation (16) in the main text. They are given with respect to a fixed basis for each multiplicity space $\mathcal{N}_\lambda$. In order to get rid of this, we define

$$\begin{aligned} U^{(\mathbf{R})}(\boldsymbol{\pi}) &:= \left(\bigoplus_\mu \mathbb{I}_{\mathcal{M}_\lambda} \otimes R_\mu \right) U(\boldsymbol{\pi}) \left(\bigoplus_\nu \mathbb{I}_{\mathcal{M}_\lambda} \otimes R_\nu^\dagger \right) \\ &= \sum_{\lambda,j} U_\lambda(\pi_{\lambda,j}) \otimes R_\lambda P_{\lambda,j} R_\lambda^\dagger \end{aligned} \tag{84}$$

for arbitrary unitaries $R_\lambda \in \mathrm{U}(n_\lambda)$ and any λ. With this definition we have $U(\boldsymbol{\pi}) = U^{(\mathbb{I})}(\boldsymbol{\pi})$, i.e. $R_\lambda = \mathbb{I}_{\mathcal{N}_\lambda}$.

Now, we have seen that the requirement $[U^{(\mathbb{I})}(\boldsymbol{\pi})_S \otimes \mathbb{I}_A, \rho_{SA}] = 0$ for all $\boldsymbol{\pi} \in S_N^Q$ implies

$$\begin{aligned} \rho_{SA} &= p_{\mathrm{Bos}}\, \rho_{\mathrm{Bos},A} \oplus \bigoplus_{j,\lambda \neq \lambda_{\mathrm{Bos}}} p_{\lambda,j} \frac{\mathbb{I}_{\lambda,j}}{d_\lambda} \otimes \rho_A^{(\lambda,j)} \\ &= p_{\mathrm{Bos}}\, \rho_{\mathrm{Bos},A} \oplus \bigoplus_{\lambda \neq \lambda_{\mathrm{Bos}}} \frac{\mathbb{I}_{\mathcal{M}_\lambda}}{d_\lambda} \otimes \sum_j p_{\lambda,j} P_{\lambda,j} \otimes \rho_A^{(\lambda,j)}, \end{aligned} \tag{85}$$

where d_λ is the dimension of $\mathcal{H}_{\lambda,j}$, and the state of system $\mathcal{N}_\lambda$ is diagonal in the basis associated to the projectors $P_{\lambda,j}$. Demanding invariance with respect to all possible quantum permutations without a fixed basis means that we need to impose the stronger condition $[U^{(\mathbf{R})}(\boldsymbol{\pi})_S \otimes \mathbb{I}_A, \rho_{SA}] = 0$ for all $\boldsymbol{\pi} \in S_N^Q$ and all R_λ. Therefore, we require

$$(U^{(\mathbf{R})}(\boldsymbol{\pi})_S \otimes \mathbb{I}_A) \rho_{SA} (U^{(\mathbf{R})}(\boldsymbol{\pi})_S \otimes \mathbb{I}_A)^\dagger = \rho_{SA}, \tag{86}$$

which, due to Eq. (84), is equivalent to

$$[U^{(\mathbb{I})}(\boldsymbol{\pi})_S \otimes \mathbb{I}_A, \underbrace{(\oplus_\mu \mathbb{I}_{\mathcal{M}_\mu} \otimes R_\mu \otimes \mathbb{I}_A) \rho_{SA} (\oplus_\nu \mathbb{I}_{\mathcal{M}_\nu} \otimes R_\nu^\dagger \otimes \mathbb{I}_A}_{=: \rho_{SA}^{(\mathbf{R})}}] = 0, \tag{87}$$

so $\rho_{SA}^{(\mathbf{R})}$ must be of the same form as ρ_{SA} in Eq. (85) for all R_λ. We obtain

$$\begin{aligned} \rho_{SA}^{(\mathbf{R})} =& p_{\mathrm{Bos}} (R_{\mathrm{Bos}} \otimes \mathbb{I}_A) \rho_{\mathrm{Bos},A} (R_{\mathrm{Bos}}^\dagger \otimes \mathbb{I}_A) \\ &\oplus \bigoplus_{\lambda \neq \lambda_{\mathrm{Bos}}} \frac{\mathbb{I}_{\mathcal{M}_\lambda}}{d_\lambda} \otimes \sum_j p_{\lambda,j} R_\lambda P_{\lambda,j} R_\lambda^\dagger \otimes \rho_A^{(\lambda,j)}, \end{aligned} \tag{88}$$

and see that this immediately implies

$$R_\lambda \left(\sum_j p_{\lambda,j} P_{\lambda,j} \right) R_\lambda^\dagger = \sum_j p'_{\lambda,j} P_{\lambda,j} \tag{89}$$

for some $p'_{\lambda,j}$ after tracing out the ancillary system A. This means that $R_\lambda (\sum_j p_{\lambda,j} P_{\lambda,j}) R_\lambda^\dagger$ must be diagonal with respect to the $P_{\lambda,j}$-basis for all unitary conjugations by R_λ, which is only the case when $p_{\lambda,j}$ does not depend on j for any $\lambda \neq \lambda_{\mathrm{Bos}}$. Due to $\dim(\mathcal{N}_\lambda) = n_\lambda$, we have $p_{\lambda,j} = p_\lambda / n_\lambda$, such that $p_{\mathrm{Bos}} + \sum_{\lambda \neq \mathrm{Bos}} p_\lambda = 1$. Note that this is only possible if $n_\lambda < \infty$. This already proves that in the case that at least one of the N_λ for $\lambda \neq \lambda_{\mathrm{Bos}}$ is infinite-dimensional, there is no invariant state ρ_{SA}.

Let us continue with the case that all $n_\lambda < \infty$ for $\lambda \neq \lambda_{\mathrm{Bos}}$. With this, let us also make the claim that basis-independent invariance implies that for any $\lambda \neq \lambda_{\mathrm{Bos}}$, the states $\rho_A^{(\lambda,j)}$ cannot depend on j either. To show this, we first note that, since $P_{\lambda,j}$ are rank-1 projectors of a projective measurement, we can find an orthogonal

basis $\{|j\rangle_\lambda\}_j$ of $\mathcal{N}_\lambda$ such that $P_{\lambda,j} = |j\rangle\langle j|_\lambda$. Then, given unitaries R_λ with $|j^{(\mathbf{R})}\rangle_\lambda := R_\lambda|j\rangle_\lambda$, we have

$$\begin{aligned}\rho_{SA}^{(\mathbf{R})} =& p_{\mathrm{Bos}}\,(R_{\mathrm{Bos}}\otimes\mathbb{I}_A)\rho_{\mathrm{Bos},A}(R_{\mathrm{Bos}}^\dagger\otimes\mathbb{I}_A)\\ &\oplus\bigoplus_{\lambda\neq\lambda_{\mathrm{Bos}}} p_\lambda\frac{\mathbb{I}_{\mathcal{M}_\lambda}}{d_\lambda n_\lambda}\otimes\sum_j |j^{(\mathbf{R})}\rangle\langle j^{(\mathbf{R})}|_\lambda\otimes\rho_A^{(\lambda,j)}.\end{aligned} \tag{90}$$

Now, if $[U^{(\mathbb{I})}(\boldsymbol{\pi})_S\otimes\mathbb{I}_A,\rho_{SA}^{(\mathbf{R})}] = 0$ holds, then it must follow that $\rho_{SA}^{(\mathbf{R})}$ has the form of Eq. (85) when expressed in the $P_{\lambda,j}$-basis. Due to $|j^{(\mathbf{R})}\rangle_\lambda = \sum_k (R_\lambda)_{kj}|k\rangle_\lambda$, we find

$$\begin{aligned}\rho_{SA}^{(\mathbf{R})} =& p_{\mathrm{Bos}}\,(R_{\mathrm{Bos}}\otimes\mathbb{I}_A)\rho_{\mathrm{Bos},A}(R_{\mathrm{Bos}}^\dagger\otimes\mathbb{I}_A)\\ &\oplus\bigoplus_{\lambda\neq\lambda_{\mathrm{Bos}}} p_\lambda\frac{\mathbb{I}_{\mathcal{M}_\lambda}}{d_\lambda n_\lambda}\otimes\sum_{k,l}|k\rangle\langle l|_\lambda\otimes\sum_j (R_\lambda)_{kj}(R_\lambda^\dagger)_{jl}\rho_A^{(\lambda,j)},\end{aligned} \tag{91}$$

and thus, for all R_λ we obtain the condition

$$\sum_j (R_\lambda)_{kj}(R_\lambda^\dagger)_{jl}\rho_A^{(\lambda,j)} = 0, \tag{92}$$

whenever $k\neq l$. In particular, let us now choose R_λ to be rotations of the planes spanned by the first and the i-th coordinate axes by an angle α. Then we have $(R_\lambda)_{11} = (R_\lambda)_{ii} = \cos\alpha$, $(R_\lambda)_{1i} = -(R_\lambda)_{i1} = \sin\alpha$ and all other entries being equal to those of the identity. With this, the condition above implies $\rho_A^{(\lambda,1)} = \rho_A^{(\lambda,i)}$ for any i, and as a consequence, $\rho_A^{(\lambda,j)}$ must not depend on j which shows the claim.

Finally, a state ρ_{SA} which is invariant under all possible quantum permutations must have the form

$$\begin{aligned}\rho_{SA} &= p_{\mathrm{Bos}}\,\rho_{\mathrm{Bos},A}\oplus\bigoplus_{j,\lambda\neq\lambda_{\mathrm{Bos}}} p_\lambda\frac{\mathbb{I}_{\lambda,j}}{d_\lambda n_\lambda}\otimes\rho_A^{(\lambda)}\\ &= p_{\mathrm{Bos}}\,\rho_{\mathrm{Bos},A}\oplus\bigoplus_{\lambda\neq\lambda_{\mathrm{Bos}}} p_\lambda\frac{\mathbb{I}_{\mathcal{M}_\lambda\otimes\mathcal{N}_\lambda}}{d_\lambda n_\lambda}\otimes\rho_A^{(\lambda)},\end{aligned} \tag{93}$$

with non-bosonic post-measurement states

$$\frac{\mathbb{I}_{\mathcal{M}_\lambda\otimes\mathcal{N}_\lambda}}{d_\lambda n_\lambda}\otimes\rho_A^{(\lambda)}. \tag{94}$$

Tracing out the ancillary system A, we obtain the completely mixed state for any $\lambda\neq\lambda_{\mathrm{Bos}}$.

SUPPLEMENTARY NOTE 5 – QUANTUM REFERENCE FRAMES

I. Application to Bosons and Fermions

In this subsection, we provide a swift introduction to Quantum Reference Frames (QRFs), thereby motivating the form of QRF transformations as conditional transformations, and we show how these can be applied in the context of Bosons and Fermions. In particular, we will see that the QRF transformation picture corresponds to the implementation of some symmetry *on the level of observables*: only observables invariant under the symmetry are deemed measurable, but all quantum states are allowed as descriptions of preparation procedures. This is the convention of invariance that we adopt for this subsection (see the Methods section in the main text).

Let us begin with a simple illustrative example of QRF transformations. Consider three particles A, B, C in one dimension subject to translation-invariance, following the analysis of Giacomini et al. [17]. Suppose that the state in position representation is a product state,

$$|\psi_{|A}\rangle = |0\rangle_A\otimes\frac{1}{\sqrt{2}}\left(|1\rangle_B+|2\rangle_B\right)\otimes|3\rangle_C \tag{95}$$

(to prevent some mathematical difficulties, let us assume that all positions are integers, like on a lattice). This has sometimes been said to describe the quantum state "as seen by A", because A is placed in the origin. Under this intuition, we would obtain the state "as seen by C" by translating everything by -3,

$$|\psi_{|C}\rangle = |-3\rangle_A\otimes\frac{1}{\sqrt{2}}\left(|-2\rangle_B+|-1\rangle_B\right)\otimes|0\rangle_C. \tag{96}$$

But what would the state "as seen by B" look like? No proper translation can move B to the origin. The idea is now to translate in superposition: one of the branches of $\psi_{|A}$ is translated by -1, and the other by -2, and this is done coherently, obtaining

$$|\psi_{|B}\rangle = |0\rangle_B\otimes\frac{1}{\sqrt{2}}\left(|-1\rangle_A\otimes|2\rangle_C+|-2\rangle_A\otimes|1\rangle_C\right). \tag{97}$$

This is typically interpreted as having the particles A, B and C themselves as quantum reference frames, i.e. quantum analogues of classical rods or clocks, and notions of entanglement being frame-dependent. Here we take a different perspective described in [18, 19]: there is an algebra of observables X that can be measured under a certain condition of translation-invariance, and we have

$$\langle\psi_{|A}|X|\psi_{|A}\rangle = \langle\psi_{|B}|X|\psi_{|B}\rangle = \langle\psi_{|C}|X|\psi_{|C}\rangle \tag{98}$$

for all of them. This means that the $\psi_{|S}$ are alternative, equally valid descriptions of one and the same quantum state. Choosing one representation over another can be interpreted as choosing a quantum coordinate system; in this case, a system that aligns the origin with one of the particles. A *QRF transformation* is a unitary transformation that maps from one quantum coordinate system to another. The starting point are the global translations T_d, translating the global state by distance d, acting as

$$V(T_d)\left(|a\rangle_A|b\rangle_B|c\rangle_C\right) = |a+d\rangle_A|b+d\rangle_B|c+d\rangle_C. \tag{99}$$

QRF transformations are coherently controlled versions of this. For example, mapping from the quantum coordinate system relative to A to the one relative to B is achieved by the map

$$U_{A\to B} = \sum_{d\in\mathbb{Z}} V(T_{-d})P_d, \tag{100}$$

where $\{P_d\}_{d\in\mathbb{Z}}$ is a projective measurement of the distance d between particles B and A, i.e.

$$P_d|a\rangle_A|b\rangle_B|c\rangle_C = \begin{cases} |a\rangle_A|b\rangle_B|c\rangle_C & \text{if } b-a=d \\ 0 & \text{otherwise.} \end{cases} \tag{101}$$

It implements the transformation from QRF A to B, i.e. $U_{A\to B}|\psi_{|A}\rangle = |\psi_{|B}\rangle$. It is a global translation, conditioned on a translation-invariant quantity, namely on the relative distance between A and B. QRF transformations in general are understood as coherently controlled classical reference frame changes [20], where the controlling branches are themselves defined in a frame-independent way [21], or as state-dependent gauge transformations [22].

The states $\psi_{|S}$ relative to the different subsystems S are all physically equivalent to the invariant state $\rho := \int_{\mathcal{U}} U|\psi_{|S}\rangle\langle\psi_{|S}|U^\dagger\, dU$, where $\mathcal{U}$ is the group of QRF transformations; see [18] for details. This construction is closely related to the *perspective-neutral framework*, where an invariant pure state $|\psi_{\rm phys}\rangle$ on a subspace $\mathcal{H}_{\rm phys}$ with in general redefined inner product takes this role [23–26].

The similarity with our definition of quantum permutations, Definition 1 in the main text, is clear: we apply a symmetry transformation conditioned on an invariant quantity. Hence, quantum permutations can be interpreted as QRF transformations, or, slightly more accurately, quantum coordinate transformations. They transform between different quantum conventions for labeling the particles. As a result of Theorem 2 in the main text, we find that there exist exactly two groups of quantum permutations, Bosonic and Fermionic ones, depending on the choice of projectively equivalent representations. Bosonic (Fermionic) QRF transformations will leave all Bosonic (Fermionic) observables invariant, thus providing groups of transformations between different but physically equivalent descriptions of Bosonic (Fermionic) quantum states.

For example, for $N=2$ Fermions, the quantum permutations are given by $V_-(\boldsymbol{\pi}) = \sum_{\lambda,j} {\rm sgn}(\pi_{\lambda,j})U(\pi_{\lambda,j})(\mathbb{I}_\lambda \otimes P_{\lambda,j})$. Hence the exchange of the two particles, denoted $(1,2)$, is represented as $V_-(\boldsymbol{\pi} = \overrightarrow{(1,2)})$, where $\overrightarrow{(1,2)}_{\lambda,j} = (1,2)$ for all λ, j, which simplifies to $V(\overrightarrow{(1,2)}) = {\rm sgn}((1,2))U((1,2)) = -U(\text{swap})$. The measurable observables are all the operators X that are fully supported on the antisymmetric subspace. Suppose we have a quantum state that we describe as $|\psi_{\rm asc}\rangle = |1,4\rangle$, where by convention we decided to describe the particles in ascending order. Then we could equivalently decide to describe them in descending order via $|\psi_{\rm des}\rangle := V(\overrightarrow{(1,2)})|\psi_{\rm asc}\rangle = -|4,1\rangle$, and then

$$\langle\psi_{\rm asc}|X|\psi_{\rm asc}\rangle = \langle\psi_{\rm des}|X|\psi_{\rm des}\rangle \tag{102}$$

for all measurable observables X. The states $|\psi_{\rm asc}\rangle$ and $|\psi_{\rm des}\rangle$ are therefore physically equivalent descriptions. These are not permutationally invariant, however the state $|\psi_{\rm phys}\rangle = \frac{1}{\sqrt{2}}(|1,4\rangle - |4,1\rangle)$ is invariant (not just as a state, but also as a vector) under $V((1,2))$ and is physically equivalent to the states $|\psi_{\rm asc}\rangle$ and $|\psi_{\rm des}\rangle$. From this simple example, it is not obvious why the minus sign is significant in the definition of $V(\overrightarrow{(1,2)})$. Indeed, for unconditional permutations (i.e. $\pi_{\lambda,j} = \pi_{\lambda',j'}$), the choice of representation $-U(\text{swap})$ occurring in $V_-(\boldsymbol{\pi})$ is equivalent up to a global phase (hence also physically equivalent) to the choice $U(\text{swap})$. The choice of representation $-U(\text{swap})$ becomes significant for conditional permutations as becomes evident in a slightly more involved example:

Example 5. *Suppose that we have $N=2$ Fermions, such that the measurable observables are those supported on the antisymmetric subspace. Representing the swap of the two particles by $e^{i\theta}U(\text{swap})$ with some phase θ, consider the operator $X = |\{2,3\}\rangle\langle\{1,4\}|_-$, where $|\{x_1,x_2\}\rangle_- := \frac{1}{\sqrt{2}}\left(|x_1,x_2\rangle - |x_2,x_1\rangle\right)$, and a state $|\psi\rangle = \frac{1}{\sqrt{2}}\left(|2,3\rangle + |1,4\rangle\right)$. We apply the following quantum permutation: we swap the two particles if their distance is larger than 2, and otherwise we do nothing. This maps the state $|\psi\rangle$ to $|\psi'\rangle = \frac{1}{\sqrt{2}}\left(|2,3\rangle + e^{i\theta}|4,1\rangle\right)$, and*

$$\langle\psi|X|\psi\rangle = \langle\psi'|X|\psi'\rangle \tag{103}$$

if and only if $e^{i\theta} = -1$, explaining the choice of phase above. It follows from Theorem 2 in the main text that this equation is true for all X on the antisymmetric subspace. Hence, this quantum permutation maps between physically equivalent descriptions of the quantum state.

In two upcoming publications, we will further elaborate on this usage of the internal quantum reference frame formalism for Bosons and Fermions: we will show how it clarifies the question of entanglement of indistinguishable particles in a particularly transparent way [27], and that it is a simple example of a natural generalization of the perspective-neutral framework [24, 25] where internal quantum reference frames need not correspond to subsystems [28].

J. Implications for quantum covariance principles

An ambitious goal of the internal quantum reference frames (QRF) research program is to obtain physical predictions for some scenarios that would ultimately be described by quantum gravity. As an example, consider a thought experiment by de la Hamette et al. [29]. We

have a large mass (for the sake of the argument, planet Earth) in a superposition of locations relative to some reference system, and a test particle moving in the resulting gravitational field. We cannot appeal to the known physical laws to predict the movement of the test particle, since we expect to obtain some sort of superposition of gravitational fields and spacetime geometries which would fall into the regime of quantum gravity. However, the authors of [29] suggest that it makes sense to postulate an "extended symmetry principle" described as the "covariance of dynamical laws under quantum coordinate transformations". Based on this principle, one may attempt to transform into a quantum coordinate system where Earth's location and hence the resulting gravitational field are definite, but the test particle is in a superposition state. Based on the generalized covariance principle, one would then solve the quantum equations of motion of the test particle in the corresponding classical gravitational field and transform back, obtaining a description of the movement of the test particle in a superposition of gravitational fields.

The results of our work show a subtle difficulty of such approaches: quantum coordinate transformations do not necessarily preserve all physical predictions, but only some of them. Before we proceed, let us remark here that the interaction of the reference system with the gravitational field of the planet is considered to be negligible by the authors of [29], which is a necessary assumption to establish their result. While, as a consequence of that, the scenario they consider is not affected by the subtleties we raise, it is nevertheless instructive to analyze it in more detail, in order to illustrate our point.

Recall that the QRF formalism can be interpreted as implementing some symmetry on the level of observables (in the terminology of Section I and the Methods section in the main text), while allowing arbitrary non-invariant density operators to describe quantum states. As the authors discuss, their argumentation applies in a physical regime where it makes sense to speak of the positions of the involved physical systems and the associated global translations. The initial state of a reference system R, the mass M and the test particle S reads

$$|\psi\rangle_{RMS}^{(R)} = |0\rangle_R \frac{1}{\sqrt{2}}\left(|x_M^{(1)}\rangle_M + |x_M^{(2)}\rangle_M\right)|x_S\rangle_S, \quad (104)$$

where x_T denotes the position of subsystem $T \in \{R, M, S\}$ and $|\psi\rangle_T$ is the state of subsystem T. Now they use the QRF transformations described in Subsection I above to move into a quantum coordinate system where the large mass M is in a definite position:

$$|\psi\rangle_{MRS}^{(M)} = |0\rangle_M \frac{1}{\sqrt{2}}\left(e^{-iPx_M^{(1)}}|-x_M^{(1)}\rangle_R|x_S - x_M^{(1)}\rangle_S + e^{-iPx_M^{(2)}}|-x_M^{(2)}\rangle_R|x_S - x_M^{(2)}\rangle_S\right), \quad (105)$$

where $P = 0$ in [29]. Note that the transition from (104) to (105) is achieved by applying the representation of the translation group (99) to the initial state branch by branch. However, there are many such representations V_P, one for every total momentum P:

$$V_P(T_d)|a\rangle|b\rangle|c\rangle = e^{iPd}|a+d\rangle|b+d\rangle|c+d\rangle. \quad (106)$$

In (105) above, we give the general result if the representation according to some value of P is chosen.

From this for $P = 0$, the authors of [29] use standard methods to calculate the time-evolved state $|\psi(t)\rangle_{MRS}^{(M)}$, under the assumption that the reference system R is far away enough such that it does not feel the gravitational pull of M. Let us drop this assumption here. We obtain

$$|\psi(t)\rangle_{MRS}^{(M)} = |0\rangle_M \frac{1}{\sqrt{2}}\left(e^{-i\Phi^{(1)}}e^{-iPx_M^{(1)}}|-x_M^{(1)} + \delta x^{(1)}(t)\rangle_R|\tilde{x}_S^{(1)}(t)\rangle_S + e^{-i\Phi^{(2)}}e^{-iPx_M^{(2)}}|-x_M^{(2)} + \delta x^{(2)}(t)\rangle_R|\tilde{x}_S^{(2)}(t)\rangle_S\right), \quad (107)$$

where $\tilde{x}_S^{(i)}(t) = x_S(t) - x_M^{(i)}$, and $\delta x^{(i)}(t)$ denotes the position of the reference system R at time t in branch i, where the movement originates in the gravitational pull from M. We expect that $\delta x^{(i)} \approx 0$ if R is very far away from M in branch i, which is the case considered in [29]. Then the authors of [29] use the inverse QRF transformation to map back into R's quantum coordinate system. In our more general case, doing so yields

$$|\psi(t)\rangle_{RMS}^{(R)} = |0\rangle_R \frac{1}{\sqrt{2}}\left(e^{-iP\delta x^{(1)}(t)}e^{-i\Phi^{(1)}}|x_M^{(1)} - \delta x^{(1)}(t)\rangle_M|\tilde{x}_S^{(1)}(t) + x_M^{(1)} - \delta x^{(1)}(t)\rangle_S + e^{-iP\delta x^{(2)}(t)}e^{-i\Phi^{(2)}}|x_M^{(2)} - \delta x^{(2)}(t)\rangle_M|\tilde{x}_S(t) + x_M^{(2)} - \delta x^{(2)}(t)\rangle_S\right). \quad (108)$$

Hence, the extended symmetry principle predicts entanglement between the test particle S and the mass M, and

a relative phase of the corresponding quantum state (108) of

$$\Delta\varphi := \Phi^{(2)} - \Phi^{(1)} + P\left(\delta x^{(2)}(t) - \delta x^{(1)}(t)\right). \tag{109}$$

Unless $\delta x^{(1)}(t) = \delta x^{(2)}(t)$ (which holds for the scenario in [29], where both are zero), the predicted relative phase depends on P, which labels the choice of representation of the translation group. Consequently, the total momentum P attains physical significance. Let us contrast this with a physical postulate formulated in [29]: *"Physical laws retain their form under quantum coordinate transformations."* What the calculation above shows is that this cannot literally be true for *all* quantum coordinate transformations, because some predictions depend on the chosen representation, which is labelled by P. Therefore, we suggest the following modification of the postulate:

Physical laws retain their form under a suitable representation of the quantum coordinate transformation group.

The extended covariance principle of [29] is but one example of a larger class of quantum generalizations of symmetry principles considered in the literature, including also, for example, proposals for a quantum version of the equivalence principle [30–32], or a notion of quantum conformal symmetries [33]. Our results underline the predictive power of such principles (we use them to rule out parastatistics), but they also motivate some caution: only *some* quantum coordinate transformations will in general preserve the physical predictions.

This is demonstrated by our results on the permutation group. For N Fermions, we have shown that all physical predictions (that is, the expectation values of antisymmetric observables) are invariant under the sign representation

$$V_-(\boldsymbol{\pi}) = \sum_{\lambda,j} \mathrm{sgn}(\pi_{\lambda,j}) U_\lambda(\pi_{\lambda,j}) \otimes P_{\lambda,j}, \tag{110}$$

but not under the standard representation

$$V_+(\boldsymbol{\pi}) = \sum_{\lambda,j} U_\lambda(\pi_{\lambda,j}) \otimes P_{\lambda,j}, \tag{111}$$

of the quantum permutation group. Indeed, the latter type of quantum coordinate maps leads to experimentally detectable physical consequences, as described in the scheme by Roos et al. [14] for the detection of the Fermionic exchange phase. Hence, additional physical arguments are in general needed to obtain the correct gauge phases [34–36] for the construction of quantum coordinate transformations.

SUPPLEMENTARY NOTE 6 – RELATION TO OTHER NOTIONS OF SYMMETRY IN PREVIOUS WORK

In this section, we describe how our notions of complete invariance and of invariance under quantum permutations (and their generalizations to other groups) are related to other notions of symmetry that have appeared in the literature: to strong/weak symmetry, gauge symmetries, and the notions of coherent vs. incoherent twirling for quantum reference frames.

K. Strong versus weak and exact versus average symmetry

The requirement that a density operator ρ_S has full support on a subspace transforming under a one-dimensional representation of a compact group G, i.e. a subspace of the form $\mathcal{M}_\lambda \otimes \mathcal{N}_\lambda$ with $\mathcal{M}_\lambda \simeq \mathbb{C}$, is sometimes known as *strong symmetry* or *exact symmetry* [37, 38]. In this case every element $|\psi_i\rangle$ in an ensemble $\{|\psi_i\rangle\}_i$ corresponding to the density operator $\rho_S = \sum_i p_i |\psi_i\rangle\langle\psi_i|_S$ is such that $|\psi_i\rangle$ transforms under the same one-dimensional irreducible representation of G for every element in the ensemble: $U_S(g)|\psi_i\rangle_S = e^{i\theta(g)}|\psi_i\rangle_S$ for all $|\psi_i\rangle_S \in \{|\psi_i\rangle_S\}_i$. This is equivalent to the requirement $U_S(g)\rho_S = e^{i\theta(g)}\rho_S$.

This notion is contrasted to the standard notion of invariance of a density operator, which just requires that the density operator is invariant: $U(g)\rho_S U^\dagger(g) = \rho_S$, and does not require individual elements in its decomposition to be invariant. Thus the density operator, interpreted as a proper mixture, is invariant as a statistical object, even though the individual elements of the ensemble need not be invariant. This standard invariance is sometimes known as *weak symmetry* or *average symmetry*, in contrast to the strong/exact symmetry described above.

In this language, Theorem 1 in the main text (generalized to arbitrary compact groups in Theorem 1) establishes that complete invariance of ρ_S is equivalent to exact symmetry of ρ_S.

The distinction between exact and average symmetry was first applied to Lindbladians in the literature on open quantum systems. Definition 4 of [39] states that for a strongly symmetric state $\rho_S = U_S(g)\rho_S$ there exists a purification $|\psi\rangle_{SA}$ which is invariant (as a ray) under $U_S(g) \otimes \mathbb{I}_A$. Our Theorem 1 establishes the opposite direction of this statement also, showing that strong symmetry entails that every purification (and indeed every extension) of the state is completely invariant.

The novel aspect of the present work, with regards to the notion of complete invariance, is its application to permutation invariance, thereby ruling out parastatistics. In the open systems literature it is typically applied to issues such as spontaneous symmetry breaking, typically involving $\mathbb{Z}_2$ or U(1) symmetry.

L. Complete invariance from local gauge symmetry

At first glance, the requirement of complete invariance, namely invariance of an extension ρ_{SA} under $(U_S(g) \otimes \mathbb{I}_A) \bullet \left(U_S^\dagger(g) \otimes \mathbb{I}_A\right)$, may seem too strong, or

even arbitrary. Could it not be the case that the ancilla space $\mathcal{H}_A$ carries a non-trivial representation $U_A(g)$, and could one not require invariance of ρ_{SA} under $(U_S(g) \otimes U(g)_A) \bullet \left(U_S^\dagger(g) \otimes U_A^\dagger(g)\right)$ instead?

The latter type of invariance occurs in the case of spacetime symmetries, for example translation invariance, where physics is expected to remain invariant under a global translation of every system. This is an instance of a global symmetry, which is typically understood to be distinct from a gauge symmetry. Global symmetries act as $\big(U_S(g) \otimes U_A(g)\big) \bullet \big(U_S^\dagger(g) \otimes U_A^\dagger(g)\big)$, and they map between physically equivalent descriptions of the quantum state under a lack of classical external reference frame for G in the quantum information approach [40]. As can be seen immediately by tracing over A, invariance of a purification $|\psi\rangle_{SA}$ of ρ_S under this symmetry implies that ρ_S has weak symmetry under $U(g)$, i.e. $U_S(g)\rho_S U_S^\dagger(g) = \rho_S$. In Theorem 2 (see also [39]) we show the converse direction: for any invariant state ρ_S one can find an ancilla $\mathcal{H}_A$ and purification $|\Psi\rangle_{SA}$ such that $|\Psi\rangle_{SA}$ is invariant under $U_S(g) \otimes U_A(g)$. Thus, the requirement of weak symmetry of a state ρ_S can be seen to follow from the existence of a purification which is invariant under a global symmetry.

A gauge symmetry is local if it acts non-trivially only on the system of interest, like e.g. the U(1) gauge symmetry of quantum electrodynamics. In the case of local gauge invariance, one expects invariance of the global quantum state under $\big(U_S(g) \otimes \mathbb{I}_A\big) \bullet \big(U_S^\dagger(g) \otimes \mathbb{I}_A\big)$. Permutation symmetry is a symmetry that acts locally on a system S of N particles. Thus the requirement of complete invariance under permutations follows from the understanding of permutation invariance as a gauge symmetry and not a global symmetry.

M. Global states in various approaches to quantum references frames

There are a number of different frameworks for the study of quantum reference frames, including the perspective-neutral framework [23–26], the operational framework [41–47], and the quantum information-theoretic approach [40, 48–52], amongst others. In all these approaches there exists a representation $U(g)$ of the symmetry group G of interest. In the perspective-neutral approach, the global state is assumed to transform under a trivial representation of G, i.e. $U(g)|\psi\rangle = |\psi\rangle$, whereas in the operational and the quantum information-theoretic frameworks, the global state is assumed to be weakly symmetric under $U(g)$, i.e. $U(g)\rho U^\dagger(g) = \rho$.

The requirement of transforming trivially under the representation $U(g)$ is in fact equivalent to being strongly symmetric as we now show. Any state which is strongly symmetric and transforms under $U(g)$ as $e^{i\theta(g)}$ transforms trivially under the projectively equivalent representation $e^{-i\theta(g)}U(g)$. Hence requiring strong symmetry under $U(g)$ is equivalent to requiring that the states transform trivially under some representation $V(g)$ which is projectively equivalent to $U(g)$.

Any non-invariant state ρ can be mapped to a weakly symmetric state $\tilde{\rho}$ which gives the same probabilities on all invariant observables via the incoherent, or weak, twirl: $\tilde{\rho} = \int_{g\in G} U(g)\rho U^\dagger(g) dg$. A non-invariant state ρ can be mapped to a strongly symmetric state ρ_{phys} which gives the same probabilities on all observables with full support on the trivial subspace via the coherent, or strong, twirl: $\rho_{\text{phys}} = \int_{g\in G}\int_{g'\in G} U(g)\rho U^\dagger(g') dg dg'$. The action of the coherent twirl on pure states is $|\psi\rangle \mapsto \int_g U(g)|\psi\rangle dg$.

Given a physical scenario with a symmetry G, imposing the additional requirement of complete invariance (or not) allows one to single out the perspective-neutral framework (or not) as the relevant approach. As discussed above, this requirement could be motivated by an understanding of $U(g)$ as a gauge symmetry, or by appealing to the church of the larger Hilbert space. An explicit argument showing that the coherent twirl, but not the incoherent twirl, fulfills the requirements needed to define a gauge theory can be found in [53, Section II. B. 3]. In contrast, the incoherent twirl is defended in [54] by appeal to Bayesian probability.

The generalization of Theorems 2 and 3 in the main text beyond the quantum permutation group, showing how invariance under quantum permutations gives rise to the perspective-neutral subspace, will be given in upcoming work [28].

REFERENCES

[1] F. Murnaghan. Representations of locally compact groups. `https://www.math.toronto.edu/murnaghan/courses/mat1196/`, 2013.

[2] A. Robert. *Introduction to the Representation Theory of Compact and Locally Compact Groups.* Cambridge University Press, Cambridge, 1983.

[3] B. Simon. *Representations of Finite and Compact Groups*, volume 10 of *Graduate Studies in Mathematics*. American Mathematical Society, Providence, RI, 1996.

[4] A. Peres. *Quantum Theory: Concepts and Methods.* Kluwer Academic Publishers, New York, 2002.

[5] J. B. Hartle and J. R. Taylor. Quantum mechanics of paraparticles. *Physical Review*, 178(5):2043–2051, 1969. doi:10.1103/PhysRev.178.2043.

[6] R. H. Stolt and J. R. Taylor. Classification of paraparticles. *Physical Review D*, 1(8):2226–2228, 1970. doi:10.1103/PhysRevD.1.2226.

[7] A B Zamolodchikov and V A Fateev. Nonlocal (parafermion) currents in two-dimensional conformal quantum field theory and self-dual critical points in z/sub n/-symmetric statistical systems. *Sov. Phys. - JETP (Engl. Transl.); (United States)*, 62:2, 08 1985. ISSN ISSN SPHJA. URL `https://www.osti.gov/biblio/5929972`.

[8] Paul Fendley. Free parafermions. *Journal of Physics A: Mathematical and Theoretical*, 47(7):075001, January 2014. ISSN 1751-8121. doi:10.1088/1751-

8113/47/7/075001. URL http://dx.doi.org/10.1088/1751-8113/47/7/075001.
[9] H. S. Green. A generalized method of field quantization. *Physical Review*, 90:270–273, 1953. doi:10.1103/PhysRev.90.270.
[10] A. M. L. Messiah and O. W. Greenberg. Symmetrization postulate and its experimental foundation. *Physical Review*, 136:B248–B267, 1964. doi:10.1103/PhysRev.136.B248.
[11] I. Bialynicki-Birula. Elementary particles and generalized statistics. *Nuclear Physics*, 49:605–608, 1963. doi:10.1016/0029-5582(63)90124-X.
[12] Y. Ohnuki and S. Kamefuchi. Wavefunctions of identical particles. *Annals of Physics*, 51:337–358, 1969. doi:10.1016/0003-4916(69)90217-6.
[13] P. V. Landshoff and H. P. Stapp. Parastatistics and a unified theory of identical particles. *Annals of Physics*, 43:72–92, 1967. doi:10.1016/0003-4916(67)90317-X.
[14] C. F. Roos, A. Alberti, D. Meschede, P. Hauke, and H. Häffner. Revealing quantum statistics with a pair of distant atoms. *Physical Review Letters*, 119:160401, 2017. doi:10.1103/PhysRevLett.119.160401.
[15] N. P. Landsman. Quantization and superselection sectors III: Multiply connected spaces and indistinguishable particles. *Rev. Math. Phys.*, 28(09):1650019, 2016. doi:10.1142/S0129055X16500197.
[16] R. H. Stolt and J. R. Taylor. Correspondence between the first- and second-quantized theories of paraparticles. *Nuclear Physics B*, 19(1):1–19, 1970. doi:10.1016/0550-3213(70)90024-6.
[17] F. Giacomini, E. Castro-Ruiz, and Č. Brukner. Quantum mechanics and the covariance of physical laws in quantum reference frames. *Nature Communications*, 10:494, 2019. doi:10.1038/s41467-018-08155-0.
[18] M. Krumm, P. A. Höhn, and M. P. Müller. Quantum reference frame transformations as symmetries and the paradox of the third particle. *Quantum*, 5:530, 2021. doi:10.22331/q-2021-08-27-530.
[19] P. A. Höhn, M. Krumm, and M. P. Müller. Internal quantum reference frames for finite abelian groups. *Journal of Mathematical Physics*, 63:112207, 2022. doi:10.1063/5.0088485.
[20] A. de la Hamette and T. D. Galley. Quantum reference frames for general symmetry groups. *Quantum*, 4:367, 2020. doi:10.22331/q-2020-11-30-367.
[21] C. Cepollaro, A. Akil, P. Cieśliński, A.-C. de la Hamette, and Č. Brukner. The sum of entanglement and subsystem coherence is invariant under quantum reference frame transformations, 2024. URL https://arxiv.org/abs/2406.19448. arXiv:2406.19448 [quant-ph].
[22] J. Butterfield. A philosophical look at quantum reference frames. Talk at the Symposium for Daniel Greenberger's 90th birthday, Vienna, 2023.
[23] P. A. Höhn. Switching internal times and a new perspective on the 'wave function of the universe'. *Universe*, 5:116, 2019. doi:10.3390/universe5050116.
[24] A. Vanrietvelde, P. A. Höhn, F. Giacomini, and E. Castro-Ruiz. A change of perspective: switching quantum reference frames via a perspective-neutral framework. *Quantum*, 4:225, 2020. doi:10.22331/q-2020-01-27-225.
[25] A. de la Hamette, T. D. Galley, P. A. Höhn, L. Loveridge, and M. P. Müller. Perspective-neutral approach to quantum frame covariance for general symmetry groups, 2021. URL https://arxiv.org/abs/2110.13824. arXiv:2110.13824 [quant-ph].
[26] A. Vanrietvelde, P. A. Höhn, and F. Giacomini. Switching quantum reference frames in the n-body problem and the absence of global relational perspectives. *Quantum*, 7:1088, 2023. doi:10.22331/q-2023-08-22-1088.
[27] M. Mekonnen, S. L. Ludescher, and M. P. Müller. Distinguishing bosons and fermions with internal quantum reference frames. in preparation, 2025.
[28] T. D. Galley, S. L. Ludescher, M. Mekonnen, and M. P. Müller. Generalizing the perspective-neutral framework to quantum reference frames that are not subsystems. in preparation, 2025.
[29] A.-C. de la Hamette, V. Kabel, E. Castro-Ruiz, and Č. Brukner. Quantum reference frames for an indefinite metric. *Communications Physics*, 6:231, 2023. doi:10.1038/s42005-023-01344-4.
[30] L. Hardy. Implementation of the quantum equivalence principle. In F. Finster, D. Giulini, J. Kleiner, and J. Tolksdorf, editors, *Progress and Visions in Quantum Theory in View of Gravity*. Birkhäuser, Cham, 2020.
[31] F. Giacomini and Č. Brukner. Einstein's equivalence principle for superpositions of gravitational fields and quantum reference frames, 2020. URL https://arxiv.org/abs/2012.13754. arXiv:2012.13754 [quant-ph].
[32] F. Giacomini and Č. Brukner. Quantum superposition of spacetimes obeys einstein's equivalence principle. *AVS Quantum Science*, 4:015601, 2022. doi:10.1116/5.0070018.
[33] V. Kabel, A.-C. de la Hamette, E. Castro-Ruiz, and Č. Brukner. Quantum conformal symmetries for spacetimes in superposition. *Quantum*, 8:1547, 2024. doi:10.22331/q-2024-12-04-1547.
[34] M. Nauenberg. Einstein's equivalence principle in quantum mechanics revisited. *American Journal of Physics*, 84:879–882, 2016. doi:10.1119/1.4962981.
[35] C. Marletto and V. Vedral. On the testability of the equivalence principle as a gauge principle detecting the gravitational t^3 phase. *Frontiers in Physics*, 8:176, 2020. doi:10.3389/fphy.2020.00176.
[36] O. Dobkowski, B. Trok, P. Skakunenko, Y. Japha, D. Groswasser, M. Efremov, C. Marletto, I. Fuentes, R. Penrose, V. Vedral, W. P. Schleich, and R. Folman. Observation of the quantum equivalence principle for matter-waves, 2025. URL https://arxiv.org/abs/2502.14535. arXiv:2502.14535 [quant-ph].
[37] P. Sala, S. Gopalakrishnan, M. Oshikawa, and Y. You. Spontaneous strong symmetry breaking in open systems: Purification perspective, 2024. URL https://arxiv.org/abs/2405.02402. arXiv:2405.02402 [cond-mat.stat-mech].
[38] L. A. Lessa, R. Ma, J.-H. Zhang, Z. Bi, M. Cheng, and C. Wang. Strong-to-weak spontaneous symmetry breaking in mixed quantum states, 2024. URL https://arxiv.org/abs/2405.03639. arXiv:2405.03639 [cond-mat.stat-mech].
[39] R. Ma and C. Wang. Average symmetry-protected topological phases. *Phys. Rev. X*, 13:031016, Aug 2023. doi:10.1103/PhysRevX.13.031016.
[40] S. D. Bartlett, T. Rudolph, and R. W. Spekkens. Reference frames, superselection rules, and quantum information. *Reviews of Modern Physics*, 79(2):555–609, 2007. doi:10.1103/RevModPhys.79.555.
[41] T. Miyadera, L. Loveridge, and P. Busch. Approxi-

mating relational observables by absolute quantities: a quantum accuracy-size trade-off. *Journal of Physics A: Mathematical and Theoretical*, 49(18):185301, 2016. doi: 10.1088/1751-8113/49/18/185301.

[42] L. Loveridge, P. Busch, and T. Miyadera. Relativity of quantum states and observables. *EPL (Europhysics Letters)*, 117:40004, 2017. doi:10.1209/0295-5075/117/40004.

[43] L. Loveridge, T. Miyadera, and P. Busch. Symmetry, reference frames, and relational quantities in quantum mechanics. *Foundations of Physics*, 48:135–198, 2018. doi:10.1007/s10701-018-0138-3.

[44] L. Loveridge. A relational perspective on the wigner-araki-yanase theorem. In *Journal of Physics: Conference Series*, volume 1638, page 012009, 2020. doi: 10.1088/1742-6596/1638/1/012009.

[45] J. Głowacki. *Operational Quantum Frames: An operational approach to quantum reference frames*. PhD thesis, Polish Academy of Sciences, Center for Theoretical Physics, 2023.

[46] T. Carette, J. Głowacki, and L. Loveridge. Operational quantum reference frame transformations, 2023. URL https://arxiv.org/abs/2303.14002. arXiv:2303.14002 [quant-ph].

[47] J. Głowacki. Towards relational quantum field theory, 2024. URL https://arxiv.org/abs/2405.15455. arXiv:2405.15455 [quant-ph].

[48] A. Kitaev, D. Mayers, and J. Preskill. Superselection rules and quantum protocols. *Physical Review A*, 69(5): 052326, 2004. doi:10.1103/PhysRevA.69.052326.

[49] G. Gour and R. W. Spekkens. The resource theory of quantum reference frames: manipulations and monotones. *New Journal of Physics*, 10:033023, 2008. doi: 10.1088/1367-2630/10/3/033023.

[50] G. Gour, I. Marvian, and R. W. Spekkens. Measuring the quality of a quantum reference frame: The relative entropy of frameness. *Physical Review A*, 80:012307, 2009. doi:10.1103/PhysRevA.80.012307.

[51] I. Marvian. *Symmetry, Asymmetry and Quantum Information*. PhD thesis, University of Waterloo, 2012.

[52] I. Marvian and R. W. Spekkens. Modes of asymmetry: The application of harmonic analysis to symmetric quantum dynamics and quantum reference frames. *Physical Review A*, 90:062110, 2014. doi: 10.1103/PhysRevA.90.062110.

[53] P. A. Höhn, I. Kotecha, and F. M. Mele. Quantum frame relativity of subsystems, correlations and thermodynamics, 2023. URL https://arxiv.org/abs/2308.09131. arXiv:2308.09131 [quant-ph].

[54] D. Poulin. Toy model for a relational formulation of quantum theory. *International Journal of Theoretical Physics*, 45:1189, 2006. doi:10.1007/s10773-006-9052-0.
