# Supplementary material for: Invariance under quantum permutations rules out parastatistics
Source: Nat Commun. 2026 May 28;17:6947. doi: 10.1038/s41467-026-73064-6 (PMC13389154; doi:10.1038/s41467-026-73064-6)
Supplement: Supplementary file 2 — Transparent Peer Review file [file 41467_2026_73064_MOESM2_ESM.pdf]

nature portfolio

Peer Review File

Invariance under quantum permutations rules out parastatistics

Corresponding Author: Dr Markus Müller

This file contains all reviewer reports in order by version, followed by all author rebuttals in order by version.

Attachments originally included by the reviewers as part of their assessment can be found at the end of this file.

Version 0:

Reviewer comments:

Reviewer #1

(Remarks to the Author)
The manuscript "Invariance under quantum permutations rules out parastatistics" addresses the longstanding question of why all known fundamental particles are either bosons or fermions, despite the fact that quantum theory formally allows more general behaviors of identical particles under permutations.

The authors work in a general setting with a unitary representation of the permutation group S_N on the N-particle Hilbert space (in first or second quantization), allowing for Green-type parastatistics described by higher-dimensional irreps of S_N. They then introduce two strengthened notions of permutation invariance:
1. Complete invariance: not only the reduced state of the particle system S must be invariant under permutations, but all extensions and purifications ρ_{SA} must be invariant under local permutations on S.
2. Invariance under quantum permutations: permutations that are applied coherently and conditionally on the outcomes of permutation-invariant observables ("branch-wise permutations"), motivated by the framework of quantum reference frames.

They prove that complete invariance is satisfied if and only if the state is fully supported in the bosonic or fermionic subspace. Under invariance with respect to suitable quantum permutation groups, paraparticle sectors are forced to be effectively classical (diagonal in a fixed basis); they cannot become entangled with ancillas or participate in nontrivial reversible interactions, and under a stronger form of quantum permutation invariance they must be maximally mixed and uncorrelated. The results provide a conceptually appealing, information-theoretic explanation for the empirical absence of Green-type paraparticles among fundamental particles, and connect ideas from quantum information, representation theory, and internal quantum reference frames. Nevertheless, I do have some critical comments and concerns discussed below.

A. Major comments

1.Relation to anyons and braid-group statistics
The manuscript focuses on parastatistics understood as irreducible representations of the permutation group S_N for systems of indistinguishable particles. This is a valid scope, but many readers will associate "statistics beyond bosons and fermions" primarily with anyons and braid-group statistics in two-dimensional systems (fractional quantum Hall quasiparticles, Majorana zero modes, parafermionic zero modes, etc.).

In the present work, the existence of a unitary representation π ↦ U(π) of the symmetric group S_N on the N-particle Hilbert space is assumed from the outset. This is appropriate for point-like particles in 3+1 dimensions, where the fundamental group of the configuration space is S_N, but it fails for anyons in 2+1 dimensions, where exchanges are described by the braid group B_N and the elementary exchanges do not satisfy σ_i^2 = I in general. Hence, the authors' results do not constrain anyon statistics or braid-group representations, which are outside the assumed framework.

Given the prominence of anyons in the literature on generalized statistics, it would be very helpful if the authors explicitly clarified in the introduction that their results address Green-type parastatistics for S_N, and that anyonic quasiparticles in 2D topological phases (FQHE, Majorana and parafermionic zero modes, etc.) fall outside the scope of the present analysis.

2.Fundamental vs. emergent particles
In the final section, the authors briefly note that their results do not conflict with recent proposals for parastatistical correlations in many-body or quasiparticle systems, and attribute the resolution of this apparent tension to the distinction between fundamental and emergent particles. This is an important point, particularly in light of fractional quantum Hall anyons and topological quasiparticles more broadly.

I would encourage the authors to expand this discussion. In particular, it would be good to:
- Provide explicit examples of emergent excitations (e.g., anyons in FQHE, Majorana zero modes, Z_n parafermionic modes) that the authors consider to be outside the scope of their arguments;
- Clarify that their invariance principles are intended as postulates for truly fundamental degrees of freedom (electrons, quarks, photons, etc.), and need not hold for effective low-energy quasiparticles in engineered many-body systems;
- Comment briefly on how one might expect the assumptions of complete or quantum permutation invariance to fail in such emergent systems.

This would make it clearer why the strong conclusions about the absence of paraparticles do not undermine the rich physics of anyons and other emergent excitations in condensed-matter systems.

3.Status and physical motivation of complete invariance
Complete invariance is a strong requirement: the authors demand that every extension and, in particular, every purification of the local state ρ_S is invariant under local permutations on S. This is reminiscent of the “church of the larger Hilbert space” attitude in quantum information theory and some versions of the many-worlds interpretation, but it is not an inevitable consequence of the basic formalism.

It would be useful if the authors could discuss a bit more the physical motivation and status of this assumption. For instance:
- Is complete invariance expected to hold in realistic quantum field theories with gauge symmetries and superselection sectors?
- Are there models where standard permutation invariance holds for S but fails once one considers arbitrary ancillas and purifications, and how would such models be interpreted physically?
- To what extent is complete invariance meant as a fundamental principle (analogous to complete positivity of channels) versus a convenient strengthening that singles out bosons and fermions?

Clarifying these points would make the scope and significance of Theorem 1 more transparent, especially for readers who are less invested in the “pure-state universe” perspective.

4. Quantum permutations as quantum reference frame transformations
The interpretation of quantum permutations as quantum reference frame (QRF) transformations is conceptually attractive and aligns well with the recent literature. At the same time, it highlights an important subtlety: whether a given transformation is a QRF symmetry operation depends on the type of system.

For bosons and fermions, the authors argue that elements of the appropriate quantum permutation group (V_+ for bosons, V_- for fermions) act as relabelings and should not change physical predictions. For systems where exchanging excitations is itself a physical operation used to implement gates—most prominently non-Abelian anyons—these transformations are not QRF changes but actual dynamical operations.

It would be helpful if the authors could make this distinction explicit in the main text: quantum permutations are QRF transformations only for those particle types for which they preserve all physical predictions; for systems like anyons, the corresponding exchange operations lie outside the symmetry group assumed in this work. This again underlines that the results are about fundamental permutation statistics, not about anyonic braiding.

B. Minor comments

- In the introduction, it may help to briefly recall that in D ≥ 3 the configuration space of N identical point particles has fundamental group S_N, whereas in 2D it has braid group B_N; this would naturally separate the present focus on S_N-based parastatistics from the anyonic case.

- Figures 1 and 2 are clear and useful. It might be worth explicitly stating in the caption of Fig. 2 that the control observable used to define branches must be permutation-invariant.

- Tables I and II in the Methods section are very helpful summaries of the allowed invariant states and correlations. It could be useful to refer to these tables explicitly in the main text when first stating Theorems 1–3.

- Since “parafermions” also appear in the condensed-matter literature to denote certain anyonic zero modes rather than Green-type parastatistical fields, a short remark clarifying the terminology would help avoid confusion.

C. Conclusion
The manuscript is clearly written, technically sound as far as I can see, and offers a novel, information-theoretic perspective on the absence of fundamental paraparticles. Subject to additions and corrections as elaborated above, I would be inclined to positively reconsider it for publication in Nature Communications. Eventually, the paper could make a valuable contribution to on-going discussions revolving around quantum statistics and/or quantum reference frames.

Reviewer #2

(Remarks to the Author)
See the attached file.

Reviewer #3

(Remarks to the Author)
The topic of the paper is the long-standing fundamental question as to why systems of identical particles observed in Nature are always either symmetric (Bosons) or antisymmetric (Fermions) with respect to permutations of particles. The question arises from the mathematical fact that the representation theory of the permutation group allows multiple irreducible representations, only two of which correspond to Bosonic and Fermionic particles, the rest being called "para-particles".

While existing work on the problem is mainly done in the context of quantum field theory, the present authors propose another approach, which is rather based on an operational approach typical for quantum information theory, and does not require field-theoretic machinery. The authors consider two ways of formulating permutation invariance: (1) the invariance of all results of measurements made on an open quantum system, and (2) invariance of measurement results conditioned on the outcome of a projective measurement. They then proceed to show how either of these assumptions can be used to rule out para-particles. This is the noteworthy result of the paper. Finally the authors link the results to recent research on quantum reference frames.

The authors' approach seems entirely original, and very appealing from the perspective of operational quantum physics, which (unlike much of field theory) is solidly grounded on the theory of prepare-and-measure type experiments. In particular, the formulation of "quantum permutations" is quite neat as it utilises the fundamental concept of quantum superposition and the associated conditional branching based on measurement outcomes. The work is clearly significant for a broad area of fundamental physics which seeks to understand how the structure of the matter can be understood in the micro-level. The mathematical work is highly nontrivial: the authors study in detail the representation theory of the symmetric group, and show rigorously how the para-particle representations are ruled out by the assumptions. The proofs are given in good detail in the Methods section, with some parts postponed to the supplemental material.

Based on this, I will recommend that the paper be published, after the authors consider the following minor points:

1) On page 2, the authors explain the need to formulate particle permutations in terms of modes rather than tensor factors when working in the second quantisation (Fock space). I understand this, but the following justification seems slightly confusing: "In second quantization, there are no tensor factors to permute [...]". Now, a common way of introducing the Fock space is done by arranging all N-particle spaces \mathcal H^{\otimes N} into a direct sum, and defining e.g. annihilation operator first on the whole of this space and then projecting to the symmetric (antisymmetric) subspaces to get the usual CCR (CAR) relations. So here we certainly have tensor factors, while the modes only arise after we choose a basis for \mathcal H. So one might naively think of defining the standard permutations for each N-particle space separately (as in the first quantisation) to get a description for the total Fock space (2nd quantisation). Perhaps the authors could add a brief explanation as to why this does not work.

2) In the methods section, the proof of Theorem 1 is structured somewhat oddly. In fact, after Lemma 1 the authors state that Thm 1 follows from this Lemma, but the claim of Thm 1 (state being supported in either Bosonic or Fermionic subspace) is not explicitly mentioned in the statement of the Lemma 1 but rather appears as a side consequence in the middle of its proof. Perhaps the authors could improve the structure here.

Version 1:

Reviewer comments:

Reviewer #1

(Remarks to the Author)
I believe that the revised manuscript is stronger than the original version and addresses most of the earlier concerns. The central idea remains interesting and suitable for publication. Before acceptance, I'd like to encourage the authors to address the following points:
1. The following sentence in the Abstract “we give two independent arguments that rule out parastatistics universally” may give a wrong impression because the authors also write: “They do not apply to other important families of particles with exotic exchange statistics such as anyons, which transform under the braid group, and (as we will discuss) they neither rule out emergent parastatistics nor paraparticles where the invariant permutation action does more than just swapping modes, as in graded systems described by Lie superalgebras”.
2. The reply seems to be appropriately careful in describing complete invariance as a well-motivated additional postulate, rather than a strict necessity. However, the manuscript seems to phrase the step from ordinary invariance to complete invariance too strongly, e.g. by stating that “The assumption that “physics is invariant” under local permutations of S should then imply the condition...”. That wording makes the argument sound more compulsory than claimed in the reply letter.

Reviewer #2

(Remarks to the Author)
In my opinion, in the revised version the authors successfully answered questions and objections raised by the referees. They presented a series of arguments and counterarguments which go beyond what is necessary and even expected. The strong point of the paper is that it forces to sharpen the notion of permutation invariance of paraparticles. As mentioned in my previous report, this work can have significant implications for the area. I recommend the revised version to be published as is.

Reviewer #3

(Remarks to the Author)
The authors have addressed my minor comments, explained the changes carefully, and amended the paper accordingly. I am therefore happy to recommend it for publication based on the assessment in my previous report.

Open Access This Peer Review File is licensed under a Creative Commons Attribution 4.0 International License, which permits use, sharing, adaptation, distribution and reproduction in any medium or format, as long as you give appropriate credit to the original author(s) and the source, provide a link to the Creative Commons license, and indicate if changes were made.
In cases where reviewers are anonymous, credit should be given to 'Anonymous Referee' and the source.
The images or other third party material in this Peer Review File are included in the article’s Creative Commons license, unless indicated otherwise in a credit line to the material. If material is not included in the article’s Creative Commons license and your intended use is not permitted by statutory regulation or exceeds the permitted use, you will need to obtain permission directly from the copyright holder.
To view a copy of this license, visit https://creativecommons.org/licenses/by/4.0/

Invariance under quantum permutations rules out parastatistics

Dear Reviewers,

we are very grateful for your detailed and thoughtful comments. Indeed, we are very happy about the high quality of the reports that we have received and think that they have allowed us to improve the quality of the paper significantly.

Before answering all comments one by one below, let us briefly describe the most important change that we have made to the paper: As requested, we have significantly **clarified the scope of our results**, which rule out parastatistical systems transforming under higher-dimensional representations of the symmetric group. We have

- explained **why they do not apply to anyons** (because these transform under a different group) **or to effective / emergent particles** (because further external degrees of freedom will typically transform under the group). In addition to the remarks in the main text, a new example calculation in the Supplementary Material S3.A shows this in detail.
- We also explain now **in what sense our results apply to graded systems described by superalgebras**: they correctly predict that physical states are invariant under permutations up to phase, but they do not prevent the appearance of exotic exchange statistics because of the underlying nonstandard representation of the symmetric group.

Further changes include clarifying the relation to realistic quantum field theory, the interpretation of complete invariance, and the non-existence of tensor factor permutations in second quantization. These and all other changes are described in detail in the point-by-point responses below. We are also resubmitting a version of our manuscript and the supplementary material with all changes marked in blue color.

We hope that we have managed to respond successfully to all your comments and requests. We sincerely thank you again for your time and effort in reviewing our manuscript.

Yours sincerely,
Manuel Mekonnen, Thomas D. Galley, and Markus P. Müller

I. REVIEW REPORT 1

Below, we respond to each of the comments individually. (Reviewer comments in green, answers in black.)

1. Relation to anyons and braid-group statistics

The manuscript focuses on parastatistics understood as irreducible representations of the permutation group S_N for systems of indistinguishable particles. This is a valid scope, but many readers will associate "statistics beyond bosons and fermions" primarily with anyons and braid-group statistics in two-dimensional systems (fractional quantum Hall quasiparticles, Majorana zero modes, parafermionic zero modes, etc.).

In the present work, the existence of a unitary representation $\pi \mapsto U(\pi)$ of the symmetric group S_N on the N-particle Hilbert space is assumed from the outset. This is appropriate for point-like particles in $3+1$ dimensions, where the fundamental group of the configuration space is S_N, but it

fails for anyons in $2+1$ dimensions, where exchanges are described by the braid group B_N and the elementary exchanges do not satisfy $\sigma_i^2 = I$ in general. Hence, the authors' results do not constrain anyon statistics or braid-group representations, which are outside the assumed framework.

Given the prominence of anyons in the literature on generalized statistics, it would be very helpful if the authors explicitly clarified in the introduction that their results address Green-type parastatistics for S_N, and that anyonic quasiparticles in 2D topological phases (FQHE, Majorana and parafermionic zero modes, etc.) fall outside the scope of the present analysis.

We agree that it is important to clarify the scope of our arguments early on. Hence, we have added the following paragraph to the introduction:

Our results apply to paraparticles defined as fundamental indistinguishable particles transforming under multidimensional irreducible representations of the symmetric group. They do not apply to other important families of particles with exotic exchange statistics such as anyons, which transform under the braid group, and (as we will discuss) they neither rule out emergent parastatistics nor paraparticles where the invariant permutation action does more than just swapping modes, as in graded systems described by Lie superalgebras [Toppan]. On the other hand, our results are rather general because they apply to all kinds of quantum systems that can be permuted, not only to particles with spatiotemporal locations. To this end, our approach defines permutation invariance directly at the level of Hilbert space operators, and not on the level of classical configuration space [Landsman].

Let us briefly clarify this distinction. In *Quantization and superselection sectors iii: Multiply connected spaces and indistinguishable particles* [1], Landsmann distinguishes two main theoretical approaches to permutation invariance: the operator approach (of Messiah and Greenberg) and the configuration space approach (of Souriau, Laidlaw, and DeWitt-Morette).

The operator approach is the one adopted in the present work, where permutation-invariance is directly defined in terms of of Hilbert spaces, without appeal to configuration space. Here the symmetry group for N Hilbert spaces is always S_N. In this context, a permutation of two particles is not necessarily given by two trajectories in configuration space.

In contrast, the configuration space approach does begin with the classical space $\mathbb{R}^d$ and then defines the configuration space of permutation invariant states as $((\mathbb{R}^d)^N \setminus \Delta_N)/S_N$, where Δ_N is given by the points $(q_1, ..., q_N) \in (\mathbb{R}^d)^N$ such at least one pair of $q_i = q_j$, $i \neq j$. In this case one has that the homotopy group $\pi_1((\mathbb{R}^d)^N)$ of $(\mathbb{R}^d)^N$ is S_N for $d > 2$ but B_N for $d = 2$.

Therefore these two different approaches to defining indistinguishable particles agree for $d > 2$ but disagree for $d = 2$. They also disagree in their scope: the Hilbert space operator approach also applies to situations where the permuted quantum systems do not have spatial locations (for example, they might be logical qubits in a quantum computer). We hope that the additional paragraph clarifies this.

2. Fundamental vs. emergent particles

In the final section, the authors briefly note that their results do not conflict with recent proposals for parastatistical correlations in many-body or quasiparticle systems, and attribute the resolution of this apparent tension to the distinction between fundamental and emergent particles. This is an important point, particularly in light of fractional quantum Hall anyons and topological quasiparticles more broadly.

I would encourage the authors to expand this discussion. In particular, it would be good to: - Provide explicit examples of emergent excitations (e.g., anyons in FQHE, Majorana zero modes, Z_n parafermionic modes) that the authors consider to be outside the scope of their arguments; - Clarify that their invariance principles are intended as postulates for truly fundamental degrees of freedom (electrons, quarks, photons, etc.), and need not hold for effective low-energy quasiparticles

in engineered many-body systems; - Comment briefly on how one might expect the assumptions of complete or quantum permutation invariance to fail in such emergent systems.

This would make it clearer why the strong conclusions about the absence of paraparticles do not undermine the rich physics of anyons and other emergent excitations in condensed-matter systems.

This is indeed an important consideration and we agree that it requires further exploration. We have worked out an explicit example, which we have now included in Subsection S2.C of the Supplementary Material, which gives an intuitive explanation for why complete invariance will generically fail in emergent or paraparticle systems S: because the fundamental, truly "physics-preserving" transformations $U(\pi)$ will not only act on S, but will also non-trivially act on other systems A that we have deliberately ignored to define the effective parasystem.

We follow an example from Landsman [1, Section 9] of parafermions emerging from three Bosonic isospin doublets, using a more explicitly group-theoretic characterisation. This is an example of emergent paraparticles from Bosons only in an abstract sense, namely there is no explicit mechanism leading to this emergence just a posited restriction on states and observables, however the simplicity of the example allows for clarity.

Example 1. *We begin with three distinguishable isospin doublets* $\mathcal{H}^{(3)} = (L^2(\mathbb{R}^3) \otimes \mathbb{C}^2)^{\otimes 3}$*, which carry the following representation of* S_3*:*

$$U^{(3)}(\pi)\psi_{a_1,a_2,a_3}(q_1, q_2, q_3) = \psi_{a_{\pi(1)},a_{\pi(2)},a_{\pi(2)}}(q_{\pi(1)}, q_{\pi(2)}, q_{\pi(3)}). \tag{1}$$

We consider the subspace $\mathcal{H}^{(3)}_{\lambda_0}$ *of Bosonic isospin doublets spanned by symmetrized wavefunctions:*

$$\psi_{a_1,a_2,a_3}(q_1, q_2, q_3) = \psi_{a_{\pi(1)},a_{\pi(2)},a_{\pi(2)}}(q_{\pi(1)}, q_{\pi(2)}, q_{\pi(3)}). \tag{2}$$

Using Schur-Weyl for the action of S_3 *given in Equation* (1) *(i.e. permuting both spatial and spin labels) we have that the space* $\mathcal{H}^{(3)}$ *decomposes as:*

$$\mathcal{H}^{(3)} \simeq \bigoplus_{\lambda} V_\lambda \otimes W_\lambda \simeq \bigoplus_{\lambda} \mathcal{H}^{(3)}_\lambda, \tag{3}$$

where V_λ *carries the irreducible representation of* S_3 *labeled by* λ *and* W_λ *a representation of* $U(W_\lambda)$*. Since* W_λ *infinite-dimensional, one needs to choose the correct topology on* W_λ *to define* $U(W_\lambda)$*, as discussed in (Landsman) (c.f. footnote 7).*

However we can further decompose $\mathcal{H}^{(3)}_{\lambda_0}$ *by considering the fact that the spatial and spin components of* $\mathcal{H}^{(3)}$ *carry separate actions of* S_3*. We write* $\mathcal{H}^{(3)} \simeq \mathcal{H}^{(3),\text{spatial}} \otimes \mathcal{H}^{(3),\text{spin}}$ *where* $\mathcal{H}^{(3),\text{spatial}} \simeq L^2(\mathbb{R}^3)^{\otimes 3}$ *and* $\mathcal{H}^{(3),\text{spin}} \simeq (\mathbb{C}^2)^{\otimes 3}$*.* $\mathcal{H}^{(3),\text{spatial}}$ *carries a representation* $U^{(3),\text{spatial}}(\pi)$ *of* S_3 *(corresponding to permuting the spatial indices) and similarly* $\mathcal{H}^{(3),\text{spin}}$ *carries a representation* $U^{(3),\text{spin}}(\pi)$ *of* S_3 *(corresponding to permuting the spin indices).*

The representation $U^{(3)}$ *of* S_3 *given in Equation* (1) *corresponds to performing the same permutation* π *on both* $\mathcal{H}^{(3),\text{spatial}}$ *and* $\mathcal{H}^{(3),\text{spin}}$*, i.e.* $U^{(3)}(\pi) = U^{(3),\text{spatial}}(\pi) \otimes U^{(3),\text{spin}}(\pi)$*.*

Using Schur-Weyl we can first decompose the spaces $\mathcal{H}^{(3),\text{spatial}}$ *and* $\mathcal{H}^{(3),\text{spin}}$ *under their respec-*

tive S_3 actions:

$$\mathcal{H}^{(3),\text{spatial}} = L^2(\mathbb{R}^3)^{\otimes 3} \simeq \bigoplus_\lambda V_\lambda^{\text{spatial}} \otimes W_\lambda^{\text{spatial}} \simeq \bigoplus_\lambda \mathcal{H}_\lambda^{(3),\text{spatial}}, \tag{4}$$

$$\mathcal{H}^{(3),\text{spin}} = (\mathbb{C}^2)^{\otimes 3} \simeq \bigoplus_\lambda V_\lambda^{\text{spin}} \otimes W_\lambda^{\text{spin}} \simeq \bigoplus_\lambda \mathcal{H}_\lambda^{(3),\text{spin}}, \tag{5}$$

where $V_\lambda^{\text{spatial}}, V_\lambda^{\text{spin}}$ carry the irreducible representation of S_3 labeled by λ, $W_\lambda^{\text{spatial}}$ a representation of $U(W_\lambda^{\text{spatial}})$ and W_λ^{spin} an irreducible representation of $\mathrm{SU}(2)$.

Clearly for λ_0 the Bosonic representation, any state in $\mathcal{H}_{\lambda_0}^{(3),\text{spatial}} \otimes \mathcal{H}_{\lambda_0}^{(3),\text{spin}}$ transforms trivially under permutations and is therefore in $\mathcal{H}_{\lambda_0}^{(3)}$. This is just the statement that the symmetric subspace $\mathcal{H}_{\lambda_0}^{(3)}$ of $\mathcal{H}^{(3)} \simeq \mathcal{H}^{(3),\text{spatial}} \otimes \mathcal{H}^{(3),\text{spin}}$ contains the tensor product of the symmetric subspaces of the factors, namely $\mathcal{H}_{\lambda_0}^{(3),\text{spatial}} \otimes \mathcal{H}_{\lambda_0}^{(3),\text{spin}} \subset \mathcal{H}_{\lambda_0}^{(3)}$. However, as is well-known, the symmetric subspace of a composite system also contains the tensor product of the antisymmetric subspaces of the factors: $\mathcal{H}_{\lambda_1}^{(3),\text{spatial}} \otimes \mathcal{H}_{\lambda_1}^{(3),\text{spin}} \subset \mathcal{H}_{\lambda_0}^{(3)}$ where λ_1 labels the anti-symmetric subspace. Similarly for λ_2 there will be elements in $\mathcal{H}_{\lambda_2}^{(3),\text{spatial}} \otimes \mathcal{H}_{\lambda_2}^{(3),\text{spin}}$ which are invariant under $U_{\lambda_2}^{(3),\text{spatial}}(\pi) \otimes U_{\lambda_2}^{(3),\text{spin}}(\pi)$, i.e. which lie in $\mathcal{H}_{\lambda_0}^{(3)}$

For $\lambda_2 = (2,1)$ we can create a permutation-invariant state in $V_{\lambda_2}^{\text{spatial}} \otimes V_{\lambda_2}^{\text{spin}}$, where $V_{\lambda_2}^{\text{spatial}} \simeq V_{\lambda_2}^{\text{spin}} \simeq \mathbb{C}^2$ both transform under the $(2,1)$ irrep of S_3. From the well known fact that the singlet state is invariant under $U \otimes U$ we can define the following Bosonic state in $\mathcal{H}_{\lambda_0}^{(3)} \subset \mathcal{H}^{(3)}$:

$$|\Psi\rangle = \left(\frac{1}{\sqrt{2}}(|e_0\rangle_{V_{\lambda_2}^{\text{spatial}}} \otimes |e_1\rangle_{V_{\lambda_2}^{\text{spin}}} - |e_1\rangle_{V_{\lambda_2}^{\text{spatial}}} \otimes |e_0\rangle_{V_{\lambda_2}^{\text{spin}}})\right) \otimes |\psi\rangle_{W_{\lambda_2}^{\text{spatial}}, W_{\lambda_2}^{\text{spin}}} \tag{6}$$

where $\{|e_i\rangle_{V_{\lambda_2}^{\text{spatial}}}\}_{i=0,1}$ is a basis for $V_{\lambda_2}^{\text{spatial}}$ (and similarly for $V_{\lambda_2}^{\text{spin}}$) and $|\psi\rangle_{W_{\lambda_2}^{\text{spatial}}, W_{\lambda_2}^{\text{spin}}}$ an arbitrary state in $W_{\lambda_2}^{\text{spatial}}, W_{\lambda_2}^{\text{spin}}$. This is clearly invariant since a simultaneous permutation of the spatial and spin indices acts as $U_{V_{\lambda_2}^{\text{spatial}}}(\sigma) \otimes U_{V_{\lambda_2}^{\text{spin}}}(\sigma) \otimes \mathbb{1}_{W_{\lambda_2}^{\text{spatial}}} \otimes \mathbb{1}_{W_{\lambda_2}^{\text{spin}}}$ and the singlet state over the first two factors is invariant under any $U \otimes U$.

Let us ignore the isospin degrees of freedom and trace out $V_\lambda^{\text{spin}} \otimes W_\lambda^{\text{spin}}$:

$$\rho_{\text{para}} := \mathrm{Tr}_{V_{\lambda_2}^{\text{spin}} W_{\lambda_2}^{\text{spin}}} |\Psi\rangle\langle\Psi| = \frac{1}{2}\mathbf{1}_{V_{\lambda_2}^{\text{spatial}}} \otimes \rho_{W_{\lambda_2}^{\text{spatial}}} \tag{7}$$

where $\rho_{W_{\lambda_2}^{\text{spatial}}} = \mathrm{Tr}_{W_{\lambda_2}^{\text{spin}}}(|\psi\rangle\langle\psi|_{W_{\lambda_2}^{\text{spatial}}, W_{\lambda_2}^{\text{spin}}})$. This is indeed an invariant parafermionic state of three particles with just spatial degrees of freedom, since $\sum_i |e_i\rangle\langle e_i|_{V_\lambda^{\text{spatial}}}$ is the identity on the space $V_\lambda^{\text{spatial}}$ transforming under the $(2,1)$ irrep of S_3 and $V_\lambda^{\text{spatial}}$ transforms trivially under S_3

This was an example of a system of Bosonic particles which together with a restriction on the allowed states and measurements leads to a system of effective paraparticles. The effective paraparticles in the above example are permutation-invariant. But does it make sense to require complete invariance? The answer is in the negative, as we now show in the explanation below, which we also included in Subsection S3.A of the Supplementary Material.

Given an agent Alice using the effective paraparticles of Equation (7), an adversary Eve without restriction would have access to the purification given in Equation (6).

Alice can implement permutations only on the spatial components, and not the spin (since by construction of the effective paraparticles she does not have access to them). Her state ρ_{para} is

indeed invariant under $U_{V^{\text{spatial}}_{\lambda_2}}(\sigma)$ for all $\sigma \in S_3$.

The purification $|\Psi\rangle$ which Eve has access to is not invariant under $U_{V^{\text{spatial}}_{\lambda_2}}(\sigma) \otimes \mathbb{1}_{W^{\text{spatial}}_{\lambda_2}} \otimes \mathbb{1}_{V^{\text{spin}}_{\lambda_2}}(\sigma) \otimes \mathbb{1}_{W^{\text{spin}}_{\lambda_2}}$ and therefore does not obey complete invariance. Remember that the requirement of being a Bosonic state meant invariance under a different condition, namely $U_{V^{\text{spatial}}_{\lambda_2}}(\sigma) \otimes \mathbb{1}_{W^{\text{spatial}}_{\lambda_2}} \otimes U_{V^{\text{spin}}_{\lambda_2}}(\sigma) \otimes \mathbb{1}_{W^{\text{spin}}_{\lambda_2}}$.

We can see that the purification is not invariant under $U_{V^{\text{spatial}}_{\lambda_2}}(\sigma)$ from the following facts about the $(2,1)$ irrep of S_3, namely that for $\sigma = (123)$:

$$U(\sigma)\,|e_0\rangle = \omega\,|e_0\rangle\,, \tag{8}$$

$$U(\sigma)\,|e_1\rangle = \bar{\omega}\,|e_1\rangle\,, \tag{9}$$

where $\omega = e^{\frac{2\pi i}{3}}$. Therefore:

$$U_{V^{\text{spatial}}_{\lambda_2}}(\sigma) \otimes \mathbb{1}_{W^{\text{spatial}}_{\lambda_2}} \otimes \mathbb{1}_{V^{\text{spin}}_{\lambda_2}}(\sigma) \otimes \mathbb{1}_{W^{\text{spin}}_{\lambda_2}}\,|\Psi\rangle \tag{10}$$

$$=\Big(\frac{1}{\sqrt{2}}(\omega\,|e_0\rangle_{V^{\text{spatial}}_{\lambda_2}} \otimes |e_1\rangle_{V^{\text{spin}}_{\lambda_2}} - \bar{\omega}\,|e_1\rangle_{V^{\text{spatial}}_{\lambda_2}} \otimes |e_0\rangle_{V^{\text{spin}}_{\lambda_2}})\Big) \otimes |\psi\rangle_{W^{\text{spatial}}_{\lambda_2}, W_{\lambda_2^{\text{spin}}}} \tag{11}$$

$$\simeq\Big(\frac{1}{\sqrt{2}}(|e_0\rangle_{V^{\text{spatial}}_{\lambda_2}} \otimes |e_1\rangle_{V^{\text{spin}}_{\lambda_2}} - \omega\,|e_1\rangle_{V^{\text{spatial}}_{\lambda_2}} \otimes |e_0\rangle_{V^{\text{spin}}_{\lambda_2}})\Big) \otimes |\psi\rangle_{W^{\text{spatial}}_{\lambda_2}, W_{\lambda_2^{\text{spin}}}} \neq |\Psi\rangle \tag{12}$$

where $\simeq$ denotes equivalence up to global phase.

The above example shows that given an effective system of paraparticles emerging from an underlying Bosonic system, the assumption of complete invariance fails exactly because the full underlying Bosonic state is not invariant under permutations acting solely on the parastatistical subsystem.

3. Status and physical motivation of complete invariance

Complete invariance is a strong requirement: the authors demand that every extension and, in particular, every purification of the local state ρ_S is invariant under local permutations on S. This is reminiscent of the "church of the larger Hilbert space" attitude in quantum information theory and some versions of the many-worlds interpretation, but it is not an inevitable consequence of the basic formalism.

It would be useful if the authors could discuss a bit more the physical motivation and status of this assumption. For instance: - Is complete invariance expected to hold in realistic quantum field theories with gauge symmetries and superselection sectors? - Are there models where standard permutation invariance holds for S but fails once one considers arbitrary ancillas and purifications, and how would such models be interpreted physically? - To what extent is complete invariance meant as a fundamental principle (analogous to complete positivity of channels) versus a convenient strengthening that singles out bosons and fermions?

Clarifying these points would make the scope and significance of Theorem 1 more transparent, especially for readers who are less invested in the "pure-state universe" perspective.

Indeed, we expect complete invariance to apply more generally to gauge symmetries in realistic QFTs. We have added the following part at the end of the last paragraph in the "complete invariance" section to explain this:

Beyond permutations, it is natural to expect that complete invariance also holds for fundamental gauge symmetries in quantum field theory. In Subsection S3.B of the Supplementary Material,

we prove a generalization of Theorem 1: complete invariance under a compact group $\mathcal{G}$ is equivalent to the system S transforming under a one-dimensional representation of $\mathcal{G}$. Even though it is beyond our mathematical framework, note that Dirac quantization implements gauge symmetries via constraints such as $C|\psi\rangle = 0$ (for example, the Gupta-Bleuler condition $C = \partial^\mu A_\mu^+$ in quantum electrodynamics [Tong]). The gauge transformations G generated by C hence satisfy $G|\psi\rangle = |\psi\rangle$ (or, in more general Yang-Mills theories, $G|\psi\rangle = e^{i\theta}|\psi\rangle$ [Jackiw]), corresponding to one-dimensional representations, even if the gauge group is non-Abelian. Hence, if the mathematical framework can be suitably generalized, we expect complete invariance to hold here, confirming the natural intuition that it expresses "nothing in the world to change whatsoever" under gauge transformations.

In the answer to the referee's previous question, we have now also given an example where standard permutation invariance holds, but complete invariance fails. Moreover, this can be intuitively understood as the consequence of parastatistics of an *emergent, non-fundamental* system, coming from reducing the degrees of freedom that are allowed to be measured. We hope that this also contributes to the clarification of our concept of complete invariance.

Let us elaborate a bit further on the quantum information motivation of complete invariance, and its analogy with the QI notion of complete positivity. Consider the transpose operation, $T : \rho \mapsto \rho^\top$. This is a positive, but not completely positive operation. Now consider some quantum system S which is a qubit, and another qubit system A. Since T is not completely positive, (at least) one of the following two options must be true:

- Either T cannot be physically implemented on S;
- or Nature forbids any entangled states between system S and A.

Typically, we expect entanglement between S and A to be possible, and so we expect the first of the two options to apply. Complete positivity is then a condition that strengthens the positivity property to "play together nicely with system composition".

Similarly, consider a quantum system S in a state which is permutation-invariant, but not completely invariant, and A some other system. Then:

- Either the system S cannot be prepared in this state;
- or Nature forbids pure entangled states of SA (or other global states not invariant under $U(\pi)_S \otimes \mathbf{1}_A$).

The second option is certainly possible, and hence, we do not claim that a violation of complete invariance will lead to a violation of basic operational properties (such as the non-negativity of probabilities). However, as the analogy above shows, complete invariance is still a very natural and well-motivated property in the context of composition of quantum systems.

The issue of where complete invariance lies on the spectrum of "fundamental principle" versus "convenient strengthening to obtain Bosons/fermions" is important and indeed not fully clarified in the manuscript. Given the above, we would say that it lies at neither end of the spectrum: it is not a strict physical necessity, but a well-motivated condition to postulate. In the main text, we have tried to explain the "compositional" motivation better than in the old version, by writing:

Furthermore, complete invariance expresses a strong compatibility of symmetries and compositions of systems: if we have a system S in our lab, and we know that it is prepared in a completely invariant state (e.g. by measuring the projector onto the Bosonic or Fermionic subspace), we can be sure that nothing else in the universe will change under local permutations, regardless of how S is correlated or entangled with other systems A. Similarly as for complete positivity, a more detailed analysis of this notion could benefit from the category-theoretic methods of process theories, analyzing the compositional structure of quantum theory in a more systematic way.

As a final comment, let us illustrate the operational motivation behind our principle with a comparison to properties of quantum communication protocols, which rely on certain properties of the system being transmitted. In order to enforce maximal security of the protocol, one assumes that the adversary might be correlated with the states being transmitted, i.e. might have access to a purifying system. In this case, an adversary's actions on an intercepted system can involve measurements of the purified system S and ancilla A.

Within this adversarial context, the notion of invariance might be defined via the following task: encode group elements $g \in G$ into states $\rho(g) = U(g)\rho(e)U^{\dagger}(g)$ such that the opponent cannot decode the group element when given access to the states. Assuming that the adversary has access to purifications of the systems used motivates complete invariance, since the use of encodings which statisfy invariance but not complete invariance will allow the adversary to at least partially decode.

In summary, neither this *adversarial* nor the above *compositional* motivation for complete invariance commit one to a pure-state universe perspective, and indeed we believe it is a strength of the proposed principle that it does not require a pure state assumption. Rather, it is a natural strengthening of invariance principles which, as explained above, seems indeed to be satisfied by Nature in many different contexts, including gauge transformations in QFT.

4. Quantum permutations as quantum reference frame transformations

The interpretation of quantum permutations as quantum reference frame (QRF) transformations is conceptually attractive and aligns well with the recent literature. At the same time, it highlights an important subtlety: whether a given transformation is a QRF symmetry operation depends on the type of system.

For bosons and fermions, the authors argue that elements of the appropriate quantum permutation group (V_+ for bosons, V_- for fermions) act as relabelings and should not change physical predictions. For systems where exchanging excitations is itself a physical operation used to implement gates—most prominently non-Abelian anyons—these transformations are not QRF changes but actual dynamical operations.

It would be helpful if the authors could make this distinction explicit in the main text: quantum permutations are QRF transformations only for those particle types for which they preserve all physical predictions; for systems like anyons, the corresponding exchange operations lie outside the symmetry group assumed in this work. This again underlines that the results are about fundamental permutation statistics, not about anyonic braiding.

We agree with the referee's reasoning in the third paragraph of the comment: The corresponding exchange operations for anyons are outside of permutation statistics, so our results do not apply there. However, we think that the distinction between relabellings and physical operations is not as relevant for this point as the referee indicates: both types of transformations are modelled as elements of $\mathcal{V}_+$ or $\mathcal{V}_-$, respectively – and both can either be symmetries of a given particle type or not. For example, in the Roos et al. experiment, active conditional transport operations are elements of $\mathcal{V}_+$, and can be applied to both Bosons and Fermions. These operations turn out to be symmetries for Bosons, not for Fermions. Moreover, passive relabellings according to V_+ are also symmetries for Bosons (and not for Fermions).

We agree with the referee that this point should be clarified. We have thus rewritten the final paragraph in the "Quantum permutation invariance" section (directly after reminding the reader that different types of systems carry different representations of the relevant symmetry groups) as follows:

Hence, elements of $\mathcal{V}_+$ (understood either as active transformations or passive relabellings) are symmetry transformations for Bosons, but not for Fermions (and vice versa for $\mathcal{V}_-$ and

Fermions). Similarly, for other types of systems without fundamental permutation invariance, e.g. anyons, none of the two groups would be symmetry transformations, and our results do not apply.

B. Minor comments

- In the introduction, it may help to briefly recall that in $D \geq 3$ the configuration space of N identical point particles has fundamental group S_N, whereas in 2D it has braid group B_N; this would naturally separate the present focus on S_N-based parastatistics from the anyonic case.

Indeed, we agree that would be a helpful distinction to make. We have added the following paragraph clarifying this at the end of the introduction:

Our results apply to paraparticles defined as fundamental indistinguishable particles transforming under multidimensional irreducible representations of the symmetric group. They do not apply to other important families of particles with exotic exchange statistics such as anyons, which transform under the braid group, and (as we will discuss) they neither rule out emergent parastatistics nor paraparticles where the invariant permutation action does more than just swapping modes, as in graded systems described by Lie superalgebras [Toppan]. On the other hand, our results are rather general because they apply to all kinds of quantum systems that can be permuted, not only to particles with spatiotemporal locations. To this end, our approach defines permutation invariance directly at the level of Hilbert space operators, and not on the level of classical configuration space [Landsman].

- Figures 1 and 2 are clear and useful. It might be worth explicitly stating in the caption of Fig. 2 that the control observable used to define branches must be permutation-invariant.

We agree that it is important to mention this. However, it already says this ("... conditioned on the value of a permutation-invariant observable."), so we thought this would be sufficient. But please let us know if further clarification would be helpful.

- Tables I and II in the Methods section are very helpful summaries of the allowed invariant states and correlations. It could be useful to refer to these tables explicitly in the main text when first stating Theorems 1–3.

We agree this makes sense. A short statement referring to the tables has been added in the main text.

- Since "parafermions" also appear in the condensed-matter literature to denote certain anyonic zero modes rather than Green-type parastatistical fields, a short remark clarifying the terminology would help avoid confusion.

A short remark with references was added in the supplementary material after introducing the notion of parafermions (directly before the start of S2.B.)

C. Conclusion

The manuscript is clearly written, technically sound as far as I can see, and offers a novel, information-theoretic perspective on the absence of fundamental paraparticles. Subject to additions and corrections as elaborated above, I would be inclined to positively reconsider it for publication in Nature Communications. Eventually, the paper could make a valuable contribution to on-going discussions revolving around quantum statistics and/or quantum reference frames.

We thank the referee for their thorough and encouraging assessment of our work.

II. REVIEW REPORT 2

Below, we respond to each comment individually, with the Reviewer's comments in teal and our answers in black.

1 - In the equivalence thesis recalled by the authors, paraparticles are ruled out not due to an inconsistency, but because under, e.g., a strong locality principle, they cannot produce results different from ordinary bosons / fermions. Somehow unexpectedly, the authors' results go in the opposite direction: under complete invariance / quantum permutation invariance, paraparticles are ruled out precisely because they are not equivalent to ordinary bosons / fermions.

At first sight, indeed it seems as if we were doing the "opposite" of what is stated in the equivalence thesis. However, on a more detailed analysis, we believe that both viewpoints are actually closely related: complete invariance, for example, is a strong condition of permutation-invariance for *fundamental* particles. If we have *emergent* parastatistics that comes from Bosons or Fermions with additional degrees of freedom (as described in the equivalence thesis), then these particles will have correlations with those hidden degrees of freedom which *do* change under local permutations. That is, while parastatistical behavior is reproduced locally on the system of interest S (coming from ordinary Bosonic or Fermionic behavior on the global system), there will be non-permutation-invariant correlations or entanglement with those other degrees of freedom A. While we are thus not ruling out the existence of effective or emergent parastatistical behavior, which is distinct from purely Bosonic or Fermionic statistics, we are excluding parastatistics arising from fundamentally indistinguishable particles.

In this sense, our results are consistent with (and arguably sharpen) the equivalence thesis.

In light of Referee 1's second comment, we have added a detailed calculation showing this in the Supplementary Material (based on a paper by Klaas Landsman). Please see our response to this other referee comment for details. This may also help clarify the relation to the equivalence thesis.

2 - The authors feel obliged to comment that their results are not in opposition to the recent tests [14-16] which prove the theoretical detectability of paraparticles. Please, be aware that, in the bibliography, the reference to the first paper presenting a theoretical test with signature of parastatistics is missed. The paper is: - F.T., $\mathbb{Z}_2 \times \mathbb{Z}_2$-graded parastatistics in multiparticle quantum Hamiltonians, J. Phys. A: Math. Theor. 54, 115203 (2021), arXiv:2008.11554[hep-th]. This is the first paper producing a signature of parastatistics (for $\mathbb{Z}_2 \times \mathbb{Z}_2$ parafermions, with ref. [14] being the second paper which extended the test to $\mathbb{Z}_2 \times \mathbb{Z}_2$ parabosons). This paper above should of course be duly cited.

We apologize for the omission, and have now added this citation in the introduction.

3- More mechanisms than the one discussed by the authors in page 5 can in principle be at work to evade the applicability of complete invariance / quantum permutation invariance to rule out paraparticles. For instance, $\mathbb{Z}_2 \times \mathbb{Z}_2$-graded parastatistics implies one-dimensional reps which are organized differently from ordinary bosons / fermions. The refs. [13-16] and the paper above present different mechanisms to evade the applicability of the locality principle. How, in the above cases, complete invariance / quantum permutation invariance is also avoided, requires a careful investigation.

We are grateful for this thoughtful comment, which was very important for us to clarify in detail the applicability of our results and their physical interpretation. Let us first describe the changes to the paper that we have made in response, and then explain some more details. First, also in

response to other reviewer comments, we have added the following paragraph to the end of the introduction in order to clarify the scope of our work:

Our results apply to paraparticles defined as fundamental indistinguishable particles transforming under multidimensional irreducible representations of the symmetric group. They do not apply to other important families of particles with exotic exchange statistics such as anyons, which transform under the braid group, and (as we will discuss) they neither rule out emergent parastatistics nor paraparticles where the invariant permutation action does more than just swapping modes, as in graded systems described by Lie superalgebras [Toppan]. On the other hand, our results are rather general because they apply to all kinds of quantum systems that can be permuted, not only to particles with spatiotemporal locations. To this end, our approach defines permutation invariance directly at the level of Hilbert space operators, and not on the level of classical configuration space [Landsman].

More importantly, we have added the following paragraph to the Conclusions section to give more details on this:

In principle, our mathematical results apply to all unitary representations of the symmetric group, enforcing that $U(\pi)$ acts either trivially or with the sign representation on the physical states. If $U(\pi)$ simply permutes the tensor factors or modes (as assumed above), then the corresponding symmetric and antisymmetric subspaces describe Bosons and Fermions, such that our results rule out parastatistics. However, if $U(\pi)$ is a more exotic representation that e.g. couples the permutations to internal degrees of freedom, then these allowed states may still have an interpretation as particles with exotic exchange statistics. For example, this happens with $\mathbb{Z}_2 \times \mathbb{Z}_2$-graded parastatistics [Toppan], where creation operators are exchanged with a grading-dependent phase $\hat{a}^\dagger \hat{b}^\dagger \mapsto (-1)^{\langle \alpha,\beta \rangle} \hat{b}^\dagger \hat{a}^\dagger$. Such systems transform trivially under exotic permutation actions and are therefore consistent with our principles of quantum permutation invariance and complete invariance as applied to those exotic permutation actions.

This comment has revealed a very subtle interpretational point of our analysis. First, it underlines the generality of our results: demanding *complete invariance* or *invariance under quantum permutations* under some unitary representation of the symmetric group, $\pi \mapsto U(\pi)$, forces the physical states to live in the symmetric or antisymmetric subspace *of that representation*. This is our main mathematical result; and it is very interesting to see that this is fully consistent with $\mathbb{Z}_2 \times \mathbb{Z}_2$-graded parastatistics where physical states $|\psi\rangle$ are indeed required to satisfy $U(\pi)|\psi\rangle = |\psi\rangle$ (in our notation), if we have understood this point correctly.

But, second, the subtlety now arises in the physical interpretation of this result. *If* the representation $U(\pi)$ corresponds to the usual permutation of the tensor factors (in 1st quantization) or to the usual permutation of modes (in 2nd quantization – as in Green's approach, for example), *then* the symmetric and antisymmetric subspaces correspond to Bosons and Fermions, and our extended permutation-symmetry principles rule out parastatistics. But otherwise, it will not in general be naturally interpreted as corresponding to Bosons or Fermions. Indeed, the $\mathbb{Z}_2 \times \mathbb{Z}_2$-grading example demonstrates this clearly, and it is not ruled out by our principles.

In summary, one might say that everything boils down to the questions of what one means by "fundamental particles being invariant under permutations": if the word "permutations" here means the usual, intuitive shuffling of tensor factors or modes, then our results show that "all fundamental particles being [in some strong sense] invariant under permutations must be Bosons or Fermions". Under this reading, one would furthermore like to ask how these permutations are defined *operationally*; and perhaps they would correspond to physical transport operations as in the Roos et al. paper that we have mentioned. However, if one understands the word "permutations" as just "an arbitrary unitary representation of the permutation group", then our results leave room for exotic exchange behavior (perhaps interpreted as "parastatistical"). But the corresponding state vectors will still be invariant up to a sign under these exotic permutations, in contrast to

Green's paraparticles.

By adding the new paragraphs described above, we hope to clarify these important points, and to do so in a way that is succinct enough to fit the structure of this paper and the journal.

4 - In various points the authors rightly question whether the new proposed notions of permutation-invariance are too strong. Could they be relaxed? I have in particular a question based on the example S1 in the Supplementary Material and the comment at the end of Section A. Let's say that we have three particles, A, B, C. One of them, C, is pushed far away. The localized measure only exchanges A and B. Is it indeed true that "the existence of the third particle far away should note influence any measurement in the lab"? What about an EPR scenario? What about if the C particle was in the past entangled to A and B, before being sent far away? Wouldn't be expected, just as in EPR, a correlation due to the past interaction of C with A and B? If so, which would be the consequences of this correlation? I think this point deserves at least some comments.

We agree that some further clarification is helpful here. As a first comment, note that entanglement as in the EPR scenario (say, between the lab system A and a far-away system C) becomes only relevant when measurements on C are performed *and compared to measurement outcomes on* A in order to observe the correlations. When we say that "C is inaccessible", we include in this the assumption that no (classical) data about possible measurement outcomes on C can be accessed in the lab. To clarify this, we have extended a sentence in the last-to-final paragraph of S2.A in the Supplementary Material as follows:

In the same way, by considering the para-sector of three particles as in Example S1 above, we can imagine a scenario where we only have access to two of the particles, and no access whatsoever to the third particle (this includes not having access to the outcomes of potential measurements that some other observer might have performed on the third particle). Since the mere existence of the third particle far away should not influence the measurement outcome statistics in the lab, the accessible pair should therefore either behave as a pair of Bosons or Fermions.

In the case where all three particles are of the same type, let's say Green-type paraparticles as in this example in the paper, the existence of the third particle C far away does not influence the measurable statistics on A and B. The two particles A and B behave just as if there was no particle C, hence they would only exhibit Bosonic/fermionic statistics. As we mention in the paper, this is shown in [2] and generalized to N paraparticles in [3]. In these papers, compatibility with the so-called cluster law is required: two isolated experiments which are sufficiently well separated must not interfere. In essence, any exotic paraparticle-type coming in the form of a pair of particles would not be distinguishable from Bosons and Fermions. We emphasize that this analysis requires the remaining degress of freedom, e.g. the third particle far away, to be inaccessible at all times. In an EPR scenario, any consequences of the correlations between the parties can only be detected once the individual measurements are correlated with each other, thus breaking the separation between them by means of communicating.

Now, in the case of particle C being of a different type, or an arbitrary quantum system altogether (such as a logical qubit in a quantum computer), we do not require fundamental indistinguishability between it and the system (say, AB) of particles in the lab. However, we still require fundamental indistinguishability of the two particles AB in the lab, in the sense of *complete invariance*: swapping A and B shall have no effect on the rest of the world whatsoever. Now, it is clear from the absence of superluminal communication (that is, from the no-signalling principle) that swapping A and B will not change the local state of C. However, it is conceivable that *the correlations between* AB *and* C would change under the swap of A and B, which is what complete invariance forbids. In order to detect such possible changes of state, one would in general have to make joint measurements on ABC, or at least to compare the measurement results on AB and on

C in order to infer the correlations.

5 - On a sideline, it could be quite tempting to understand the possible implications of the refined notions of particle permutations proposed by the authors beyond the permutation group and its higher dim reps labeled by Young tableaux. Here I mean the anyonic parastatistics which is based on the braid group and whose higher-dim reps lead to the important class of non-abelian anyons. This is of course something for the future.

We agree that this is a fascinating line of research questions for future work! It may however be the case that anyons are not quite the next logical case to study, since, as we understand it, one does not in general expect anyons (in particular, *non-Abelian* anyons) to be invariant under braidings (rather, unitary operations are implemented that allow for quantum computation). Therefore, a more natural arena to study are *gauge transformations in quantum field theory* (with groups such as U(1) etc. rather than permutations), since we expect that the physical state of a system is invariant under any gauge transformation (and, hopefully, even completely invariant). Indeed, in partial response to Reviewer 1's comment 3, we have added a short comment to the "complete invariance" section which explains this potential further application of complete invariance.

My final point: this is a very important paper which can have significant implications. It provides a welcome clarification on aspects of parastatistics which are related to different areas of physics (quantum information/ QRF program and more). It introduces refined notions of "invariant permutation of particles". I have no doubt that this paper deserves being published. I urge the author to insert in the bibliography the reference mentioned at the point 2 and, possibly, give some comments and spend some words on the comments/questions 1-5.

Thank you so much for the detailed, thoughtful and encouraging comments, and for many illuminating previous discussions!

III. REVIEW REPORT 3

Below, we respond to each comment individually, with the Reviewer's comments in turqoise and our answers in black.

The topic of the paper is the long-standing fundamental question as to why systems of identical particles observed in Nature are always either symmetric (Bosons) or antisymmetric (Fermions) with respect to permutations of particles. The question arises from the mathematical fact that the representation theory of the permutation group allows multiple irreducible representations, only two of which correspond to Bosonic and Fermionic particles, the rest being called "para-particles".

While existing work on the problem is mainly done in the context of quantum field theory, the present authors propose another approach, which is rather based on an operational approach typical for quantum information theory, and does not require field-theoretic machinery. The authors consider two ways of formulating permutation invariance: (1) the invariance of all results of measurements made on an open quantum system, and (2) invariance of measurement results conditioned on the outcome of a projective measurement. They then proceed to show how either of these assumptions can be used to rule out para-particles. This is the noteworthy result of the paper. Finally the authors link the results to recent research on quantum reference frames.

The authors' approach seems entirely original, and very appealing from the perspective of operational quantum physics, which (unlike much of field theory) is solidly grounded on the theory of prepare-and-measure type experiments. In particular, the formulation of "quantum permutations" is quite neat as it utilises the fundamental concept of quantum superposition and the associated

conditional branching based on measurement outcomes. The work is clearly significant for a broad area of fundamental physics which seeks to understand how the structure of the matter can be understood in the micro-level. The mathematical work is highly nontrivial: the authors study in detail the representation theory of the symmetric group, and show rigorously how the para-particle representations are ruled out by the assumptions. The proofs are given in good detail in the Methods section, with some parts postponed to the supplemental material.

We thank the referee for their thorough assessment of our work and the encouraging feedback. Below, we provide responses to the points that were raised.

Based on this, I will recommend that the paper be published, after the authors consider the following minor points:

1) On page 2, the authors explain the need to formulate particle permutations in terms of modes rather than tensor factors when working in the second quantisation (Fock space). I understand this, but the following justification seems slightly confusing: "In second quantization, there are no tensor factors to permute [...]". Now, a common way of introducing the Fock space is done by arranging all N-particle spaces $\mathcal{H}^{\otimes N}$ into a direct sum, and defining e.g. annihilation operator first on the whole of this space and then projecting to the symmetric (antisymmetric) subspaces to get the usual CCR (CAR) relations. So here we certainly have tensor factors, while the modes only arise after we choose a basis for $\mathcal{H}$. So one might naively think of defining the standard permutations for each N-particle space separately (as in the first quantisation) to get a description for the total Fock space (2nd quantisation). Perhaps the authors could add a brief explanation as to why this does not work.

We agree that the previous formulation was confusing for readers. We have slightly reformulated in the following way:

In second quantization, and in particular in the formalism of Green's paraparticles that we include in our analysis, this tensor product structure is lost, and so are the aforementioned permutations of tensor factors.

This is immediately followed by the earlier sentence: *Indeed, attempts to define analogous transformations lead to non-unitary and non-physical maps (see Section S4 A in the Supplementary Material).*

We agree with the referee's statement that the second quantized picture can be constructed from the tensor algebra $\bigoplus_n V^{\otimes n}$ with V the single particle space, and projecting onto a subspace (symmetric for bosons, antisymmetric for Fermions and other representations for parastatistics). The issue with the parastatistics subspace is that while the relevant subspace will be closed under the particle permutations permuting between the modes, it will not be closed under the permutations of tensor factors. This is shown in [4], where they focus on a discrete number of modes, i.e. $V \simeq \mathbb{C}^d$, and the summand $(\mathbb{C}^d)^{\otimes d}$ where both the place permutations and particle permutations act corresponding to a representation of $S_d \times S_d$. Using tools from representation theory, they show that any subspace of $V^{\otimes d}$ corresponding to parastatistics is closed under the particle permutations but not the "place permutations", i.e. permutations of tensor factors.

In the paper, we use a different way of showing the failure of place permutations directly in terms of the annihilation and creation operators.

Here is why defining these "place permutations" fails in second quantization. This is directly from our paper's appendix (S4.A) and it shows the failure even in the subset of cases we consider: states with exactly one excitation per mode.

While the action of standard permutations in first quantization is given by

$$U(\pi)|x_1,\ldots,x_N\rangle = |x_{\pi^{-1}(1)},\ldots,x_{\pi^{-1}(N)}\rangle, \tag{13}$$

usually referred to as a *label* or *place* permutation, it can generally not be defined consistently this way on Fock states

$$|k_1, ..., k_N\rangle_{\mathcal{F}} := a^\dagger_{k_1} ... a^\dagger_{k_N} |0\rangle_{\mathcal{F}} \tag{14}$$

and the associated creation operators in second quantization. To see this, consider the parastatistical model from Example S2 (Volkov model): There, a product of three creation operators fulfills the relation $a^\dagger_i a^\dagger_j a^\dagger_k = -a^\dagger_k a^\dagger_j a^\dagger_i$ for $i, j, k \in \{1, 2, 3\}$). Given the state $\left(a^\dagger_2 a^\dagger_1 a^\dagger_3 + a^\dagger_2 a^\dagger_3 a^\dagger_1\right) |0\rangle_{\mathcal{F}} \neq 0$, exchanging the positions of the first two creation operators gives $\left(a^\dagger_1 a^\dagger_2 a^\dagger_3 + a^\dagger_3 a^\dagger_2 a^\dagger_1\right) |0\rangle_{\mathcal{F}} = 0$, entailing that place permutations are not unitary. Instead, standard permutations in second quantization, which are referred to as *particle* permutations, act by permuting the *modes*. For example, the particle permutation corresponding to exchanging modes 1 and 2 from above can be represented by a unitary operator $\bar{U}((12))$ that effectively exchanges the creation operators $a^\dagger_1$ and $a^\dagger_2$ and transforms $\left(a^\dagger_2 a^\dagger_1 a^\dagger_3 + a^\dagger_2 a^\dagger_3 a^\dagger_1\right) |0\rangle_{\mathcal{F}}$ into $\left(a^\dagger_1 a^\dagger_2 a^\dagger_3 + a^\dagger_1 a^\dagger_3 a^\dagger_2\right) |0\rangle_{\mathcal{F}} \neq 0$.

2) In the methods section, the proof of Theorem 1 is structured somewhat oddly. In fact, after Lemma 1 the authors state that Thm 1 follows from this Lemma, but the claim of Thm 1 (state being supported in either Bosonic or Fermionic subspace) is not explicitly mentioned in the statement of the Lemma 1 but rather appears as a side consequence in the middle of its proof. Perhaps the authors could improve the structure here.

We agree. We have restructured the lemma by adding an equivalent item (iv) to the other three, saying that ρ_S has full support on the Fermionic or Bosonic subspace, and adapted the proof accordingly. We have done this as well for the generalization of the lemma to compact groups in the supplementary material.

---

[1] N. P. Landsman. Quantization and superselection sectors III: Multiply connected spaces and indistinguishable particles. *Rev. Math. Phys.*, 28(09):1650019, 2016.

[2] James B. Hartle and John R. Taylor. Quantum Mechanics of Paraparticles. *Physical Review*, 178(5):2043–2051, 1969.

[3] Robert H. Stolt and John R. Taylor. Classification of Paraparticles. *Physical Review D*, 1(8):2226–2228, 1970.

[4] R. H. Stolt and J. R. Taylor. Correspondence between the first- and second-quantized theories of paraparticles. *Nuclear Physics B*, 19(1):1–19, 1970.

Invariance under quantum permutations rules out parastatistics

We thank the Referees for the positive assessment, and we are glad that all three Reviewers suggest acceptance. Referee 1 has sent us two comments to be implemented before acceptance. We agree with both of them, and have slightly edited the paper accordingly. Specifically, Reviewer 1 wrote:

1. *The following sentence in the Abstract "we give two independent arguments that rule out parastatistics universally" may give a wrong impression because the authors also write: "They do not apply to other important families of particles with exotic exchange statistics such as anyons, which transform under the braid group, and (as we will discuss) they neither rule out emergent parastatistics nor paraparticles where the invariant permutation action does more than just swapping modes, as in graded systems described by Lie superalgebras".*

   We have reworded the sentence in the abstract, explaining in particular what type of parastatistics is ruled out. The new version of the sentence reads now:

   "Here we give two distinct model-independent arguments that rule out parastatistics, i.e. fundamentally indistinguishable quantum systems transforming under higher-dimensional representations of the symmetric group, which draw on quantum information theory and recent research on internal quantum reference frames."

2. *The reply seems to be appropriately careful in describing complete invariance as a well-motivated additional postulate, rather than a strict necessity. However, the manuscript seems to phrase the step from ordinary invariance to complete invariance too strongly, e.g. by stating that "The assumption that "physics is invariant" under local permutations of S should then imply the condition...". That wording makes the argument sound more compulsory than claimed in the reply letter.*

   We have revised this sentence, and some of those that immediately precede and follow it, to avoid claiming that this is an unavoidable consequence of the "physics doesn't change" intuition. While doing so, we have taken the freedom to slightly improve the presentation in order to clarify the logic of the argument, see the beginning of the subsection"Complete Permutation Invariance" on page 3 in the colored version. Concretely, we first begin with a single realized extension ρ_{SA}; invariance under local permutations is equivalent to equation (5) for this specific extension. The strong assumption of complete invariance is, however, the more general and stronger postulate that this holds for all extensions ρ_{SA}; and then, this turns out to be equivalent to the existence of a single pure extension with this property. We believe that the new formulation makes it clear that we do not claim complete invariance to be a physical necessity, but a well-motivated additional postulate.

Thank you again for the valuable and thoughtful comments.

Kind regards,
Manuel, Thomas and Markus

Review of Manuscript: "Invariance under quantum permutations rule out parastatistics"(corresponding author: M. P. Muller)¨

In the manuscript the authors refine the naive notion of "invariant permutation of particles" by introducing two new invariant notions. The first one, "complete invariance", is inspired by quantum information theory and the notion of complete positiveness. It puts further conditions on a quantum system, requiring not only its local permutation invariance, but also the preservation of information that it carries about some other (ancillary) system. The second notion, "quantum permutation invariance" takes into account permutations which are conditioned on values of observables. The authors are able to link this second notion to the Quantum Reference Frame program which investigates, e.g., quantum versions of the equivalence principle and postulates novel predictions requiring covariance under QRF transformations.

A careful analysis of the physical implications of these two new notions are made. First-quantized complete invariant theories can only be consistent for ordinary bosons / fermions and not for more general higher dimensional reps of the permutation group.
The notion of "quantum permutation invariance" collapses, applied to classical states, to the ordinary permutation-invariant notion. In the quantum case (both first and second quantization) unlike ordinary permutation invariance, "quantum permutation invariance" is only consistent for bosons / fermions, ruling out once more higher dimensional reps of the permutation group. In QRF language this is linked to a lack of a fundamental reference for labeling particles.

The paper is thought-provoking, brilliant and based on a sound mathematics. I found misplaced the stress by the authors on the "ruling out of parastatistics". In my understanding, the proposed refined notions of permutations invariance are the really important key issues. Technically, I cannot object to the title ("Invariance under quantum permutation rule out parastatistics" is correct). On the other hand, I feel that it somehow diminishes the main results: the refinements of the notions of invariant permutations link parastatistics to two broad research programs (in quantum information and, via QRF, quantum gravity). These refinements can suggest new classes of tests investigating, under which conditions, complete invariance and quantum permutation invariance are applicable to given quantum systems.

I have the following comments/questions:

1 - In the *equivalence thesis* recalled by the authors, paraparticles are ruled out not due to an inconsistency, but because under, e.g., a strong locality principle, they

cannot produce results different from ordinary bosons / fermions.
Somehow unexpectedly, the authors' results go in the opposite direction: under complete invariance / quantum permutation invariance, paraparticles are ruled out precisely because they are not equivalent to ordinary bosons / fermions.

2 - The authors feel obliged to comment that their results are not in opposition to the recent tests [14-16] which prove the theoretical detectability of paraparticles. Please, be aware that, in the bibliography, the reference to the first paper presenting a theoretical test with signature of parastatistics is missed. The paper is:

- F.T., *$Z_2 \times Z_2$-graded parastatistics in multiparticle quantum Hamiltonians*, J. Phys. A: Math. Theor. 54, 115203 (2021), arXiv:2008.11554[hep-th].

This is the first paper producing a signature of parastatistics (for $Z_2 \times Z_2$ parafermions, with ref. [14] being the second paper which extended the test to $Z_2 \times Z_2$ parabosons). This paper above should of course be duly cited.

3- More mechanisms than the one discussed by the authors in page 5 can in principle be at work to evade the applicability of complete invariance / quantum permutation invariance to rule out paraparticles. For instance, $Z_2 \times Z_2$-graded parastatistics implies one-dimensional reps which are organized differently from ordinary bosons / fermions. The refs. [13-16] and the paper above present different mechanisms to evade the applicability of the locality principle. How, in the above cases, complete invariance / quantum permutation invariance is also avoided, requires a careful investigation.

4 - In various points the authors rightly question whether the new proposed notions of permutation-invariance are too strong. Could they be relaxed? I have in particular a question based on the example S1 in the Supplementary Material and the comment at the end of Section A. Let's say that we have three particles, A, B, C. One of them, C, is pushed far away. The localized measure only exchanges A and B. Is it indeed true that "the existence of the third particle far away should note influence any measurement in the lab"? What about an EPR scenario? What about if the C particle was in the past entangled to A and B, before being sent far away? Wouldn't be expected, just as in EPR, a correlation due to the past interaction of C with A and B? If so, which would be the consequences of this correlation? I think this point deserves at least some comments.

5 - On a sideline, it could be quite tempting to understand the possible implications of the refined notions of particle permutations proposed by the authors beyond the permutation group and its higher dim reps labeled by Young tableaux. Here I mean the anyonic parastatistics which is based on the braid group and whose higher-dim reps lead to the important class of non-abelian anyons. This is of course something

for the future.

My final point: this is a very important paper which can have significant implications. It provides a welcome clarification on aspects of parastatistics which are related to different areas of physics (quantum information/ QRF program and more). It introduces refined notions of "invariant permutation of particles". I have no doubt that this paper deserves being published. I urge the author to insert in the bibliography the reference mentioned at the point 2 and, possibly, give some comments and spend some words on the comments/questions 1-5.

Francesco Toppan
